# Highly Tunable, Nanomaterial‐Functionalized Structural Templating of Intracellular Protein Structures Within Biological Species

**DOI:** 10.1002/advs.202406492

**Published:** 2024-11-13

**Authors:** Dae‐Hyeon Song, Chang Woo Song, Seunghee H. Cho, Tae Yoon Kwon, Hoeyun Jung, Ki Hyun Park, Jiyun Kim, Junyoung Seo, Jaeyoung Yoo, Minjoon Kim, Gyu Rac Lee, Jisung Hwang, Hyuck Mo Lee, Jonghwa Shin, Jennifer H. Shin, Yeon Sik Jung, Jae‐Byum Chang

**Affiliations:** ^1^ Department of Materials Science and Engineering Korea Advanced Institute of Science and Technology Daejeon 34141 South Korea; ^2^ Department of Mechanical Engineering Korea Advanced Institute of Science and Technology Daejeon 34141 South Korea; ^3^ Department of Biological Sciences Korea Advanced Institute of Science and Technology Daejeon 34141 South Korea; ^4^ Bioimaging Data Curation Center Seoul 03760 South Korea

**Keywords:** biotemplating, nanomaterials, nanostructures, surface‐enhanced Raman spectroscopy (SERS) substrate, tunability

## Abstract

Inside living organisms, proteins are self‐assembled into diverse 3D structures optimized for specific functions. This structure‐function relationship can be exploited to synthesize functional materials through biotemplating and depositing functional materials onto protein structures. However, conventional biotemplating faces limitations due to the predominantly intracellular existence of proteins and associated challenges in achieving tunability while preserving functionality. In this study, Conversion to Advanced Materials via labeled Biostructures (CamBio), an integrated biotemplating platform that involves labeling target protein structures with antibodies followed by the growth of functional materials, ensuring outstanding nanostructure tunability is proposed. Protein‐derived plasmonic nanostructures created by CamBio can serve as precise quantitative tools for assessing target species is demonstrated. The assessment is achieved through highly tunable and efficient surface‐enhanced Raman spectroscopy (SERS). CamBio enables the formation of dense nanogap hot spots among metal nanoparticles, templated by diverse fibrous proteins comprising densely repeated monomers. Furthermore, iterative antibody labeling strategies to adjust the antibody density surrounding targets, amplifying the number of nanogaps and consequently improving SERS performance are employed. Finally, cell‐patterned substrates and whole meat sections as SERS substrates, confirming their easily accessible, cost‐effective, scalable preparation capabilities and dimensional tunability are incorporated.

## Graphical abstract

This work demonstrates an integrated biotemplating that utilizes intracellular proteins to fabricate highly functional nanomaterials, called Conversion to advanced materials via labeled Biostructures (CamBio). This work exploits the structural characteristics of cells and tissues, employing them as Surface‐Enhanced Raman Spectroscopy (SERS) substrates by combining conventional fabrication and bio‐related techniques. This work offers the capability to transform advanced materials by adding biostructural benefits for a myriad of applications.

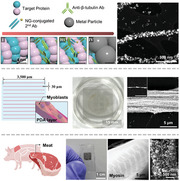

## 1 Introduction

In nature, biological structures span from viruses to animal tissues, and these structures are primarily comprised of various proteins.^[^ ^1^ ^]^ The proteins assemble within biological structures, giving rise to well‐defined hierarchical arrangements.^[^ ^2^ ^]^ Over an extensive period, these structures have evolved to exhibit distinctive 3D geometries for specific functions and display sophisticated nanostructures that challenge artificial synthesis methodologies.^[^ ^3^ ^]^ Therefore, researchers have dedicated various efforts to leveraging the inherent structure‐function correlation in these biological structures.^[^ ^4^ ^]^ Biotemplating, a representative method, synthesizes and organizes inorganic nanostructures using biostructures as templates, resulting in well‐defined architectures with technological significance.^[^ ^5^ ^]^ By leveraging the structural advantages of biological systems, this process enables the creation of novel micro/nanostructured materials.^[^ ^6^ ^]^

However, biotemplating methods face 2 critical challenges in deriving structure‐function correlation from biostructures. First, utilizing specific intracellular protein structures within complex biosystems is challenging as current approaches mainly use either their outer surface or overall morphologies.^[^ ^7^, ^8^ ^]^ While other studies have successfully used the interactions of peptide materials in organisms with simple genes, such as viruses, to fabricate inorganic structures, more intricate biosystems, such as cells, tend to use the outer surface or overall morphologies as templates rather than specific intracellular protein structures. Since structurally distinctive and practically functional biological structures primarily exist intracellularly, harnessing intracellular structures is crucial for exploiting nanostructure‐property relationships.^[^ ^9^ ^]^ Second, current biotemplating methods are limited in terms of the scalability of structure features and overall sample size. Depositing materials onto the morphology of biostructures can encounter challenges in scale tunability because most biotemplates are pre‐established with fixed dimensions.^[^ ^10^ ^]^ Additionally, large‐scale fabrication using viruses or recombinant biomolecules may face issues with insufficient purified material or biotemplate morphological failures that hinder long‐range order.^[^ ^11^ ^]^ However, employing controlled cultures of cells or tissues with the macroscopic organization of various biomolecules lays the foundation for massive production, offering a feasible solution for large‐scale fabrication.

To address the challenges encountered in biotemplating methods while preserving structural benefits, we propose an integrated biotemplating approach called Conversion to advanced materials via labeled Biostructures (CamBio). Compared to existing biotemplating methods, CamBio can utilize specific intracellular structures through a labeling process. Additionally, it offers high tunability by integrating other techniques, even when biotemplates are used (Table S1, Supporting Information). We have recently reported studies that combine antibody labeling and material growth on specific proteins in cells/tissues for catalysts, bioimaging, and sensors.^[^ ^12^, ^13^, ^14^ ^]^ Herein, we exploit the structural characteristics of cells and tissues, exemplified by employing them as Surface‐Enhanced Raman Spectroscopy (SERS) substrates. Moreover, to enhance tunability in biostructures and biotemplating, we introduce iterative antibody labeling strategies and verify the altered characteristics using electron microscopy and SERS performance. Furthermore, we demonstrate the use of Induced Coupled Plasma‐Reactive Ion Etching (ICP‐RIE) as a physical etching to eliminate unnecessary biomolecule layers, thereby exposing the underlying converted structures and enabling subsequent surface modification. Lastly, CamBio can combine cell patterns that extend beyond a single cellular level. Moving to the tissue level, we demonstrate that integrating cryo‐sectioning with CamBio enables the exploration of complex internal biostructures within tissues, which are not observable in individual cells.

## 2 Results and Discussion

### 2.1 Conversion to Advanced Materials via Labeled Biostructure (CamBio)

**Figure 1:**
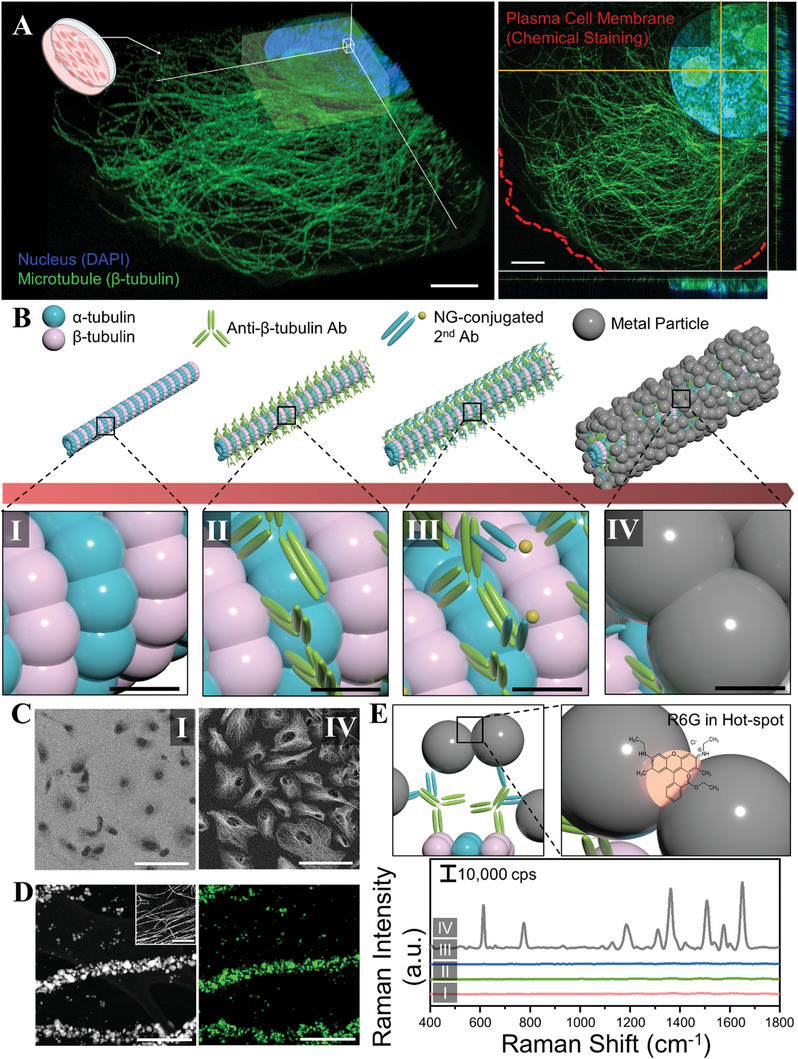
Schematic of the CamBio process from a target protein structure. A) 3D volumetric expansion microscopy image of a beta‐tubulin stained cell. The expansion factor is 4.309 (Figure S1, Supporting Information). B) Step‐by‐step process of intra‐cellular biostructure templating using CamBio. (B.I) Fixed cell state (target protein structure: microtubule). (B.II) Primary antibody (Ab) stained state. (B.III) Nanogold‐conjugated secondary Ab stained state. (B.IV) Metal‐grown state (silver, Ag). C) SEM images of cell substrates corresponding to each step illustrated in B.I and B.IV. D) TEM (left) and EDS mapping (right) images of silver nanoparticle (AgNP) chains converted from microtubule structure via CamBio (Inset image: low‐magnification image of TEM, scale bar: 2 µm). E) Illustration of a hot spot in AgNPs chain, SERS, and Raman analysis of cell substrates from each step using R6G, average spectra, *n* = 10 points, each point from ten independent cells in a single substrate. Scale bar. A. 5 µm, B. 4 nm, C. 100 µm, D. 300 nm (Inset. 2 µm).

The first step in CamBio is selecting a target protein structure. Here, we choose one of the major cytoskeletons: microtubules. These proteins span the entire cell and provide pathways for motor proteins (**Figure** **1**A; Video S1, Supporting Information).^[^ ^15^ ^]^ Microtubules comprise repetitive self‐assembled alpha‐ and beta‐tubulin dimers (Figure 1B(I)).^[^ ^16^ ^]^ Next, tubulins are labeled using antibodies. They are first labeled with a primary antibody (Figure 1B(II)), followed by a 1.4‐nm gold nanoparticle (AuNPs)‐conjugated secondary antibody that binds to the primary antibody (Figure 1B(III)). Finally, metal particle growth from AuNPs on the labeled biostructures facilitates their conversion into advanced materials (Figure 1B(IV)). The metal growth mechanism involved the catalyzed reduction of metal ions from 1.4‐nm AuNPs, minimizing non‐specific growth and aggregation in unintended areas.^[^ ^17^ ^]^ Consequently, silver growth converted microtubules to silver nanoparticle (AgNP) chains within cells.^[^ ^18^, ^19^ ^]^ AgNP light absorption validated the conversion of microtubules. Before silver growth, the structure was invisible in brightfield microscopy but became visible afterward due to the absorption of light by AgNPs (Figure S2, Supporting Information). The growth of AgNPs was also confirmed by signals observed under fluorescence microscopy (FM) (Figure S3, Supporting Information). As is well known, changes in the fluorescence signal were observed due to the plasma‐enhancing effect of AgNPs positioned near fluorescent molecules (Figures S4 and S5, Supporting Information).^[^ ^20^, ^21^ ^]^ Furthermore, AgNPs in biological specimens increase electron density within the sample, enabling the verification of the structure conversion through electron microscopy.^[^ ^22^ ^]^ Further, scanning electron microscopy (SEM) showed no microtubule features before silver growth. However, following silver growth, AgNP chains were observed along the microtubules (Figure 1C; Figures S6 and S7, Supporting Information). Through transmission electron microscopy (TEM) imaging and energy‐dispersive X‐ray spectroscopy (EDS) mapping, the silver growth along intracellular microtubules was also confirmed (Figure 1D; Figure S8, Supporting Information).

CamBio's primary goal is correlating the structural benefits of biological structures with their functions. Positioning metal particles within densely assembled linear microtubules forms AgNP chains with numerous nanogap hot spots, amplifying local electromagnetic fields for effective SERS substrates.^[^ ^23^ ^]^ Among novel metal particles commonly used in SERS application, AgNPs exhibit strong plasmon resonance, making silver growth the preferred choice for fabricating SERS substrates in this study.^[^ ^24^ ^]^ Hence, SERS/Raman analysis with rhodamine 6G (R6G) successfully detected R6G‐specific fingerprints at the locations of microtubules only after the formation of AgNP chains through silver growth, taking advantage of the hot spots formed within the aligned microtubule structures (Figure 1E; Figure S9 and S10, Supporting Information).^[^ ^25^ ^]^ The detection limit was 5 × 10^−8^ M with an enhancement factor of 1.79 × 10^7^, comparable to other biotemplated SERS substrates (Figure S11, Supporting Information).^[^ ^26^, ^27^, ^28^ ^]^ Unlike typical biotemplating that utilizes external morphologies of biological specimens, CamBio enables specific conversions only on labeled biostructures (Figure S12, Supporting Information). When the growth of gold, another representative material in the SERS application, was introduced, similar outcomes were observed, including comparable optical properties and SERS performance that led to silver growth. (Figure S13, Supporting Information).^[^ ^29^, ^30^ ^]^ Additionally, unlike silver growth, in the case of gold growth, the results indicated that the converted nanostructures exhibited biocompatibility when live cells were cultured onto the substrates following material growth (Figure S14, Supporting Information). The biocompatibility of these structures confirms that CamBio‐processed structures could be used in future biological applications.

### 2.2 Iterative Labeling Strategies For Biostructure‐Tunability

**Figure 2:**
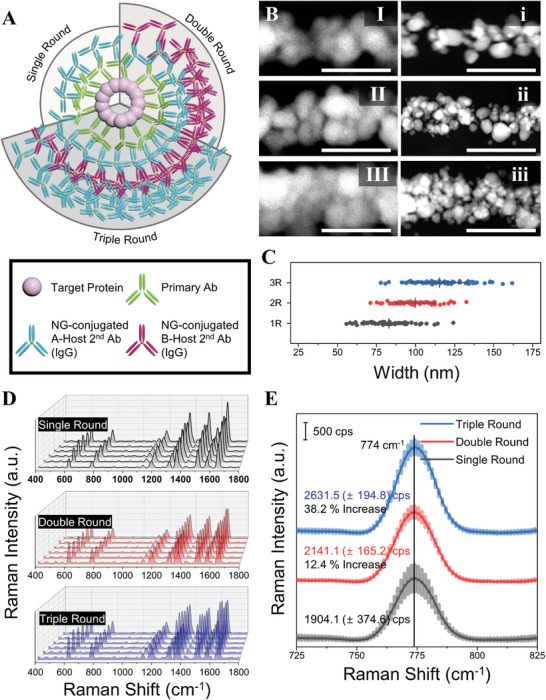
Iterative labeling strategy for structure‐tunability in CamBio. A) Schematic of an iterative labeling strategy from single round to triple round through the introduction of nanogold‐conjugated secondary Ab pair. B) (I‐III) SEM, (i‐iii) TEM images of single AgNP chains converted from microtubules after each round. C) Width comparison of AgNP chains via full width at half maximum value calculation against the labeling round. (average width; 1R: 82.8 nm / 2R: 99.7 nm / 3R: 115.3 nm) Data of each step are presented as mean ± s.d. with measurements from more than *n* = 50 profiles at 2 independent regions of different cells in a single cell culture well. D) Reproducible SERS spectra of R6G on silver‐grown cell substrates according to the number of labeling rounds. E) Raman intensity comparison at a representative peak (774 cm^−1^) with average SERS spectra of each labeling round. SERS spectra data (D, E) are presented as mean ± s.d., total *n* = 6 points taken from 3 cells each from 2 substrates. Scale bar. B. 200 nm.

Inspired by the layer‐by‐layer method, we implemented an iterative antibody labeling technique as part of CamBio's labeling step to tune the biostructure scale.^[^ ^31^ ^]^ The iterative labeling method is commonly employed in bioimaging to amplify the fluorescence signals of target molecules.^[^ ^32^, ^33^ ^]^ As shown in **Figure** **2**A, iterative labeling uses pairs of secondary antibodies. With each round, the total antibody layer increased, allowing the original biostructure to maintain its shape while increasing in size. After multiple labeling rounds and material growth, electron microscopy imaging showed the converted structures tuned from the original form. As shown in the SEM and TEM images, the iteratively labeled and silver‐grown microtubules exhibited a gradual increase in the width of the AgNP chains according to the labeling rounds (Figure 2B; Figure S15, Supporting Information). Comparing the widths of AgNP chains using full width at half maximum from SEM reveals that the average width increased from 82.4 nm after a single round to 115.3 nm after 3 rounds (Figure 2C; Figure S16, Supporting Information).

Each labeling round increased the number of AuNP seeds, resulting in more hot spots within each AgNP chain. Due to size tuning occurring only in the labeled microtubules, even with repeated labeling, the AgNP chains exhibited high reproducibility (Figure 2D) and signal enhancement in SERS performance (Figure 2E) with a 38% increase when comparing single to triple rounds. Furthermore, electromagnetic simulations confirmed the SERS signal enhancements with each labeling round by showing an increase in hot spots (Figure S17, Supporting Information). The tunability of biostructures through iterative antibody labeling varied depending on different strategies based on the choice of antibodies used during the labeling process. Using smaller antibodies, such as the fragment antigen binding (Fab') region as secondary antibodies, instead of the whole immunoglobulin secondary Ab pair previously used, as shown in Figure 2, allows easier penetration into the biostructure and provides the potential for more precise size tuning. We employed 2 different fluorophore‐conjugated Fab' pairs in odd and even rounds to iteratively tune each material. This iterative staining was confirmed by the distinct increases in fluorescence signals from each fluorophore observed in each round (Figure S18, Supporting Information). Moreover, successful silver growth in cells corresponding to each round was observed through brightfield imaging (Figure S19, Supporting Information). Another strategy involves using non‐conjugated antibodies in one of the secondary antibody pairs, allowing for a volumetric increase in hot spots and SERS enhancement with reduced use of 1.4‐nm gold‐conjugated antibodies (Figure S20, Supporting Information). Employing a nonuple‐round labeling approach led to a remarkable increase of 230% in SERS signals obtained from the converted AgNP chains (Figure S21, Supporting Information).

### 2.3 Converted Structure Extraction Through Membrane Removal

**Figure 3:**
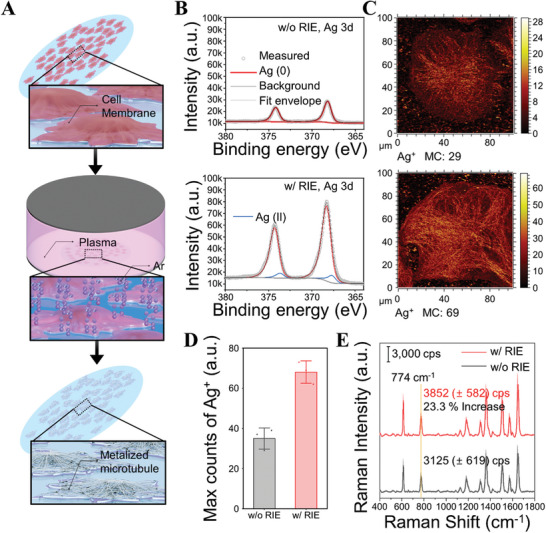
Extraction of the converted structure via CamBio through the membrane removal process. A) Schematic of the membrane removal process by introducing ICP‐RIE. B) Ag 3d XPS spectra of silver‐grown cell substrate before and after RIE. (top: w/o RIE, bottom: w/ RIE). C) ToF‐SIMS mapping images of silver‐grown cells according to the RIE process. D) Max counts comparison of silver ion against RIE (average max count, MC (a.u.); without: 35 / with: 68). Data of each sample are presented as mean ± s.d., *n* = 3 from 3 independent regions in a single substrate. E) Average SERS spectra of R6G recorded on silver‐grown cell substrates with and without the RIE process. Data are presented as mean ± s.d., *n* = 7 points from 7 independent cells in a single substrate.

The cell membrane covering the converted protein structures can hinder SERS performance by creating gaps that prevent analyte adsorption onto metal surfaces. Therefore, a method was devised to remove them. ICP‐RIE is widely used to etch various materials to eliminate unnecessary layers during fabrication.^[^ ^34^, ^35^ ^]^ Physical etching using ICP‐RIE resulted in the extraction of converted structures, as shown in **Figure** **3**A, eliminating unintentional biomaterial barriers.

X‐ray photoelectron spectroscopy (XPS) analysis verified structure extraction through the RIE process. The examination of the Ag 3d XPS spectra of cell substrates with AgNP chains converted along microtubules revealed significantly higher intensity with RIE than without RIE (Figure 3B). This suggests an effective removal of biomaterials, such as the cell membrane covering the converted structures. In physical RIE etching, determining suitable processing time under consistent induced voltage is vital.^[^ ^36^ ^]^ A comparison of Ag 3d XPS spectra at different RIE times showed the highest Ag 3d peak intensity at 20 s, while 30 s resulted in damage to the AgNPs (Figure S22, Supporting Information). In the case without RIE, the spectra were almost identical after 10 s of processing due to biomaterial surrounding the AgNPs reducing surface oxidation and minimizing the observation of the Ag^1+^ ion peak (Figure S23, Supporting Information).^[^ ^37^ ^]^ At 20 and 30 s, Ag^1+^ ion peaks appeared, and after 30 s, overall Ag 3d intensity decreased with the increased Ag^1+^ peak; this indicated that structural damage could occur beyond a certain etching time. Additionally, the analysis of time‐of‐flight secondary ion mass spectroscopy (ToF‐SIMS) ensured a precise morphological structure extraction match. ToF‐SIMS provided detailed surface composition information and high spatial resolution for converted structure information at the cellular level.^[^ ^38^ ^]^ At the single‐cell level, silver ion images with and without RIE showed more distinct observations of AgNP chains converted along microtubules (Figure 3C; Figure S24, Supporting Information). Furthermore, comparing the maximum silver ion count through ToF‐SIMS imaging revealed a two‐fold difference, visually confirming the removal of biomaterial covering the converted structures (Figure 3D).

From the perspective of SERS substrates, removing the incidental layer between analytes and the novel metal surfaces provided advantages in analyte adsorption. The Raman spectra of the silver‐grown cell substrates, both without and with RIE, reveal a clear difference. Without RIE, the biomolecules inherent in the cells introduced noise, whereas, with RIE, many of those noise peaks were removed (Figure S25, Supporting Information). Comparison of SERS spectra through R6G molecules revealed that the intensity of an RIE‐processed substrate increased by ≈23% (Figure 3E), attributed to enhanced analyte absorption on the metal surface from cell membrane removal. In addition, we demonstrated the effect of RIE on gold‐grown cell substrates. Unlike silver, chemically stable gold showed no surface oxidation peaks in XPS analysis after RIE (Figure S26, Supporting Information). Nonetheless, the Au 4f XPS peak intensity was high, and ToF‐SIMS imaging revealed a higher maximum gold ion count with RIE, similar to the results observed for silver (Figure S27, Supporting Information). While Raman spectra showed no significant differences due to gold's lower surface plasmon resonance effect than that of silver (Figure S28A, Supporting Information),^[^ ^39^ ^]^ the SERS signal intensity through R6G demonstrated a higher difference with RIE (Figure S28B, Supporting Information). Furthermore, after membrane removal, an additional function was incorporated through thiol functionalization onto the converted gold structures, utilizing gold's higher thiol affinity than silver (Figure S29, Supporting Information).^[^ ^40^ ^]^ Thiol‐functionalized bisphenol A (BPA) aptamers enabled successful BPA detection, thus confirming the potential to expand the application of converted structure through CamBio.^[^ ^41^, ^42^, ^43^, ^44^ ^]^

### 2.4 Tunability with Patterned Cells in CamBio

Adherent cells proliferate in response to the given environment.^[^ ^45^ ^]^ When sufficient nutrients are supplied, cells can autonomously cover the designated cell culture area comprehensively.^[^ ^46^ ^]^ Leveraging these traits with CamBio, we can obtain substrates covering areas using culture dishes of different diameters (Figure S30, Supporting Information). This method offers a solution to the large‐scale production challenges posed by traditional biotemplating approaches.

**Figure 4:**
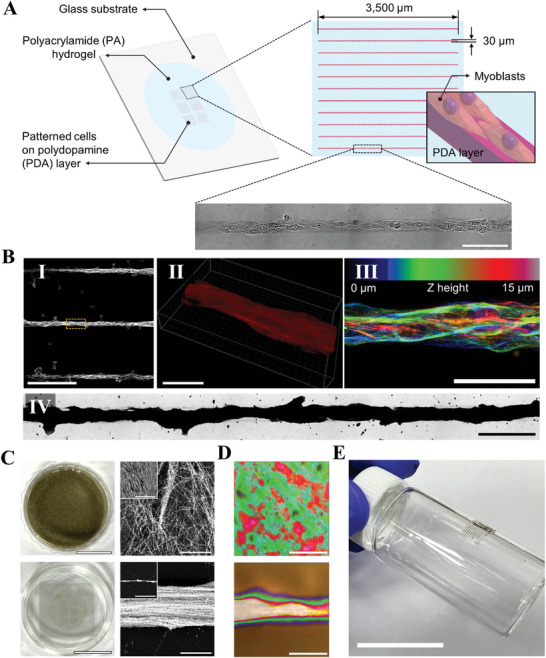
Combination of CamBio with a cell patterning technique. A) Schematic of linear cell patterning using myoblasts on a hydrogel substrate. B) FM images of microtubule‐labeled linear patterned myoblasts. (I) Maximum intensity projected FM images. (II) 3D blend image of the yellow box region in I. (III). Z‐color‐coded map image in the yellow box region. (IV) Brightfield image of silver‐grown line‐patterned cells at labeled microtubule structures. C) Images of silver‐grown cell substrates. A cell substrate without patterning is shown at the top line, and one with patterning is shown at the bottom line. (Left images: digital image, right images: SEM images, inlet image: low‐magnified SEM images) D) Raman mapping images of R6G recorded on cell substrates (intensity at 1362 cm^−1^). E) A digital image of flexible cell patterned SERS substrate. Scale bar. A. 100 µm, B.I. 300 µm, II. 30 µm, III. 50 µm, IV. 100 µm, C. 15 mm (digital), 5 µm (SEM, Inset. 200 µm), D. 20 µm, E. 25 mm.

Recent strategies control cell‐surface interaction by adjusting micro‐environmental factors such as substrate stiffness or geometry patterning to culture cells in 2D patterns.^[^ ^47^ ^]^ In particular, the cell patterning technique is widely utilized in cell behavior studies, tissue engineering, drug screening, and more.^[^ ^48^ ^]^ Herein, to demonstrate the scalable tunability of biotemplating by combining cell patterning and CamBio, we produced polyacrylamide substrates on which C2C12 myoblast was patterned in a micro‐scale pattern. (**Figure** **4**A; Figure S31, Supporting Information). Despite the micrometer‐scale patterning, labeling of patterned C2C12 cells confirmed the acquisition of linear nanoscale microtubule orientation (Figure 4B(I‐III)). FM images showed that in the absence of patterning, the cells exhibited random, non‐directional morphology with disorganized microtubule alignment; however, when patterned, they exhibited a well‐oriented microtubule alignment (Figure S32, Videos S2, and S3, Supporting Information). Silver growth on these patterned cells yielded millimeter‐scale linear AgNP chains (Figure 4B(IV)), demonstrating the potential for tunable, large‐scale biotemplating. Without patterning, digital images of silver‐grown cell substrates revealed that the entire dish appeared uniformly covered, similar to previous cultures of epithelial cells. With patterning, the silver‐grown regions were confined to the hydrogel pattern areas (Figure 4C). Unlike epithelial cells, C2C12 myoblasts without patterning tend to spread and differentiate randomly. Additionally, SEM images revealed AgNP chains following the random orientation of the cells, showing layers of differentiated and undifferentiated cells (Figure S33, Supporting Information). In contrast, with patterning, there was a discernible alignment of microtubules, resulting in uniformly oriented AgNP chains.

The ability to tune cell shape and orientation within the substrate through patterning is reflected in the application of the CamBio‐converted structures. An examination of the SERS substrate with microtubule structures revealed significant SERS intensity variance within the cells in the absence of patterning, indicating spot‐to‐spot variation in Raman mapping with R6G (Figure 4D; Figure S34, Supporting Information). Conversely, patterning produced a homogenous SERS response within the patterned zones due to the consistent microtubule alignment in those cells. Moreover, SERS spectra comparisons of single AgNP chains from patterned and non‐patterned areas confirmed that the patterning process does not affect the SERS performance (Figure S35, Supporting Information). In addition, cell patterning is versatile; specific applications can employ various patterns based on cell type.^[^ ^49^ ^]^ For instance, we fabricated circular‐patterned SERS substrates with epithelial cells through CamBio, demonstrating the potential for distinct region substrates (Figure S36, Supporting Information). Additionally, cell patterning can be achieved on non‐flat surfaces.^[^ ^50^ ^]^ Applying patterning to curved hydrogel surfaces broadens the scope, facilitating the development of flexible SERS substrates (Figure 4E).

### 2.5 Expanding CamBio Through Tissue Samples

**Figure 5:**
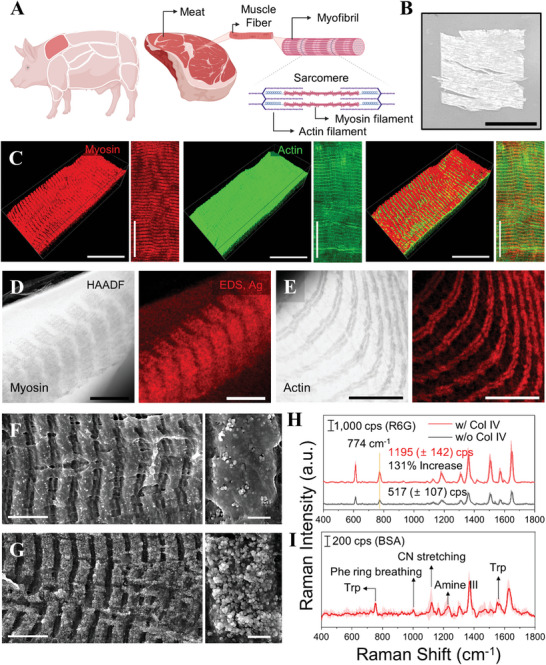
Combination of CamBio with cryo‐sectioned meat slice samples. A) Protein structures in muscle tissues from meats (pork shoulder). B) Digital images of a meat slice substrate (8 mm × 8 mm × 20 µm). C) FM images of myosin and actin in the meat slice (pork shoulder) substrate. (Left: normal shaded, right: maximum intensity projected). TEM images of periodic patterned AgNPs against D) myosin and E) actin proteins (left: high‐angle annular dark‐field image, right: Ag EDS mapping image). SEM images of CamBio processed myosin structures from F) the meat slice sample itself and G) the collagenase‐treated meat slice sample. H) Comparison of average SERS spectra of R6G depending on collagenase treatment. Data of each sample are presented as mean ± s.d., total *n* = 7 points from a single substrate each. I) Average SERS spectra of bovine serum albumin recorded on the collagenase‐treated, silver‐grown meat slice substrate (target protein: myosin). Data are presented as mean ± s.d., *n* = 7 points from different points in a single substrate. Scale bar. B. 5 mm, C. 30 µm, D, E. 5 µm, F, G. 5 µm (left), 500 nm (right).

To demonstrate CamBio's scalability, protein structures within more complex tissues than cultured cells were utilized. Utilizing tissues increased the exploitable protein structure diversity and available template dimensions. Among various tissues, we selected muscle tissue, which is formed through the differentiation of myoblasts.^[^ ^51^ ^]^ FM images of myoblasts revealed abundant fibrous myosin and actin, and we demonstrated the potential of the SERS substrate through silver growth on labeled myosin (Figure S37, Supporting Information). Muscle tissue is easily identifiable within meats, and we selected pork shoulder as a template from various types of meat. Muscle comprises muscle fibers organized into myofibril bundles. Myofibrils, minimal functional units, contain sarcomeres with periodic myosin and actin patterns, which are distinctly different from the disorganized patterns observed in myoblasts (**Figure** **5**A).^[^ ^52^ ^]^ The adoption of cryo‐sectioning, used for analyzing highly aligned bulk structures, allows cost‐effective production of myofiber templates with relatively large dimensions, achieving a thickness of tens of microns (Figure 5B; Figure S38, Supporting Information).^[^ ^53^ ^]^ FM imaging of the cryo‐sectioned meat substrates, with myosin labeled using antibodies and actin labeled with phalloidin, enabled the observation of proteins with distinct patterns in the tissue (Figure 5C).^[^ ^54^, ^55^, ^56^ ^]^ Furthermore, the structural patterns of myosin and actin in muscle tissue were consistently observed in beef and chicken (Figure S39, Supporting Information).

When silver growth was conducted through CamBio on meat slice samples with a highly periodic pattern, TEM imaging revealed periodic AgNP band patterns (Figure 5D,E). During TEM imaging with a high‐angle annular dark field detector, a contrast inversion phenomenon was observed, attributed to electron scattering influenced by muscle fiber thickness (Figure S40, Supporting Information).^[^ ^12^ ^]^ However, successful silver growth and AgNP formation were confirmed through EDS analysis (Figure S41, Supporting Information). Muscle fibers, densely arranged myofibril bundles, comprise complex actin and myosin proteins with fasciae, consisting of collagen proteins network.^[^ ^57^ ^]^ Like cell membranes, fascial structures are unnecessary layers covering converted structures in the cryo‐sectioned meat slices. To selectively remove the fascial layer, samples were treated with a collagenase solution before labeling the protein structure.^[^ ^58^ ^]^ The treated meat slices exhibited higher myosin antibody labeling density and sharper, more precise periodic AgNP bands on SEM imaging after the silver growth (Figure 5F,G).

The silver‐grown meat slice substrates were used as the SERS substrates, prepared with and without collagenase treatment. Initially, to verify the SERS performance in the AgNP‐converted meat sample within myosin structures, Raman and SERS spectra using R6G were observed (Figure S42, Supporting Information). The SERS performance of the substrate was successfully validated by confirming negligible Raman signals from the substrate compared to the R6G SERS spectra. In the SERS performance of the substrates with and without collagenase treatment, the treated case exhibited an enhancement of 131% as compared to the untreated cases (Figure 5H). Collagenase treatment suggests a similar effect as cell membrane removal observed in the previous RIE process, enhancing analyte absorption. However, compared to the RIE treatment on meat slice samples, collagenase treatment appears more effective as it facilitates the volumetric removal of unnecessary collagen layers, whereas RIE affects only the thin surface layer (Figure S43, Supporting Information). Additionally, the actin structure was well‐preserved in the converted meat slice samples, making them suitable for the SERS application (Figure S44, Supporting Information).^[^ ^54^ ^]^

Biotemplated SERS substrates utilizing innate biological structures could be useful for biomolecule analysis. Biological analytes, such as DNA, proteins, and cells, pose critical challenges for Raman spectroscopy due to their inherently small Raman cross‐sections and the need for substantial SERS enhancement.^[^ ^59^ ^]^ In addition, their large molecular size complicates conformal adherence to the plasmonic surfaces often patterned on a nanometer scale, failing to utilize hot spots.^[^ ^60^ ^]^ Silver‐grown meat slice substrates leverage the densely formed myofibril morphology and strong Ag plasmonic resonance to enable efficient biomolecule analysis, as demonstrated by the detection of distinct bovine serum albumin (BSA) Raman bands (Figure 5I; Figure S45, Supporting Information). BSA is commonly used in immunolabeling to reduce the non‐specific binding of antibodies.^[^ ^61^ ^]^ Therefore, CamBio's substrates, based on biotemplates such as cells or tissues, offer advantages in BSA adhesion, with their capabilities being evident when compared to commercial SERS substrates (Figure S46A, Supporting Information). Furthermore, hierarchically structured meat slices grown with silver exhibited greater overall SERS enhancement of BSA molecules compared to the enhancement observed from silver‐grown cell substrates. (Figure S46B, Supporting Information).

Beyond the application of SERS substrates, the lamellar structures formed by the repetitive organization of myosin and actin in muscle tissue can be utilized to position various materials or control composition using CamBio. To demonstrate this, we introduced gold toning onto AgNPs. Gold toning is widely used in the electron microscopy imaging of biological specimens to prevent the degradation of silver during osmium tetroxide fixation (Figure S47, Supporting Information).^[^ ^62^ ^]^ Antibody labeling and silver growth were first performed on actin, followed by antibody labeling and gold growth on myosin. Although actin and myosin were structurally overlapped, resulting in overall silver EDS readings, we successfully created substrates with Ag‐Au core‐shell particles grown on actin and AuNPs on myosin. Considering the reduction potential, successive labeling and material growth, or the use of conjugated antibodies with different materials, could expand CamBio, enabling the creation of multi‐material converted structures.^[^ ^63^, ^64^ ^]^

## 3 Conclusion

In this study, we demonstrated the conversion to advanced materials via labeled biostructures, leveraging the structure‐function relationship of specifically labeled intracellular protein structures. We showcased exceptional tunability in CamBio, which involves target structure preparation, labeling, and materials growth, along with the application of SERS substrates.

Similar to the conventional layer‐by‐layer (LbL) method, we demonstrated that an iterative antibody staining, a signal amplification approach used in bioimaging, enables scale tunability while preserving the original shapes. Additionally, to better utilize the converted intracellular structures, we proposed a method to remove unnecessary biomolecules covering the converted structures through ICP‐RIE physical etching. Lastly, from the standpoint of biological sample preparation, we achieved customized biotemplates by adjusting the overall shape and dimensions through cell patterning techniques, cryo‐sectioning of biological tissues, and integrating these methods with the CamBio approach.

CamBio harbors the potential for future applications by integrating conventional fabrication and biotechniques. Combining state‐of‐the‐art biotechniques, including gene editing or 3D bioprinting, could revolutionize the utilization of substrates with even more diverse structural benefits.^[^ ^65^, ^66^ ^]^ In the labeling step, tunability can be enhanced through signal amplification methods using DNA or horseradish peroxidase (HRP),^[^ ^67^, ^68^ ^]^ diverse conjugation chemistries, peptides, and genetic tag expression.^[^ ^69^, ^70^ ^]^ Therefore, CamBio has significant potential to drive the evolution of biotemplating methodologies and inspire researchers working with biological structures. This technique can enhance advanced materials by integrating structural benefits for various applications. For instance, blood vessels and the extracellular matrix can be applied where large surface areas are required, such as energy storage or catalytic applications. Highly hierarchical biostructures, exemplified by bones, can be utilized in engineering materials that demand numerous mechanical properties. Moreover, CamBio fabricates structures using biological organisms, potentially opening up new possibilities for functional biomaterial applications.

## 4 Experimental Section

### Chemicals and Materials

The Information about the chemicals, materials, and antibodies used in this study was summarized in Tables S2, S3 (Supporting Information).

### Cell Culture and Fixation

BS‐C‐1 and HeLa cell lines were purchased from the Korean Cell Line Bank and cultured at 2.5 × 104 cells mL^−1^ density. The cells were cultured in Minimum Essential Medium (MEM) supplemented with 10% fetal bovine serum (FBS), 1% penicillin‐streptomycin, and 1% sodium pyruvate. All cells were incubated at 37 °C in 5% CO_2_. For tubulin staining, cells were washed briefly with 1× phosphate‐buffered saline (PBS) 3 times. Then cells were extracted for 30 s with cytoskeleton extraction buffer^[^ ^71^ ^]^ (0.1 M 1,4‐piperazinediethanesulfonic acid (PIPES), 1 mM ethylene glycol‐bis(2‐aminoethylether)‐N,N,N′,N′‐tetraacetic acid (EGTA), 200 mM sodium hydroxide (NaOH), 1 mM magnesium chloride (MgCl_2_), 0.2% Triton X‐100, pH 7). The extracted cells were fixed with tubulin fixation solution (3% paraformaldehyde (PFA), 0.1% glutaraldehyde (GA) in 1× PBS) for 10 min, followed by a reduction with 0.1% sodium borohydride (NaBH_4_) in 1× PBS for 7 min, and then rinsed with 0.1 M glycine in 1× PBS 3 times for 5 min.

### Cell Patterning and Culture

Stamps were made with polydimethylsiloxane (PDMS) for linear patterning. A 10:1 ratio of base and curing agent mixture was poured on the patterned silicon wafer and cured in the 70 °C oven over 4 h. PDMS stamps were immersed in 2.0 mg mL^−1^ of dopamine hydrochloride solution (w/v in 10 mM Tris‐HCl buffer, pH 8.5) at room temperature for 1 h.^[^ ^72^ ^]^ After 1 h, patterned surfaces of PDMS stamps were dried with nitrogen gas. Polyacrylamide (PA) gel (11kPa) was made with 40% Acrylamide solution and 2% BIS solution. The gel was prepared similarly to the previously described.^[^ ^73^ ^]^ The poly(dopamine) (PDA) coated surface of the PDMS stamp was contacted with a dried PA‐gel surface and incubated overnight (Figure S31, Supporting Information). A PDMS stencil (with holes of 700 µm diameter) was cleaned with 70% ethanol and distilled water for circular patterning. The stencil was attached to a dried PA‐gel surface, and 0.5 mg mL^−1^ Sulfo‐SANPAH solution (w/v in 50 mM HEPES solution) was poured on the stencil. After that, 365 nm UV light was used to activate the Sulfo‐SANPAH solution for 20 min. Next, the gel was washed twice with 50 mM HEPES solution, and 100 µg mL^−1^ Collagen type I solution (w/v in DPBS) was covered on the stencil. The gel was then incubated overnight.

The C2C12 cell line was used for linear patterning. C2C12 cells were cultured in Dulbecco's Modified Eagle's Medium (DMEM) supplemented with 10% FBS and 1% penicillin‐streptomycin. All cells were incubated at 37 °C in 5% CO_2_. Before the culturing step, PDMS stamps were removed from the gel and washed with DPBS twice. C2C12 cells were cultured on the PDA‐patterned PA‐gel with a cell density 2 × 10^4^ cells cm^−2^. After 30 min, the gel was washed with media twice and incubated. The culture media was changed to differentiation media (DMEM supplemented with 2% horse serum and 1% penicillin‐streptomycin) after one day and differentiated for 7 days. BS‐C‐1 cell line was used for circular patterning. Before the culturing step, the gel was washed with DPBS twice. Next, 200 µL of BS‐C‐1 cell solution (2.5 × 10^4^ cells mL^−1^) was poured on the stencil. After 30 min, the gel was washed with media twice and incubated.

### Staining of Cells

All of the following steps were performed at RT. For permeabilization and blocking, cells were incubated in a blocking buffer (5% normal donkey serum or goat serum, 0.2% Triton X‐100 in 1× PBS) for 1 h. The cells were stained with a primary antibody for 1 h and washed 3 times for 5 min with the blocking buffer. After the primary antibody staining, cells were stained with a secondary antibody for 1 h and washed with the blocking buffer, a PBST buffer (0.2% Triton X‐100 in 1× PBS), and 1× PBS for 5 min each. For iterative staining, MAXblock Blocking Medium was used for permeabilization and blocking, and the antibodies were diluted in MAXbind Staining Medium, followed by the washing step with MAXwash Washing Medium.

### Meat Samples Fixation, Cryo‐Sectioning, and Staining

Purchased meat samples from Jeongyookgak that were as fresh as possible. First, meat samples were trimmed to remove extra fats and fasciae, followed by iced 1× PBS incubation for 1 h at 4 °C. After PBS incubation, meat samples were trimmed again for residue removal and the proper size for cryo‐sectioning, followed by iced 1× PBS incubation for 1 h at 4 °C once again. Trimmed meat samples were transferred into 4% PFA fixative solution at 4 °C on a rocker, protected from the light, overnight. After PFA fixation, 1× PBS incubation was performed overnight at 4 °C. The fixed samples were incubated in 10, 20, and 30 w/w% sucrose solution for 1 day each at 4 °C without shaking. Using an OCT mold, the meat samples were then incubated into the OCT compound above the dry ice. The samples were kept in a deep freezer until cryo‐sectioning. Cryo‐sectioning was conducted in KAIST BioCore Center with Cryocut Microtome (CM3050S, Leica). The thickness of the cryo‐sectioned sample was 20 or 30 µm. The meat slices were collected onto silane‐coated glass and kept in the deep freezer before the experiments. Before the staining of the meat slices, the slices were dehydrated in 37 °C convection oven for 30 min. After dehydration, a rectangular well was attached to the glass to make a chamber. A humid chamber was also used to protect the samples from dehydration and light during subsequent steps. The samples were washed with 1× PBS for 5 min 3 times, followed by PBST incubation for 30 min, and then washed again with 1× PBS for 5 min 3 times. For the staining of meat slices, blocking, staining, and washing steps were identical to the cultured cell protocol except for the incubation time (blocking: 1.5 h, staining: overnight, washing: 30 min).

### Meat Collagenase Treatment

For meat collagenase treatment, collagenase type IV powder was used. Collagenase powder was diluted at 0.1 U µL^−1^ in 3 mM CaCl_2_, 1×HBSS solution. The washed meat samples were incubated in the collagenase solution at 37 °C for 2 h. After incubation, the samples were carefully washed with 1× PBS for 5 min thrice.

### Protein‐Retention Expansion Microscopy (Pro‐ExM)

For protein‐retention expansion microscopy, the stained cells in removable chambered cover glasses were incubated in acryloyl‐X, SE ((6‐((acryloyl)amino) hexanoic acid, succinimidyl ester, AcX) diluted to 0.1 mg mL^−1^ in 1× PBS for 1 h at RT and then washed 3 times for 5 min with 1× PBS. The samples were then incubated twice with a monomer solution (7.5% (w/w) sodium acrylate, 2.5% (w/w) acrylamide, 0.15% (w/w) N,N′‐methylenebisacrylamide (BIS), 1× PBS, 2 M NaCl) at 4 °C for 15 min each time. After incubation, the samples were filled with a gelation buffer (monomer solution, 0.2% (w/w) ammonium persulfate (APS), 0.2% (w/w) tetramethylethylenediamine (TEMED), 0.01% (w/w) 4‐hydroxy‐2,2,6,6‐tetramethylpiperidin‐1‐oxyl (H‐TEMPO)) and incubated at 37 °C for 1.5 h. After gelation, the chambers of the culture wells were removed, and the gels on the cover glasses were treated with proteinase K diluted at 1:100 in a digestion buffer (25 mM ethylendiaminetetraacetic acid (EDTA), 50 mM Tris‐HCl (pH 8), 0.5% Triton X‐100, 1 M NaCl) at RT overnight with gentle shaking. After digestion, to identify the nucleus, the gels were washed with PBST buffer for 30 min and then incubated 1 µg mL^−1^ DAPI solution in PBST buffer for 1 h followed by washing with PBST buffer for 30 min. After DAPI staining, for identifying boundary of cells, the gels were incubated with Atto 565 dye solution (10 mg mL^−1^ ATTO were diluted with 0.1 M NaHCO_3_ as 1:700 volumetric ratio) for 4 h at RT, and then gels were washed with 1× PBS for 30 min 3 times, followed by washing with deionized water (DW) multiple times with gentle shaking until the size of the gels plateaued.

### In Situ Material Growth from Labeled Biostructures

The antibodies labeled samples were post‐fixed with 1% GA in 1× PBS for 10 min and then washed with DW 3 times for 5 min each. For silver nanoparticle (AgNP) growth from the post‐fixed samples, samples were washed with 0.02 M sodium citrate 3 times for 5 min each. Two AgNP growth methods were used in this work. The first was using a commercial silver growth kit (HQ Silver) according to the manufacturer's protocol, followed by thoroughly washing with DW for 1 min 3 times. Another one was a modified protocol from a related paper.^[^ ^74^ ^]^ The silver growth solution comprised 0.5 g mL^−1^ gum arabic, 200 mM HEPES, 0.5 M hydroquinone, and 50 mM silver lactate, a 6: 2: 1: 1 volumetric ratio. The hydroquinone and silver lactate solution should be made just before use, and the hydroquinone solution should be added at the end. The post‐fixed samples were incubated in the silver growth solution for 30 min at 25 °C while shielded from light. After silver growth, the samples were thoroughly washed with an SCT buffer (0.02 M sodium citrate, 0.2% Triton X‐100 in DW) once, 0.02 M sodium citrate 2 times, and DW wash 3 times for 5 min each. For gold nanoparticle (AuNP) growth from the post‐fixed samples, a commercial gold growth kit (GoldEnhance EM) was used according to the manufacturer's protocol, followed by thoroughly washing with DW for 1 min 3 times.

### Biocompatibility Test of Cell Substrates

For the cell viability assay, the fixed, silver‐, and gold‐grown cell substrates were dehydrated and kept in a vacuum desiccator prior to use. BS‐C‐1 cells were seeded as described in the cell culture section and incubated for 7 days. Per the manufacturer‘s protocol, the biocompatibility test was conducted using the Live/Dead Cell Imaging Kit. The cells were incubated with an equal volume of 2× working solution for 15 min at room temperature.

### Imaging; Optical and Electron Microscopy

Two microscopy systems were used in this work. The first one was a spinning‐disk confocal microscopy system, which was Andor Dragonfly (Oxford Instruments) through an inverted microscope, Ti2‐E (Nikon), or upright microscope, BX51WI (Olympus). The other was a scanning confocal microscopy system, C2 plus (Nikon) through Ti2‐E. A brightfield imaging filter mode on Fusion software obtained brightfield images.

For scanning electron microscopy (SEM) imaging, the samples were coated with an osmium plasma coater, HPC‐1SW (Vacuum Device). SEM imaging was performed on a scanning electron microscope, SU82300 (Hitachi) of KARA (KAIST Analysis Center for Research Advancement). Regions of interest in samples were imaged using a BSE detector, with accelerating voltages of 10 kV for SEM.

For analysis using scanning transmission electron microscopy (STEM) imaging, material‐grown samples were dehydrated through a graded ethanol series of 30%, 40%, 50%, 60%, 70%, 80%, 90%, 95%, and 100% for 15 min each, followed by a transition in 99.5% propylene oxide (EMS) for 20 min. Transient cells by propylene oxide were incubated in propylene oxide‐resin solutions mixed in 2 : 1, 1 : 1, and 1 : 2 ratios for 1 h each, and then the cells were incubated in 100% resin overnight at room temperature, followed by polymerization of resin at 40 °C in a convection oven for 48 h. The resin blocks were trimmed and sectioned at 300 nm thickness with an ultramicrotome (EM UC7, Leica), and the sections were collected on copper grids. The ultramicrotome sectioning was done in the EM & Histology Core Facility at the BioMedical Research Center, Korea Advanced Institute of Science and Technology. STEM images were acquired in KARA (KAIST Analysis Center for Research Advancement) with a transmission electron microscope (Titan cubed G2 or Talos F200X) at 300 kV with high angle annular dark‐field (HAADF) detector and dispersive X‐ray spectroscopy (EDS). Images were processed for better visibility through ImageJ software and ImarisViewer software. Detailed information about the imaging is listed in Table S4 (Supporting Information).

### UV–Vis Spectrophotometer

The absorbance spectra were recorded using UV–Vis spectrophotometer, UV‐1800 (SHIMADZU) through UV‐Probe program (interval: 0.5, medium scanning mode) or UV–Vis 1900i (SHIMADZU) (interval: 1.0, slow scanning mode).

### Raman Spectroscopy

RS experiments were performed with a dispersive Raman spectroscopy ARAMIS (HORIBA) and LabRAM HR Evolution Visible_NIR (HORIBA). Regarding ARAMIS, 2 excitation laser wavelengths were used: 515 nm (Cobolt Fandango) and 633 nm (Melles Griot). The laser intensity measured at the sample was set from 20 µW to 1.8 mW according to lasers and objectives. The laser was focused onto the sample with a 50× (numerical aperture, N.A. = 0.5) or 100× objective (numerical aperture, N.A. = 0.9). In the case of LabRAM HR Evolution, 515 nm (Cobolt Fandango, Sweden) was used as an excitation laser wavelength. The laser intensity measured at the sample was set to 0.1 mW with a 100× objective (numerical aperture, N.A. = 0.9). The scattered Raman signal was collected with the same objective and detected by a CCD detector (ARAMIS: Synapse / LabRAM HR Evolution: HORIBA) and cooled down to ‐70 °C. UEye UI‐146xLE Series (ARAMIS, IDS) or UI‐1460LE‐C‐HQ obtained bright field images during RS measurements (LabRAM HR Series (ARAM, IDS). To evaluate SERS performance through rhodamine 6G (R6G), R6G was dissolved in ethanol to prepare a solution with 10^−3^ M concentrations, followed by dilution with DW to 10^−10^ M solution. For bovine serum albumin (BSA), BSA powder was dissolved in DW to prepare a solution with a concentration of 50 mg mL^−1^. R6G and BSA solutions were drop‐cast onto substrates and fully dried before SERS measurements. Commercial SERS substrates were purchased from PiCO Foundry Inc.

### Electromagnetic Simulation of Plasmonic Structures

Silver nanoparticles with a radius of 10 nm were arranged in a helical structure similar to the labeled microtubules with antibodies. Multiple rounds of these labeled structures were simulated beyond the first round. The nanoparticles were positioned in all designs to maintain proximity between neighboring particles. An electromagnetic simulation was conducted to determine the absolute value of the electric field under light injection. The finite‐difference time‐domain (FDTD) method was adopted using Ansys Lumerical FDTD Solutions (Ansys Inc.). A plane wave source of λ  =  515 nm was injected along the z‐axis, which was assumed. During the simulation, perfect‐matched layer (PML) boundaries were applied along the x‐ and z‐axes, while a periodic boundary was used along the y‐axis corresponding to the helix's axis. The period along the y‐axis was set to 20 nm, and the mesh size was determined to be 0.25 nm. The permittivity of the silver nanoparticles was (−9.324+0.795j)^[^ ^75^ ^]^ in the simulation. With an electric field amplitude of 1 for the incident plane wave sources, the log(|E|) was plotted on the x‐y plane. This plot determines the maximum log(|E|) value along the z‐axis for fixed x‐ and y‐coordinates. Only the upper half of the helix along the z‐axis was used. The electric field data for the 2 polarizations (along the helix axis and the helix radius) were calculated separately.

### Inductively coupled plasma‐reactive ion etching (ICP‐RIE) process, aptamer functionalization and complementary DNA, and BPA incubation

The metalized cells and meat samples were etched by inductively coupled plasma‐reactive ion etching (ICP‐RIE) using argon plasma (50–60 W, 30 sccm, 10–15 mTorr). BPA‐specific aptamer and its complementary ssDNA used in this study were synthesized by Integrated DNA Technologies, IDT, with previously reported sequences described in Table S5, Supporting Information. Before aptamer functionalization, gold‐grown cell substrates were incubated in pre‐DNA treatment buffer (0.2 mg mL^−1^ Salmon sperm DNA, 0.1% Triton X‐100 in 1× PBS) for 3 h at 4 °C. Thiol‐modified aptamer was first dissolved in pre‐DNA treatment buffer with aptamer concentrations being 6 µM, followed by mixing with 600 µM TCEP (1:1 by volume) for 2 h at room temperature for thiol reduction. The resulting solution (final aptamer solution concentration = 3 µM) was pipetted onto substrates, which were subsequently incubated for 3 h under wet conditions at 4 °C. After aptamer solution incubation, substrates were thoroughly washed with pre‐DNA treatment buffer 3 times.

For complementary DNA binding to BPA target aptamer, complementary DNA was diluted in pre‐DNA treatment buffer with a concentration of 3 µM. Aptamer functionalized substrates were incubated in the complementary DNA solution for 4 h at room temperature in the dark, followed by washing with pre‐DNA treatment buffer once, and A buffer (0.1 M NaCl, 0.025 M KCl, 0.01 M MgCl_2_, 0.02 M Tris‐HCl in 5% DMSO, pH 8.0 adjusted) twice thoroughly to remove non‐specifically bound DNAs.

For BPA incubation, BPA was first dissolved in DMSO as a stock solution with a concentration of 20 mM, and then BPA solutions were diluted with A buffer, and then double‐strand DNA functionalized substrates were incubated in BPA solutions for 80 min at 37 °C. After BPA incubation, substrates were washed with A buffer 2 times thoroughly.

### Time of Flight Secondary Ion Mass Spectrometer (ToF‐SIMS)

ToF‐SIMS was performed on TOF‐SIMS 5 (IONTOF) of KARA (KAIST Analysis Center for Research Advancement). Bi^+^ ion was used as a primary ion with 30 keV, a positive mode. ToF‐SIMS images were obtained through an imaging mode (1 FOV: 200 × 200 µm, Raster size (pixels): 256 × 256). The elemental peaks were assigned via spectrometry mode. All data were processed using the analysis software SurfaceLab7.

### X‐ray Photoelectron Spectroscopy (XPS)

XPS was performed on KARA's K‐alpha (Thermo VG Scientific) with a monochromatic Al‐Kα X‐ray source. The obtained XPS spectra were fitted in casaXPS software.

### 2D, 3D Design Illustration

2D illustrations were generated through BioRender. 3D illustrations of microtubules and antibodies were generated through Autodesk 3ds Max software.

## Conflict of Interest

J.‐B.C, D.‐H.S., and C.W.S declare competing interests. J.‐B.C, D.‐H.S., and C.W.S are co‐inventors on multiple patents and patent applications owned by KAIST concerning the material growth technique used in this manuscript.

## Supporting information



Supporting Information

Supplementary Video

Supplementary Video

Supplementary Video

## Data Availability

The data that support the findings of this study are available from the corresponding author upon reasonable request.
