# Supplementary material for: Highly Tunable, Nanomaterial‐Functionalized Structural Templating of Intracellular Protein Structures Within Biological Species
Source: Adv Sci (Weinh). 2024 Nov 13;12(2):2406492. doi: 10.1002/advs.202406492 (PMC11727137; doi:10.1002/advs.202406492)
Supplement: Supplementary file 1 — Supporting Information [file ADVS-12-2406492-s004.docx]

Supporting Information

**Highly tunable, nanomaterial-functionalized structural templating of intracellular protein structures within biological species**

*Dae-Hyeon Song, Chang Woo Song, Seunghee H. Cho, Tae Yoon Kwon, Hoeyun Jung, Ki Hyun Park, Jiyun Kim, Junyoung Seo, Jaeyoung Yoo, Minjoon Kim, Gyu Rac Lee, Jisung Hwang, Hyuck Mo Lee, Jonghwa Shin, Jennifer H. Shin, Yeon Sik Jung^*^, Jae-Byum Chang^*^*

Supplementary Movies

**Movie S1.**

Z-stack confocal image of beta-tubulin/DAPI stained BS-C-1 cells after expansion *via* pro-ExM shown in Fig. 1A. The last frame is a 2D MIP image.

**Movie S2.**

Z-stack confocal image of beta-tubulin stained C2C12 cells without pattern.

**Movie S3.**

Z-stack confocal image of beta-tubulin stained C2C12 cells with pattern. The thickness of line patterns is 100 microns.


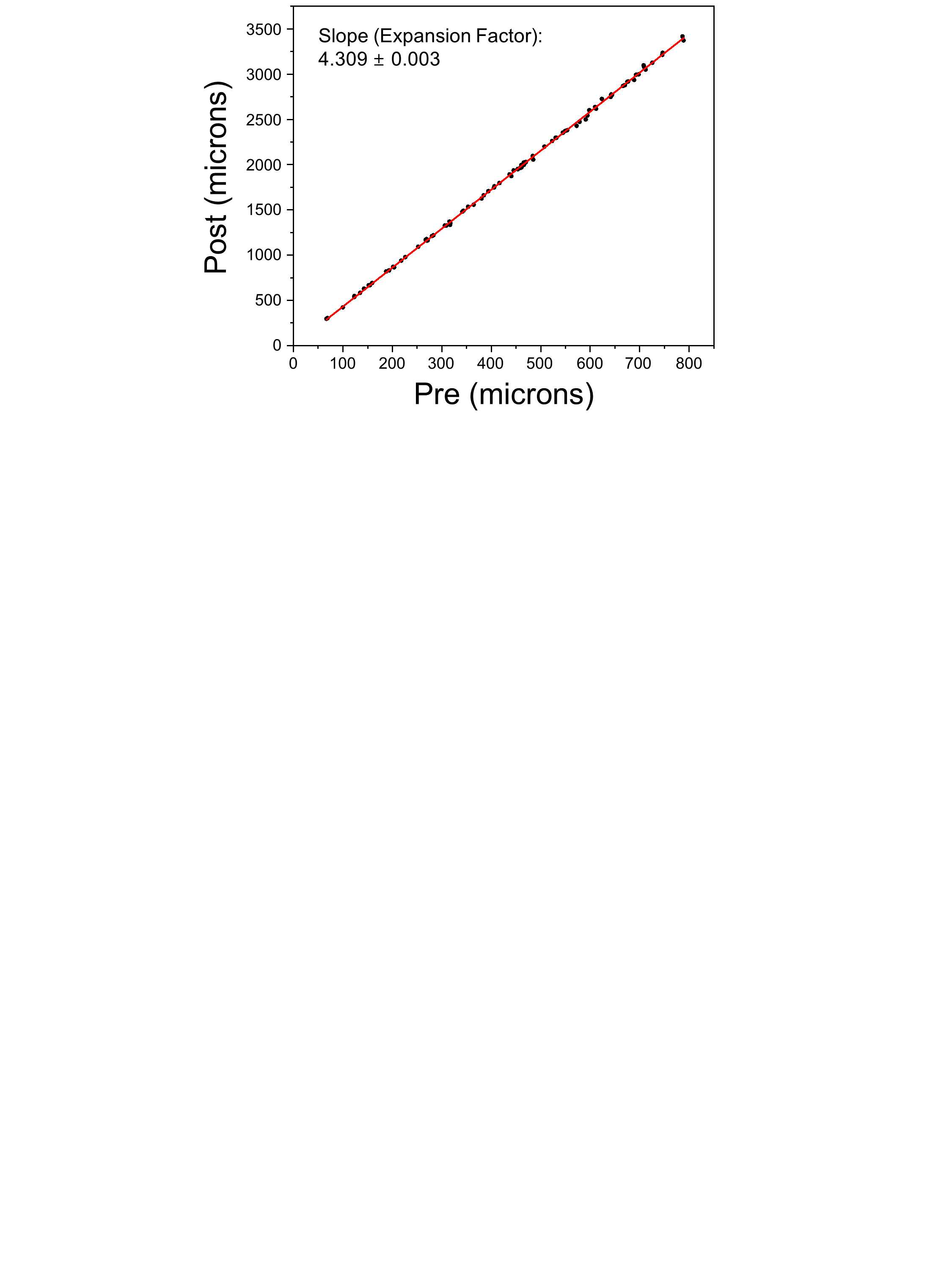


**Fig**. **S1**. **Expansion factor calculation through a comparison between the pre-and post-expansion states**. The expansion factor was determined by measuring the distance between two landmarks in the specimen before versus after the expansion. *n* = 90 points, 30 lines each, were measured from three independent wells.

**
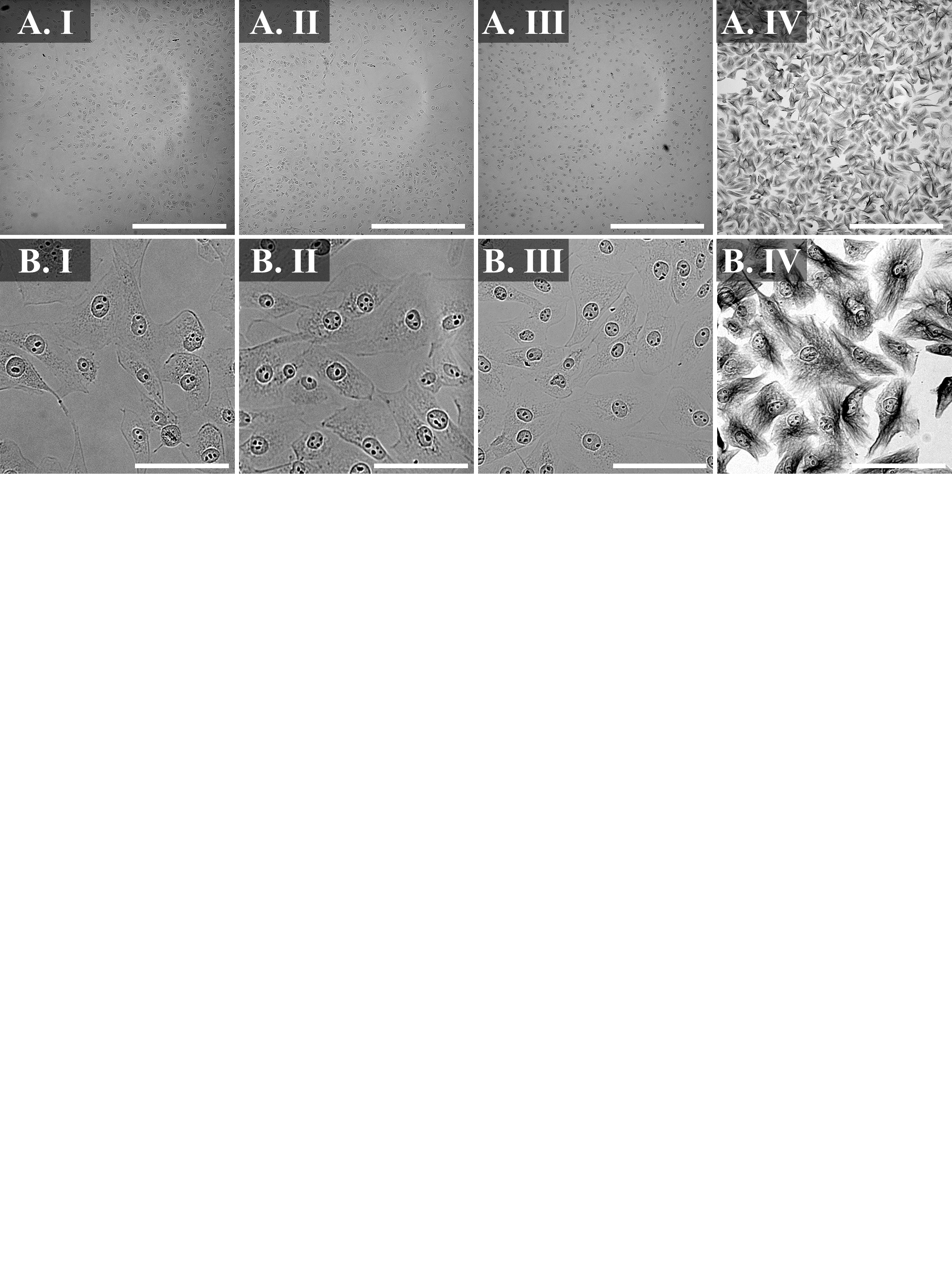
**

**Fig**. **S2**. **Bright-field (BF) images according to steps in Fig. 1B**. (**I**) Fixed cell state. (**II**) Primary Ab stained state. (**III**) NG-conjugated secondary Ab stained state. (**IV**) Silver-grown state (**A**: Low-magnified / **B**: High-magnified images). Scale bar. A. 500 μm, B. 100 μm.


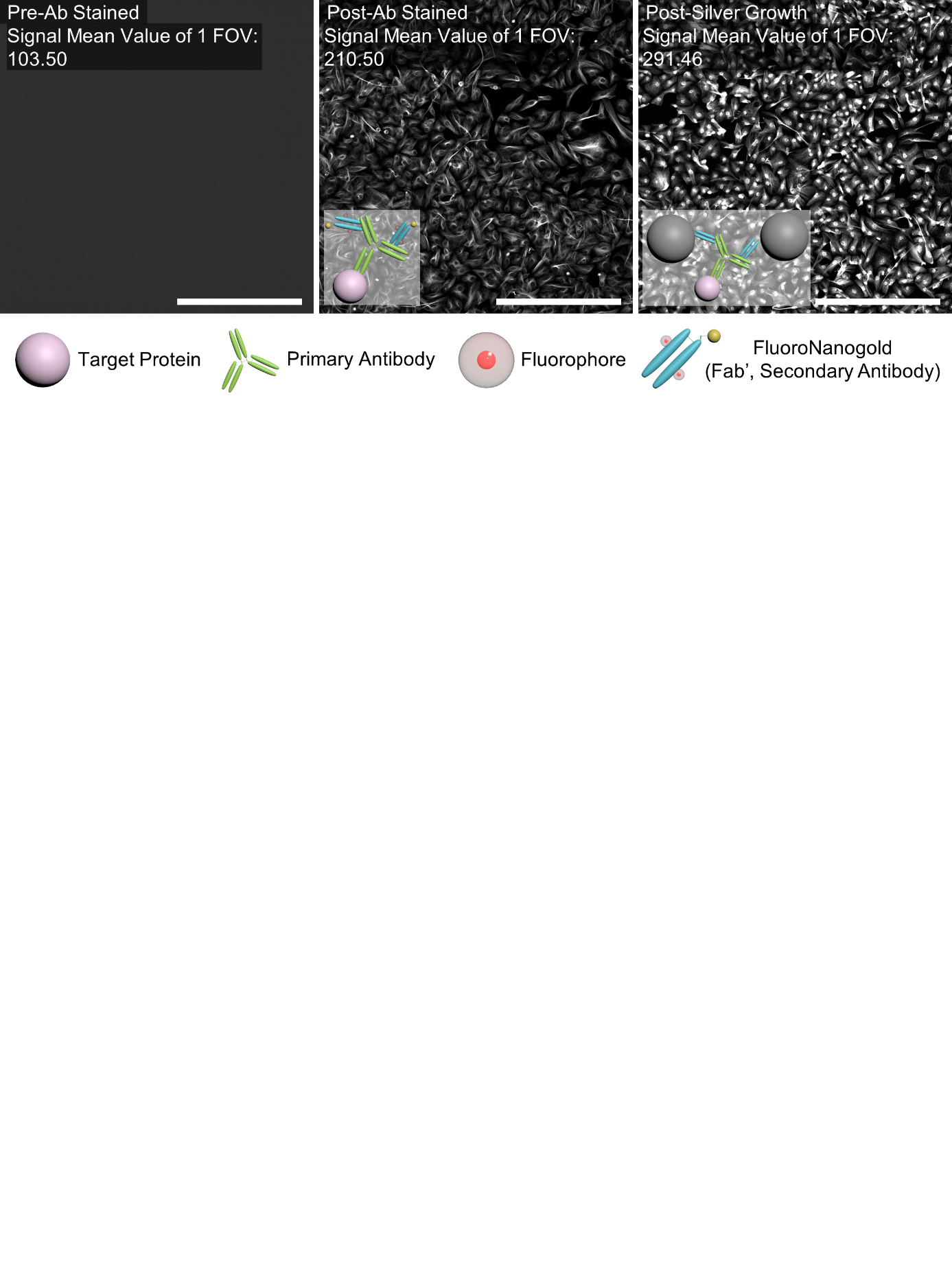


**Fig**. **S3**. **Fluorescence microscopy (FM) images according to FluoroNanogold (FNG) antibody-staining and silver-growth**. Mean signal values (a.u.) for each field of view (FOV) are written at the top of each image, and simple schematics are at the bottom left. Scale bar. 500 μm.


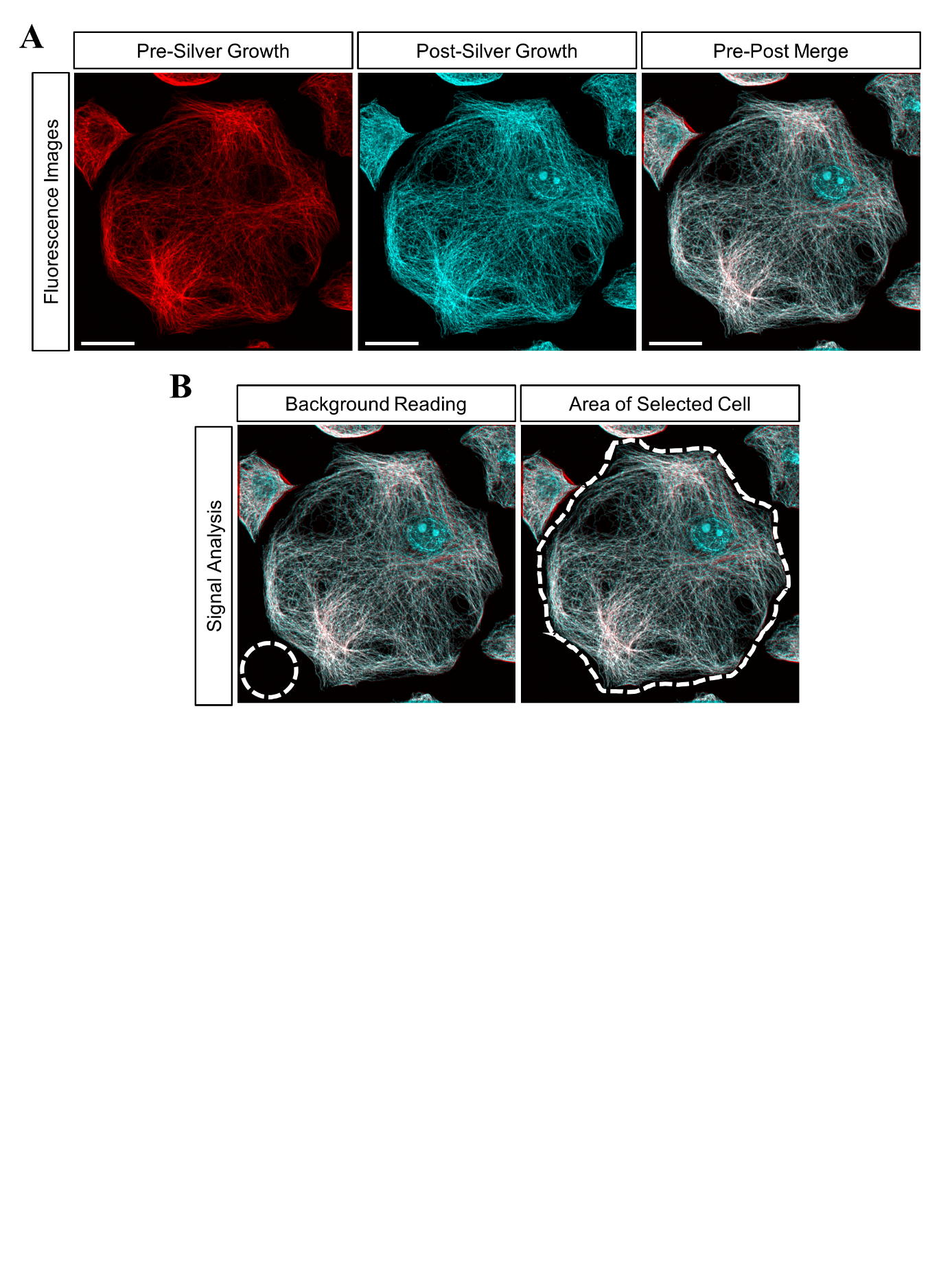


**Fig**. **S4**. **Strategy for comparison of fluorescence signals between pre- and post-silver growth state.** (**A**) FM images of beta-tubulin antibody stained cells pre- and post-silver growth (red: pre- / cyan: post-silver growth). (**B**) Specific regions selected for calculating fluorescence signals in the merged pre- and post-images (left: background signal / right: antibody stained region). Maximum-intensity projection (MIP) was performed for each image. Fluorescence signals were obtained by subtracting the mean signal value of 'background reading' from the 'area of selected cell.' Scale bar. 30 μm.


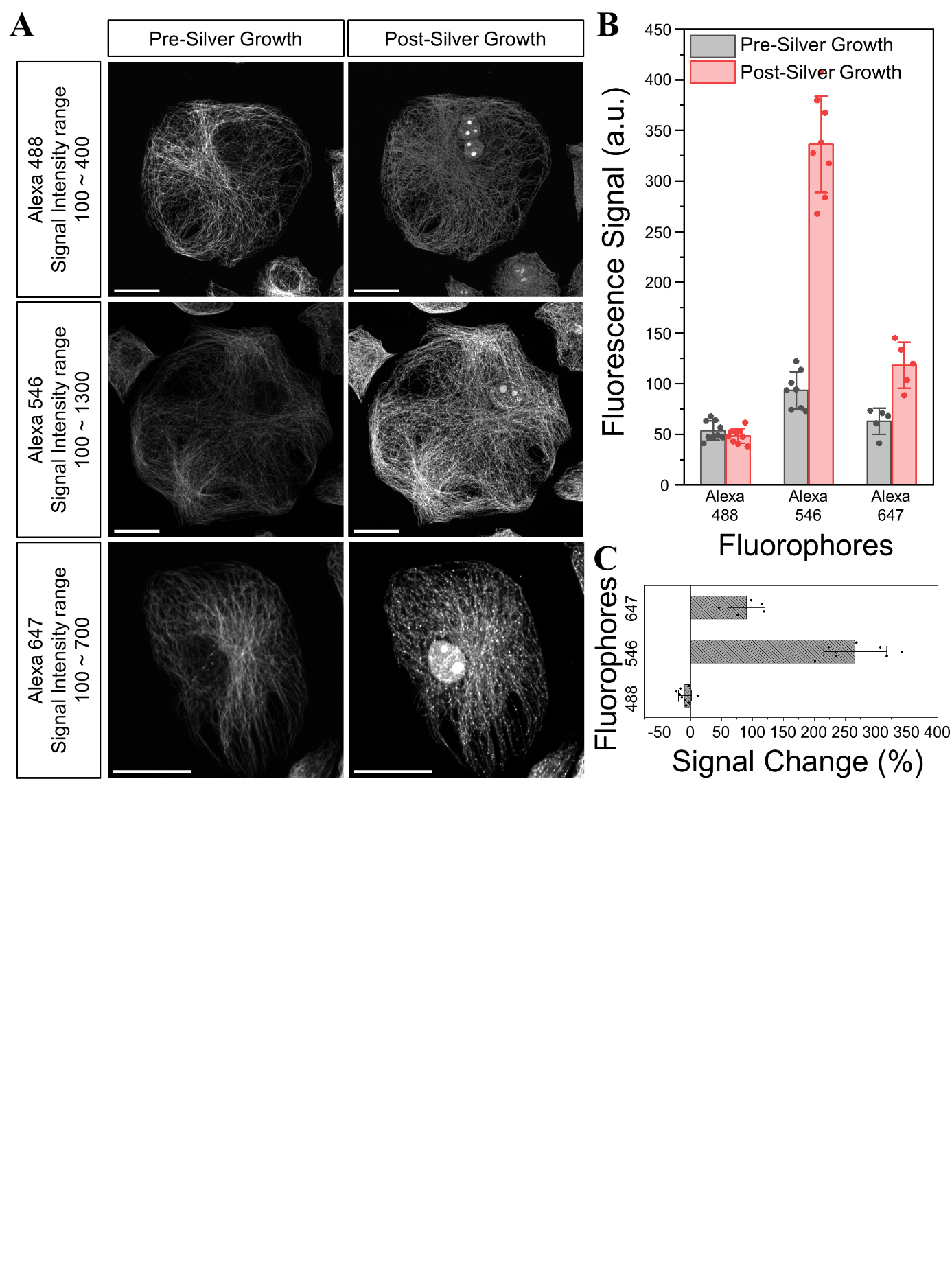


**Fig**. **S5**. **Fluorescence signal comparison between pre- and post-silver grown state *via* different fluorophore-conjugated FNG antibodies**. (**A**) Representative FM images of cells stained with different FNG antibodies pre- and post-silver growth. MIP was performed for all images. (**B**) Fluorescence signals within all cases. (Data are presented as mean ± s.d., Alexa 488: *n* = 9, Alexa 546: *n* = 8, Alexa 647: *n* = 5 from five independent cells in a single substrate). (**C**) Signal changes between pre- and post-silver growth according to fluorophores. In the case of Alexa 488 dye, the slight decrease in signal is due to the overlap between the absorption wavelengths of AgNPs and the emission wavelengths of the dye. Scale bar. 30 μm.


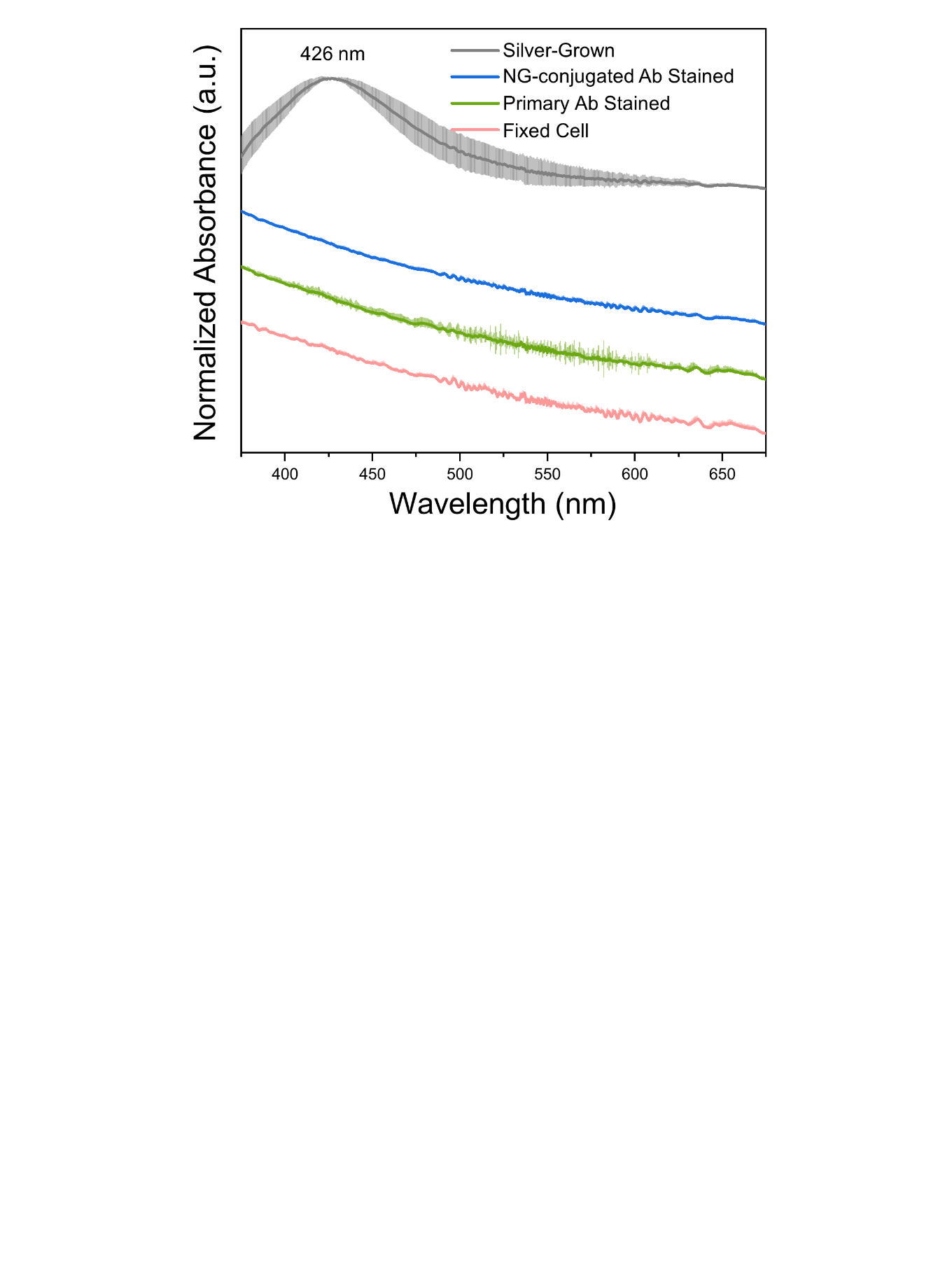


**Fig**. **S6**. **UV-vis spectra corresponding to each step of the CamBio process**. Each data was normalized separately for better visibility. Data are presented as mean ± s.d., *n* = 3 from three independent substrates.


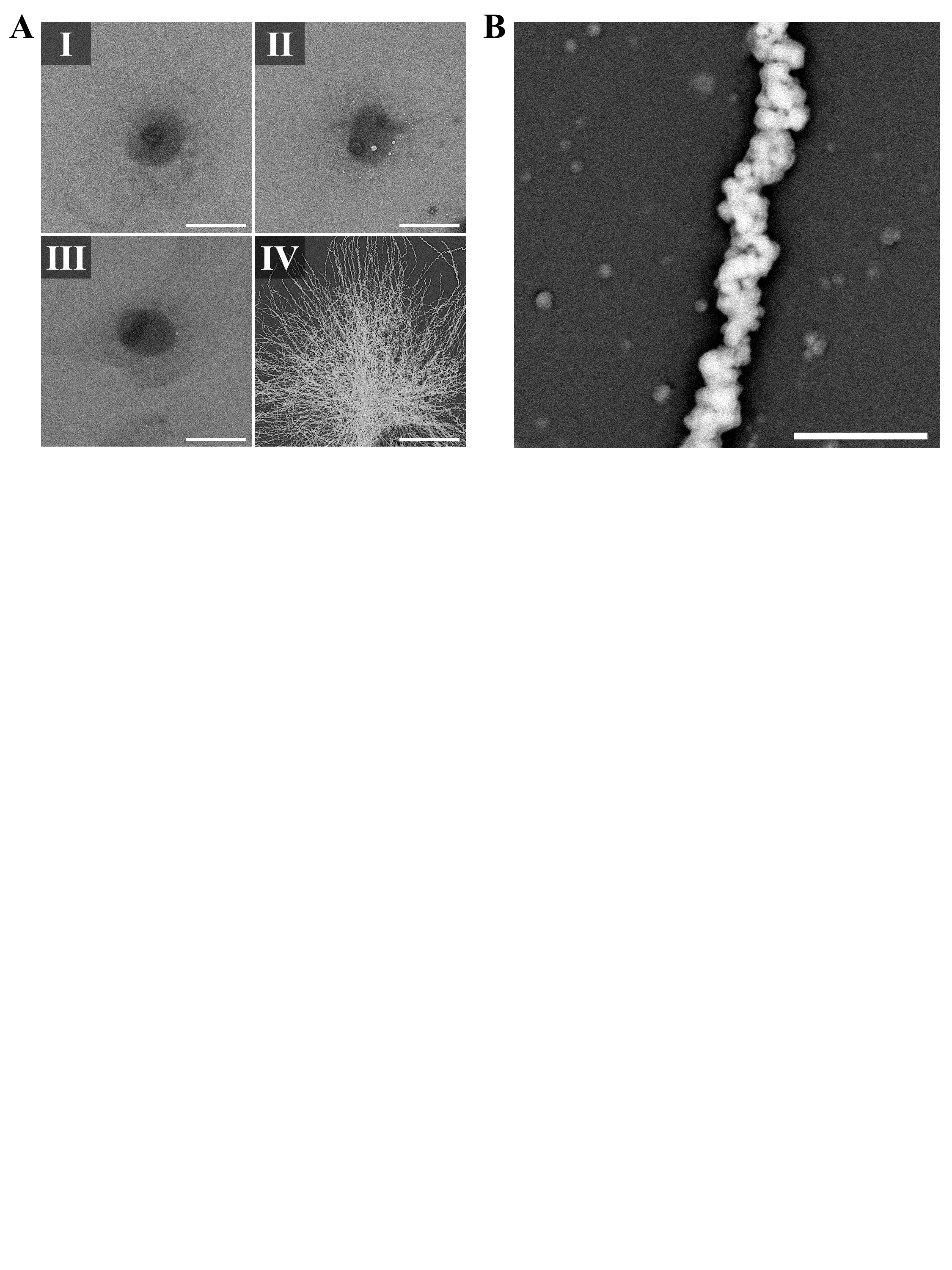


**Fig**. **S7**. **Highly magnified SEM images corresponding to each step of the CamBio**. (**A**) SEM images of single cellular level magnification corresponding to each step of the CamBio process. (**I**) Fixed cell state (target protein structure: microtubule). (**II**) Primary Ab stained state. (**III**) NG-conjugated secondary Ab stained state. (**IV**) Metal-grown state (silver, Ag). (**B**) Magnified SEM image of single silver nanoparticle (AgNP) chain at silver-grown state. Scale bar. A. 20 μm, B. 500 nm.

**
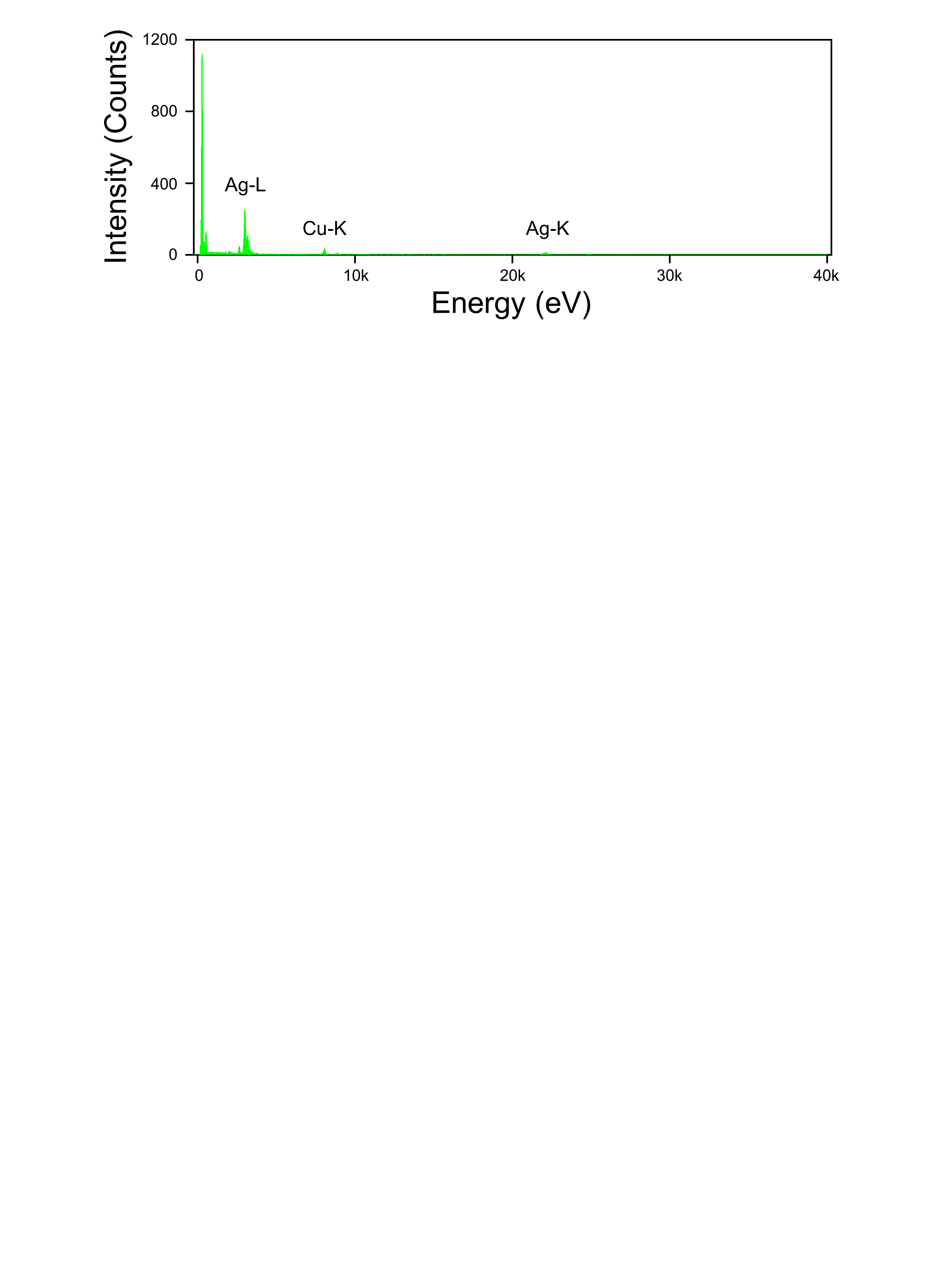
**

**Fig**. **S8**. **EDS spectrum obtained from the same region shown in Fig. 1D**.


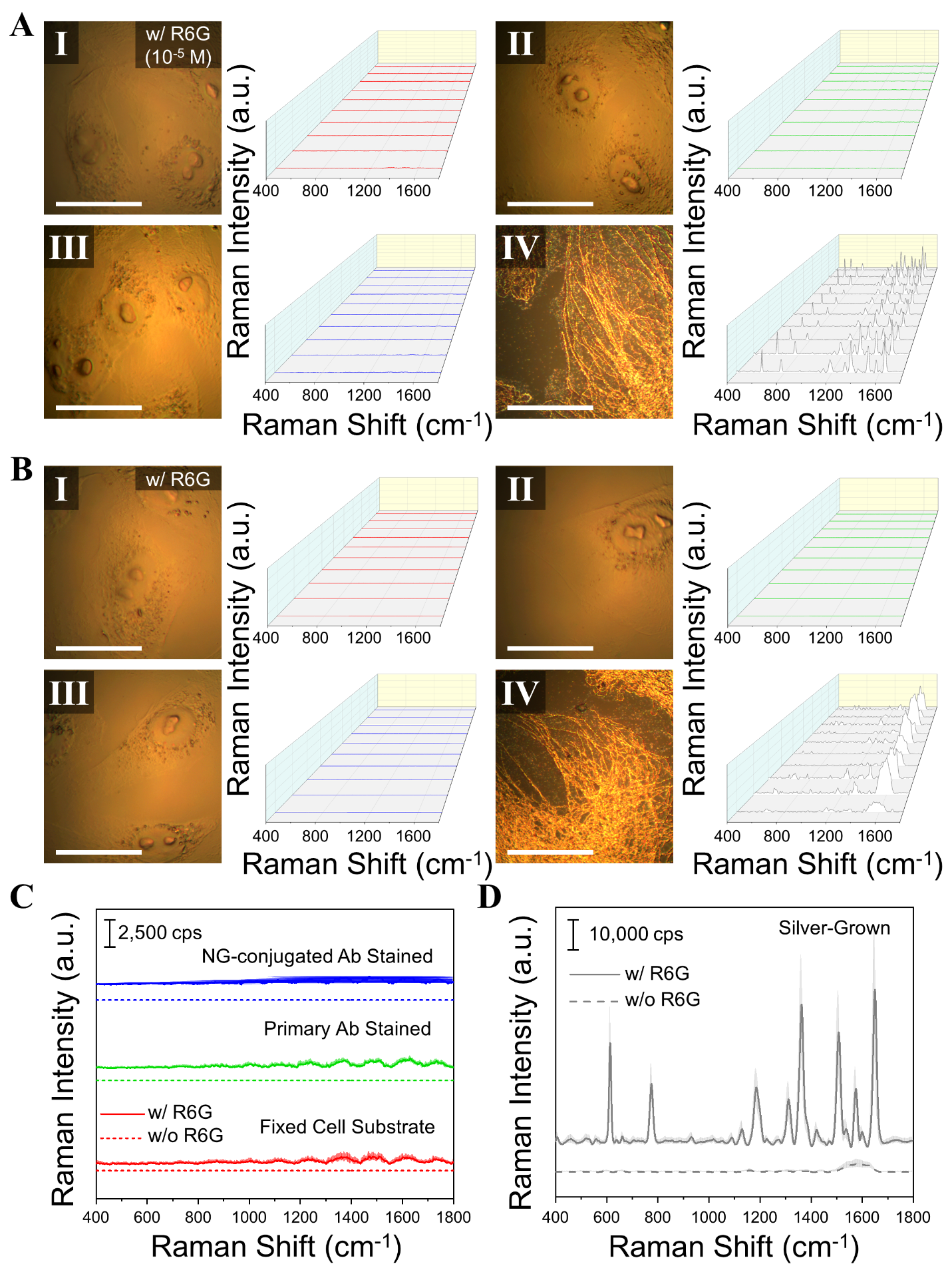


**Fig**. **S9**. **Reproducible SERS spectra of w/ and w/o R6G recorded on cell substrates of each step**. SERS spectra of (A) w/ R6G (10,000 cps per unit) and (B) w/o R6G (10,000 cps per unit) were recorded on cell substrates corresponding to each step in Fig. 1B with representative bright-field images acquired during measurement (*n* = 10 points from ten independent cells in a single substrate). Average SERS spectra (± standard deviation) from (C) pre-silver growth states and (D) post-silver grown state (solid line: w/ R6G, dash line: w/o R6G). Scale bar. 30 μm.


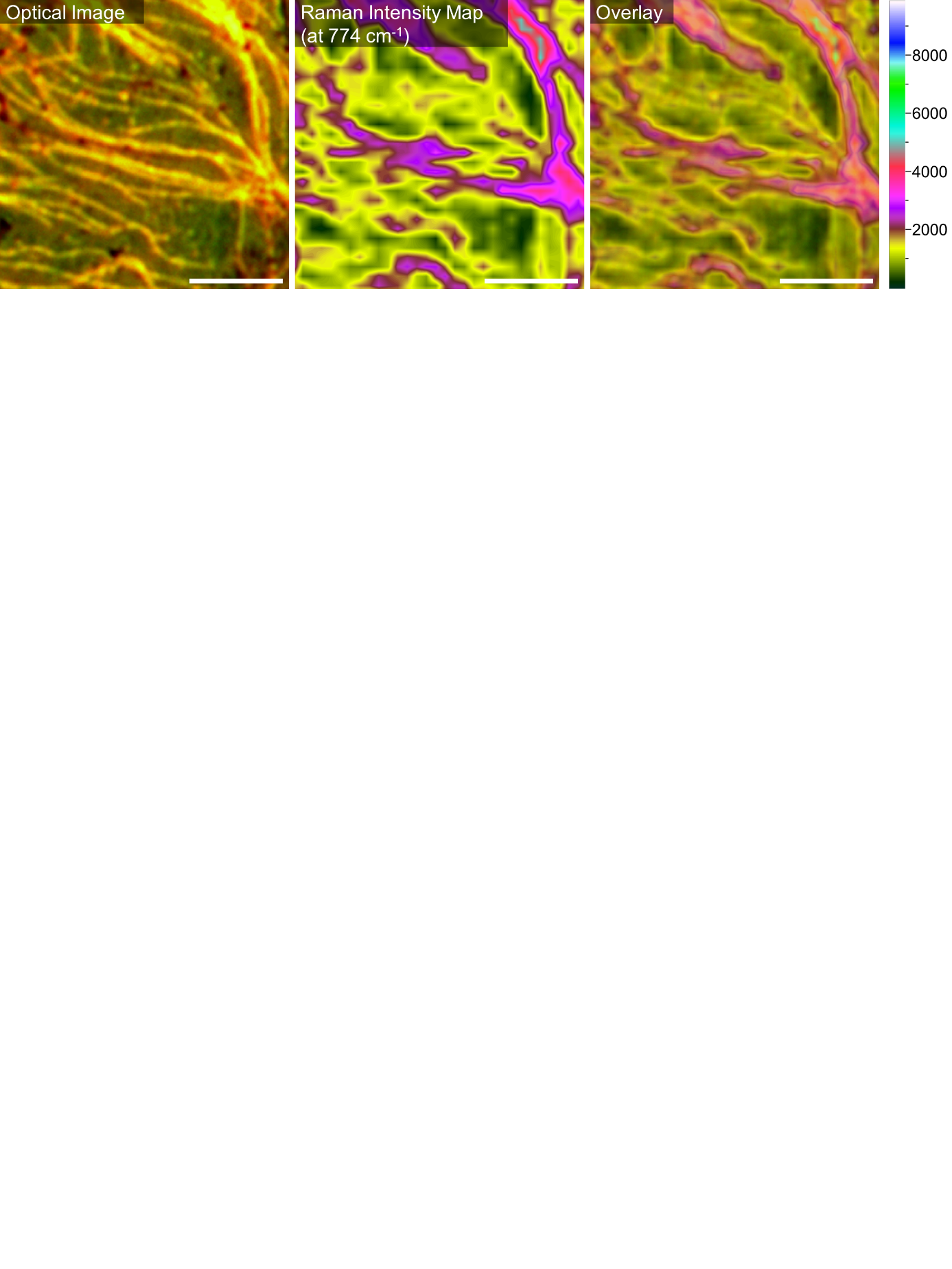


**Fig**. **S10**. **Mapping of Raman signal intensities from R6G molecules along with AgNP chains converted from microtubule structures in cells.** Scale bar. 5 μm.


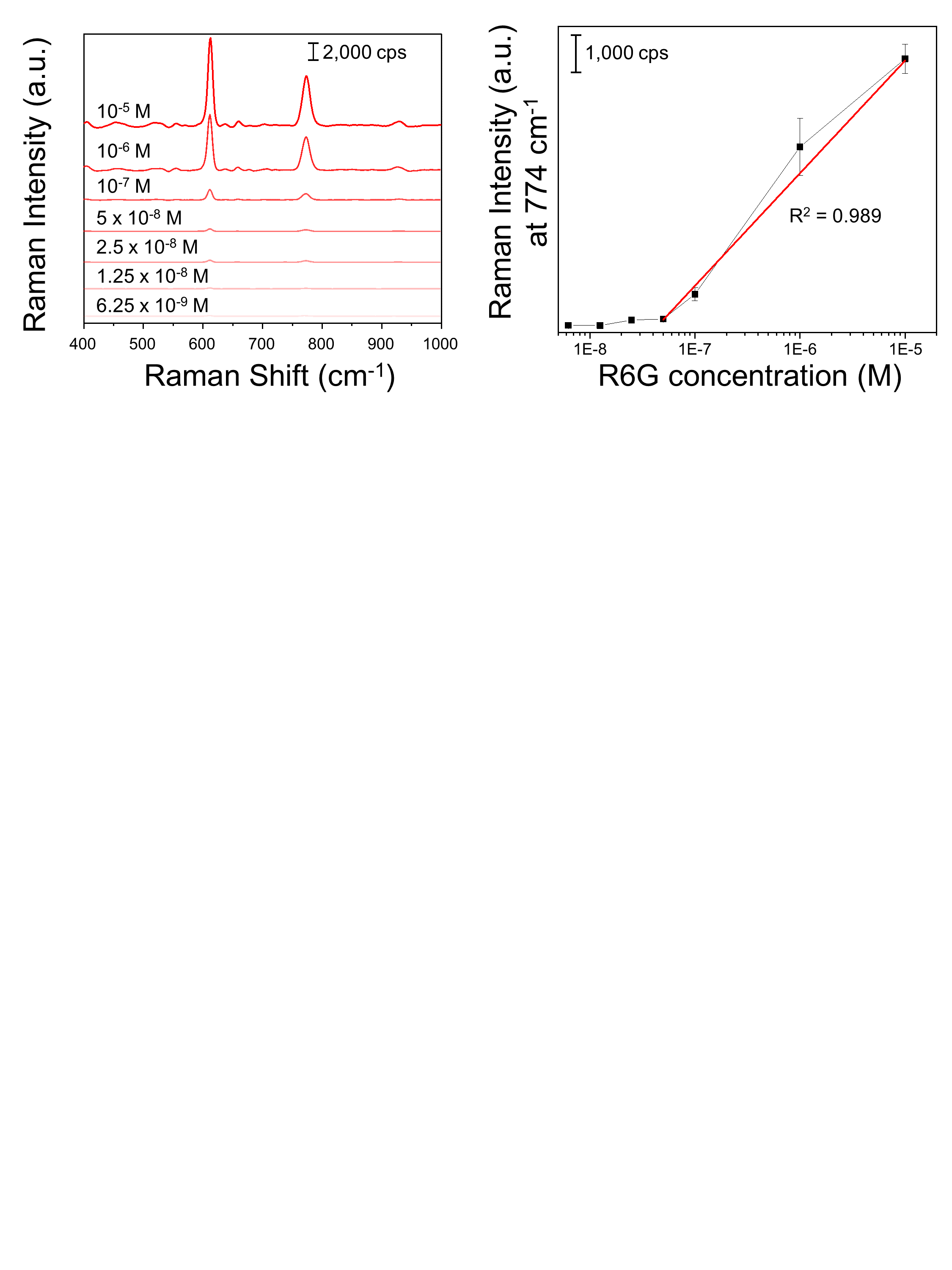


**Fig**. **S11**. **Raman spectra of R6G by concentration obtained from the silver-grown substrate, where AgNP chains were converted from microtubule structure through the CamBio process and standard curve using Raman intensity of a characteristic peak from R6G molecule.** The detection limit (DL) is the minimum concentration where linearity is maintained for the standard curve (DL: 5 × 10^-8^ M). For the enhancement factor (EF) calculation, the Raman signals of R6G (102 M) from the NG conjugated Ab state substrates were used as a reference. EF is derived using the following equation.

EF = (I_SERS_ × C_Ref_) / (I_Ref_ × C_SERS_)

I_SERS_, I_Ref_, C_SERS_, and C_Ref_ each represent the intensity of the SERS signal on the silver-grown substrate, the intensity of the Raman signal on the NG conjugated Ab substrate, the concentration of R6G solution on the silver-grown substrate, and the concentration of R6G solution on the NG conjugated Ab substrate. Data are presented as mean values from *n* = 5 points of five independent cells, each concentration in a single substrate.


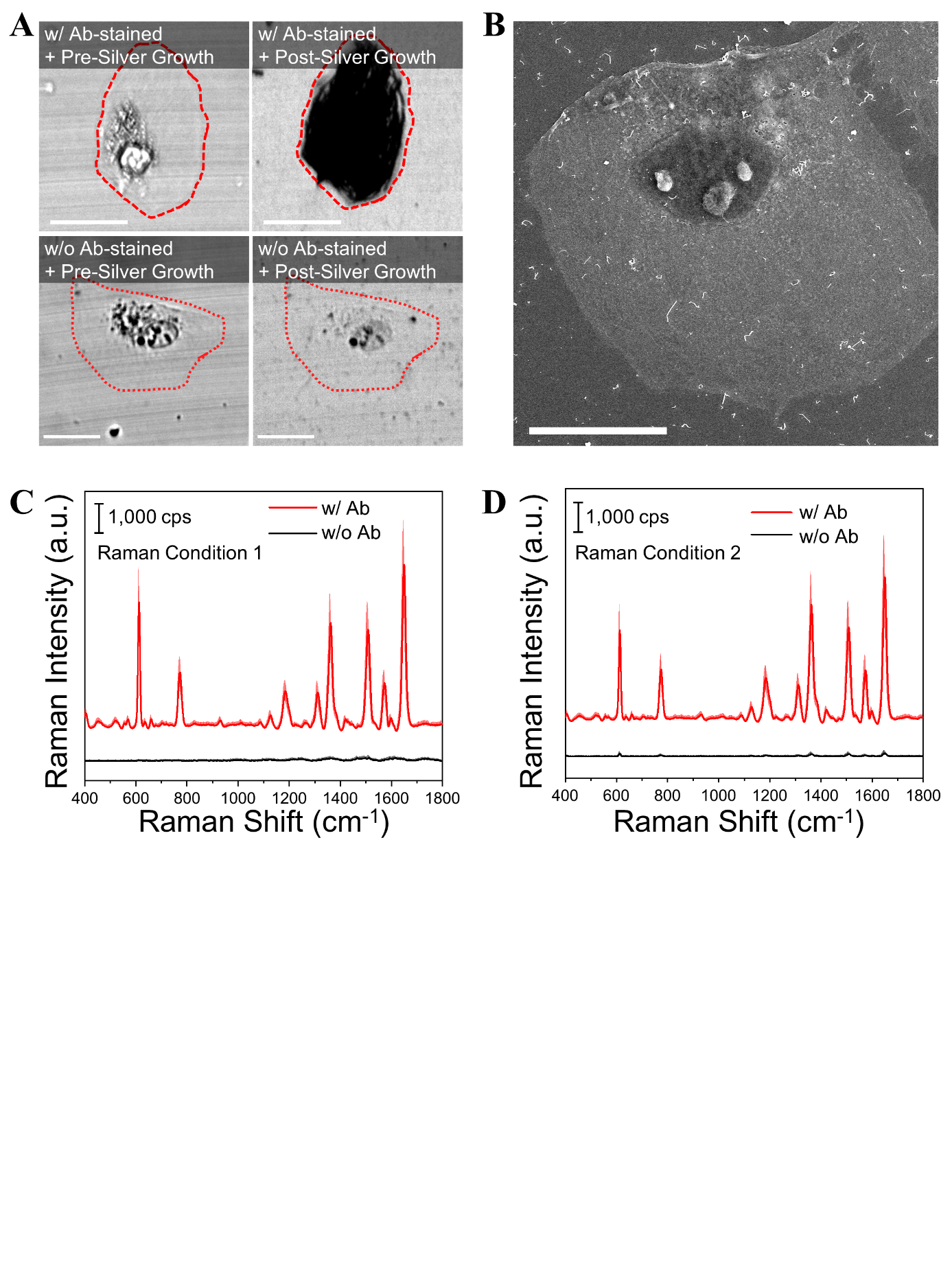


**Fig**. **S12**. **CamBio process results depending on antibody staining.** (**A**) BF images from pre- and post-silver growth cells with and without antibody staining. (**B**) SEM image of the silver-grown cell without antibody staining. (**C, D**) SERS spectra of R6G depending on antibody staining. The spectra were obtained at two different Raman conditions. Data are presented as mean ± s.d. with (**C**) total *n* = 7 points in each case from two independent wells, (**D**) *n* = 10 points from ten independent cells in a single substrate. Scale bar. A. 30 μm, B. 20 μm.


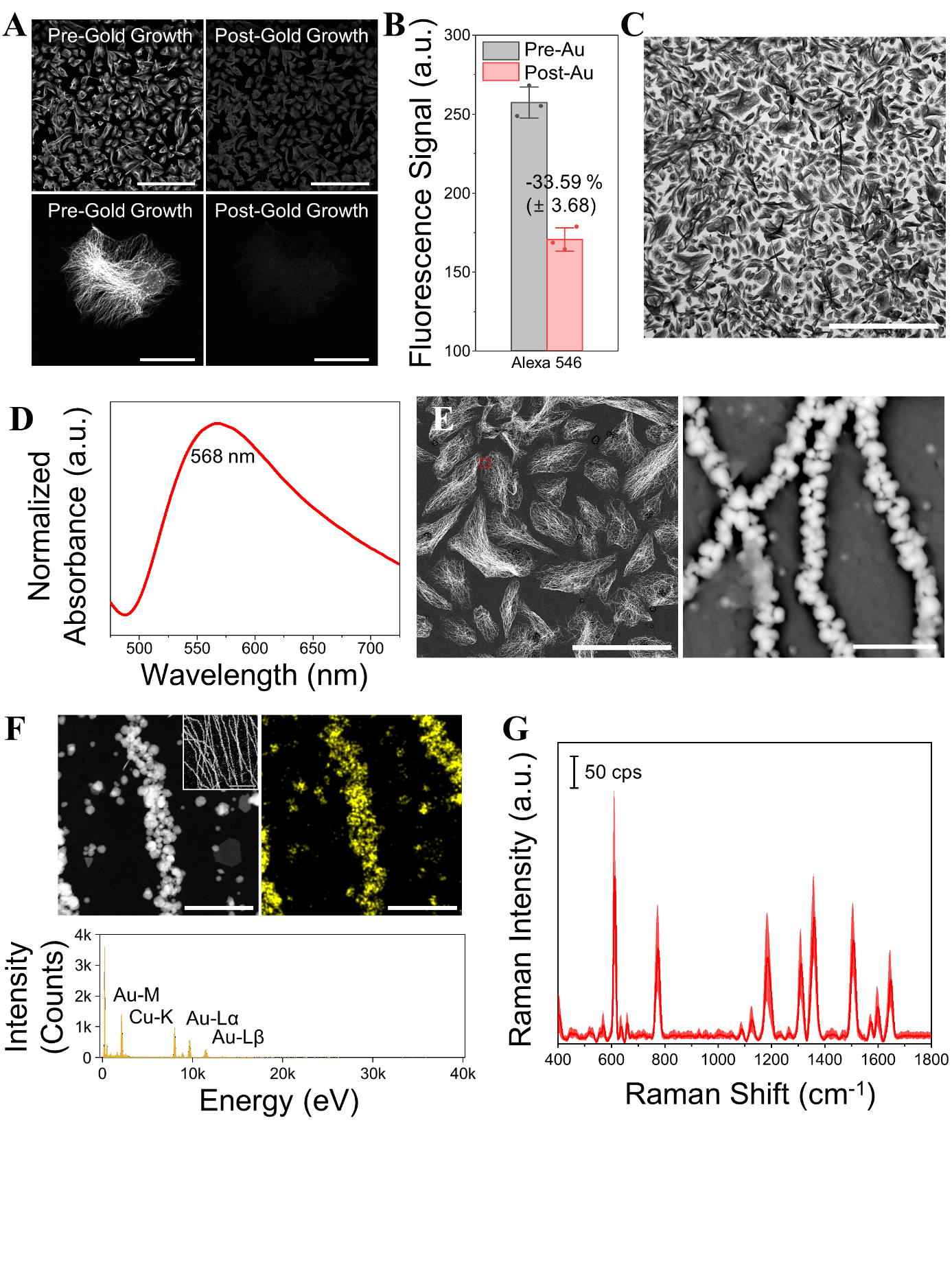


**Fig**. **S13**. **CamBio results from a target protein structure with gold growth.** (**A**) FM images of FNG stained cell substrate and a representative single cell depending on gold growth. (Signal range of images; top (tile): 100 – 1500 (a.u.) / bottom (single cell): 100 – 1000 (a.u.). (**B**) Signal changes between pre- and post-gold growth. Data for each step are presented as mean ± s.d., *n* = 3 points from three independent cells in a single substrate. (**C**) BF image, (**D**) UV-vis spectrum (*n* = 1), and (**E**) SEM images of gold-grown cell substrate. SEM images are magnified images inside the red boxes on the left. (**F**) STEM (left), EDS mapping (right) image, and EDS spectrum of gold nanoparticle (AuNP) chains converted from microtubule structure *via* CamBio (Inset image: a low-magnified image of STEM, Scale bar: 2μm). (**G**) Average SERS spectrum (± s.d.) of R6G from the gold-grown state, *n* = 7 points from seven independent cells in a single substrate. Scale bar. A. 300 μm (top), 50 μm (bottom), C. 500 μm, E. 300 μm (left), 500 nm (right), F. 300 nm.


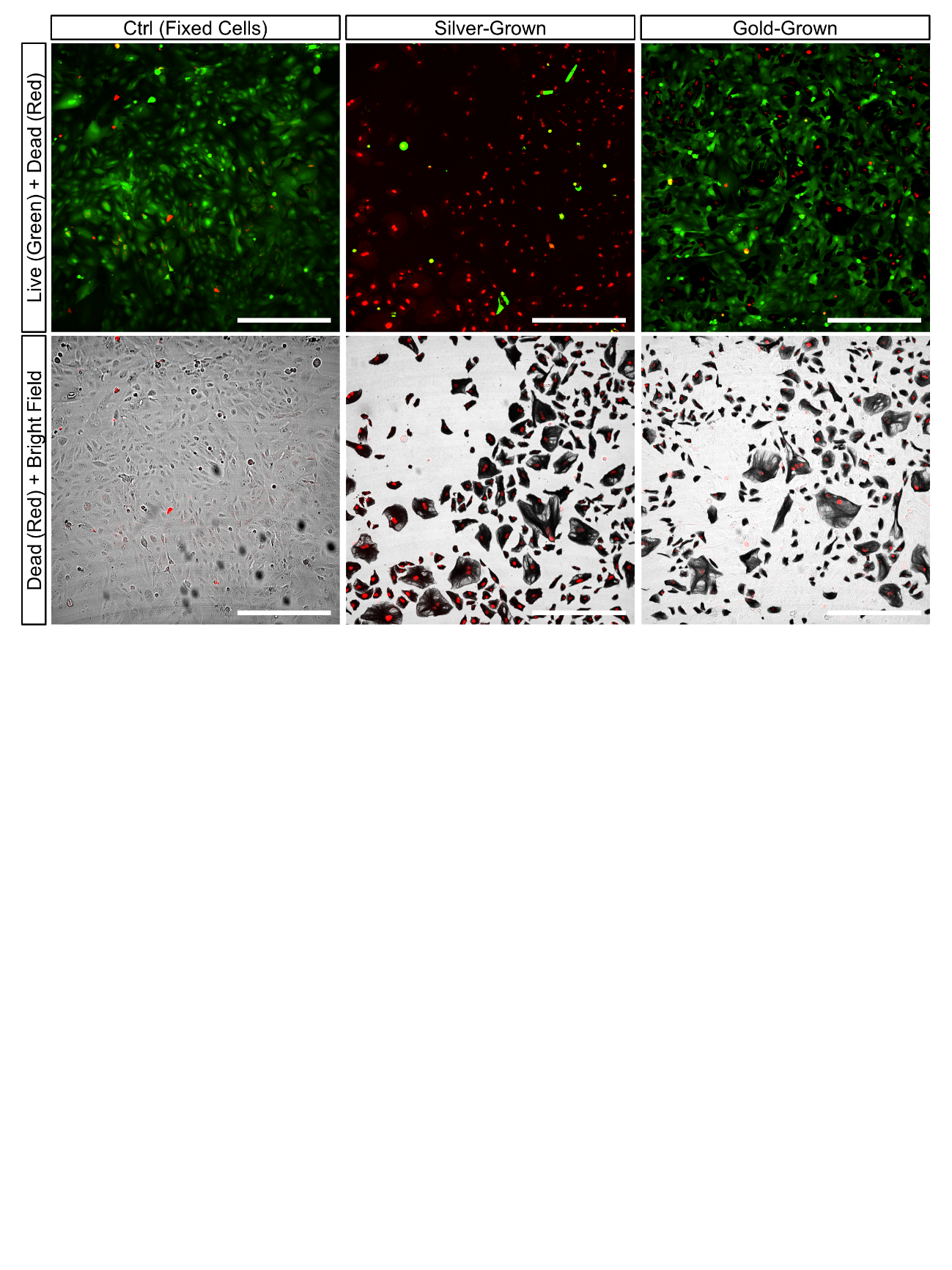


**Fig. S14. Biocompatibility of cell substrates (target molecule: α-tubulin; green: live; red: dead).** When merged with BF images, most dead signals were confirmed to have been generated from the fixed or converted cells on the substrates. Silver-grown substrates appeared to negatively impact cell proliferation. Scale bar. 400 μm.

**
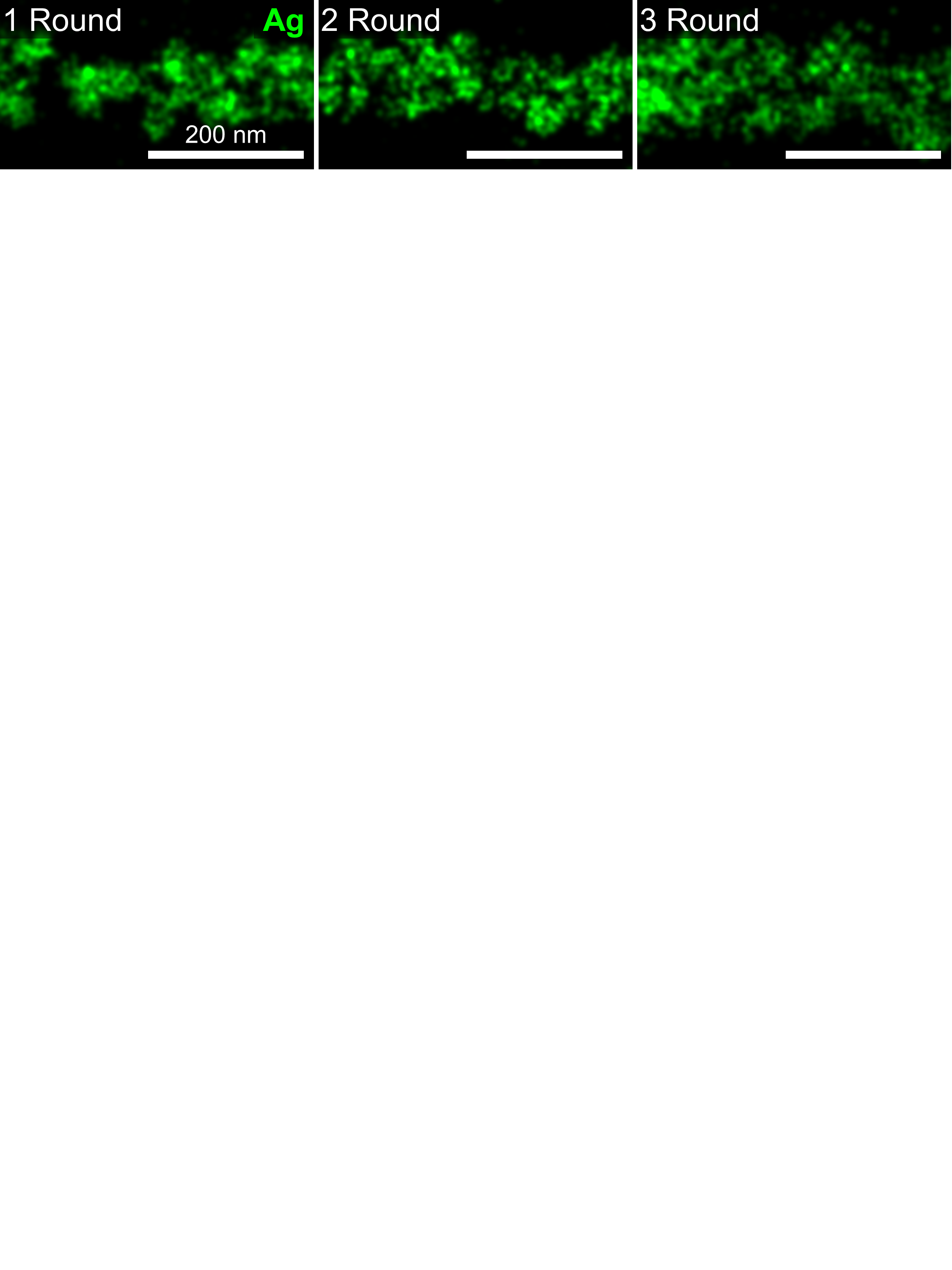
**

**Fig**. **S15**. **EDS mapping images (Ag) of AgNP chains converted from microtubule structure from each round of iterative staining.** Scale bar. 200 nm.

**
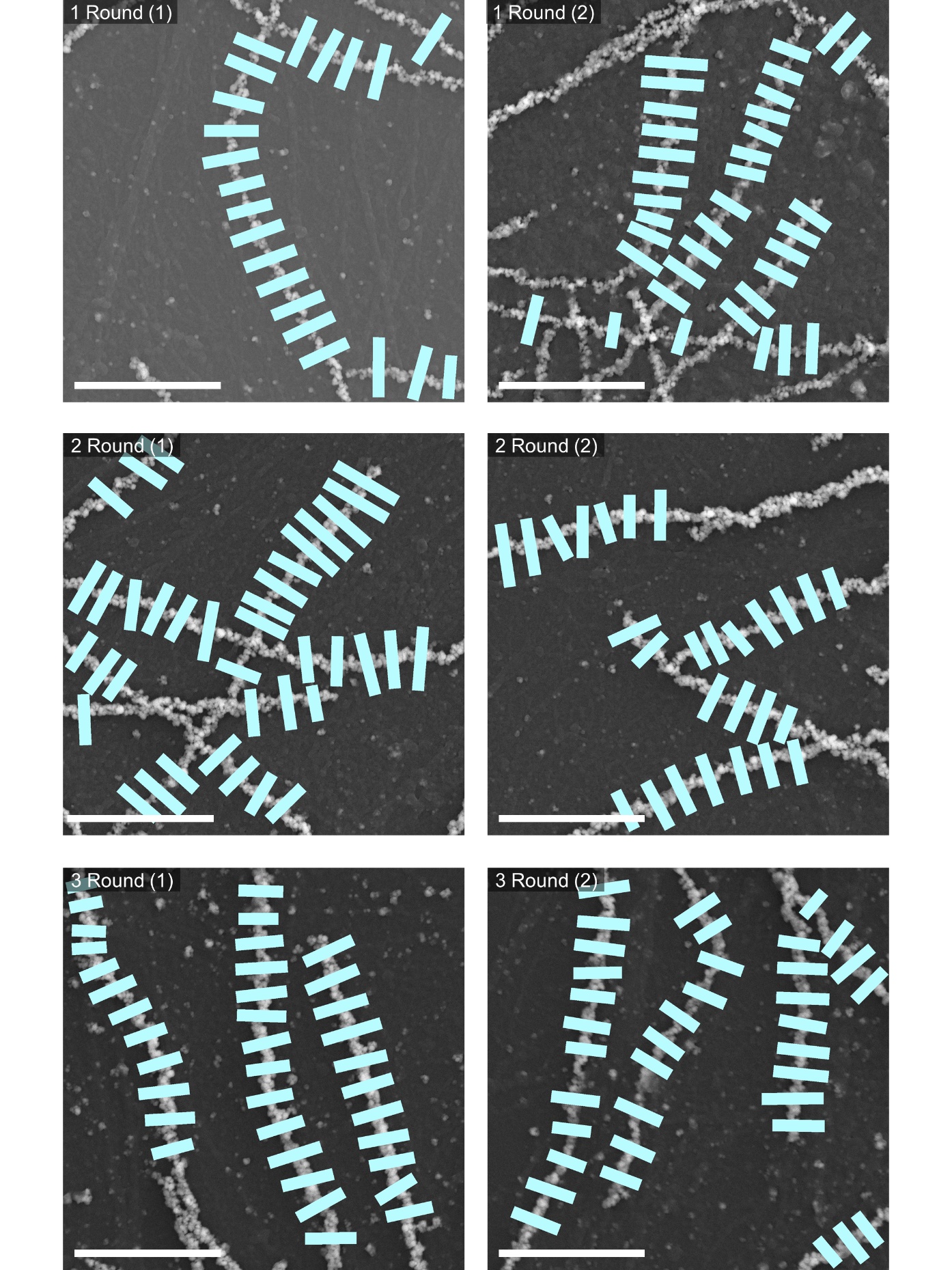
**

**Fig**. **S16**. **Low-magnification SEM images of AgNP chains in cells at each round.** Blue lines represent regions used for SEM profile analysis. Scale bar. 2 μm.


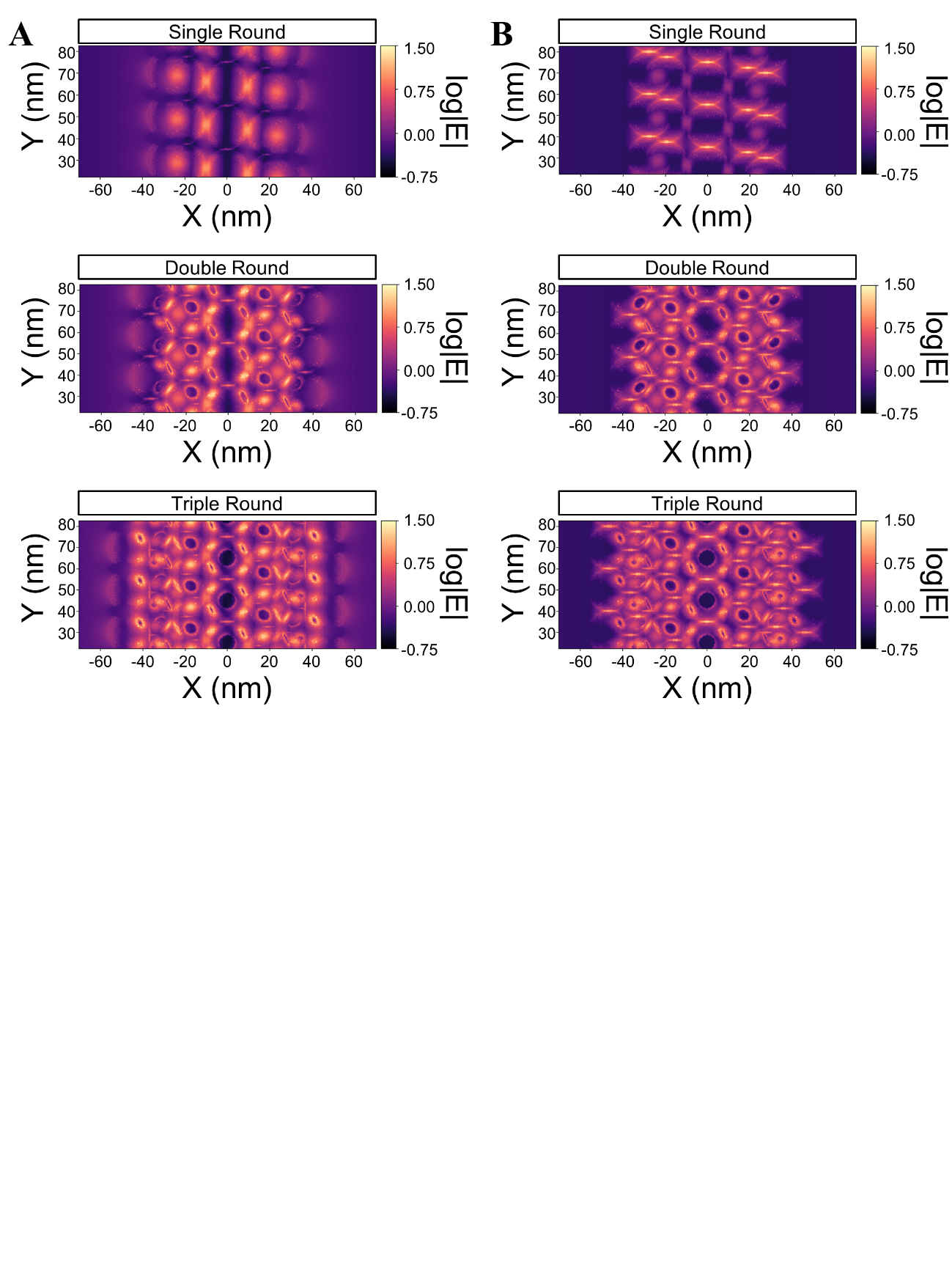


**Fig**. **S17**. **Electromagnetic simulations of iteratively labeled microtubules with silver growth.** (**A**) Data for polarization along the helix radius. (B) Data for polarization along the helix axis.


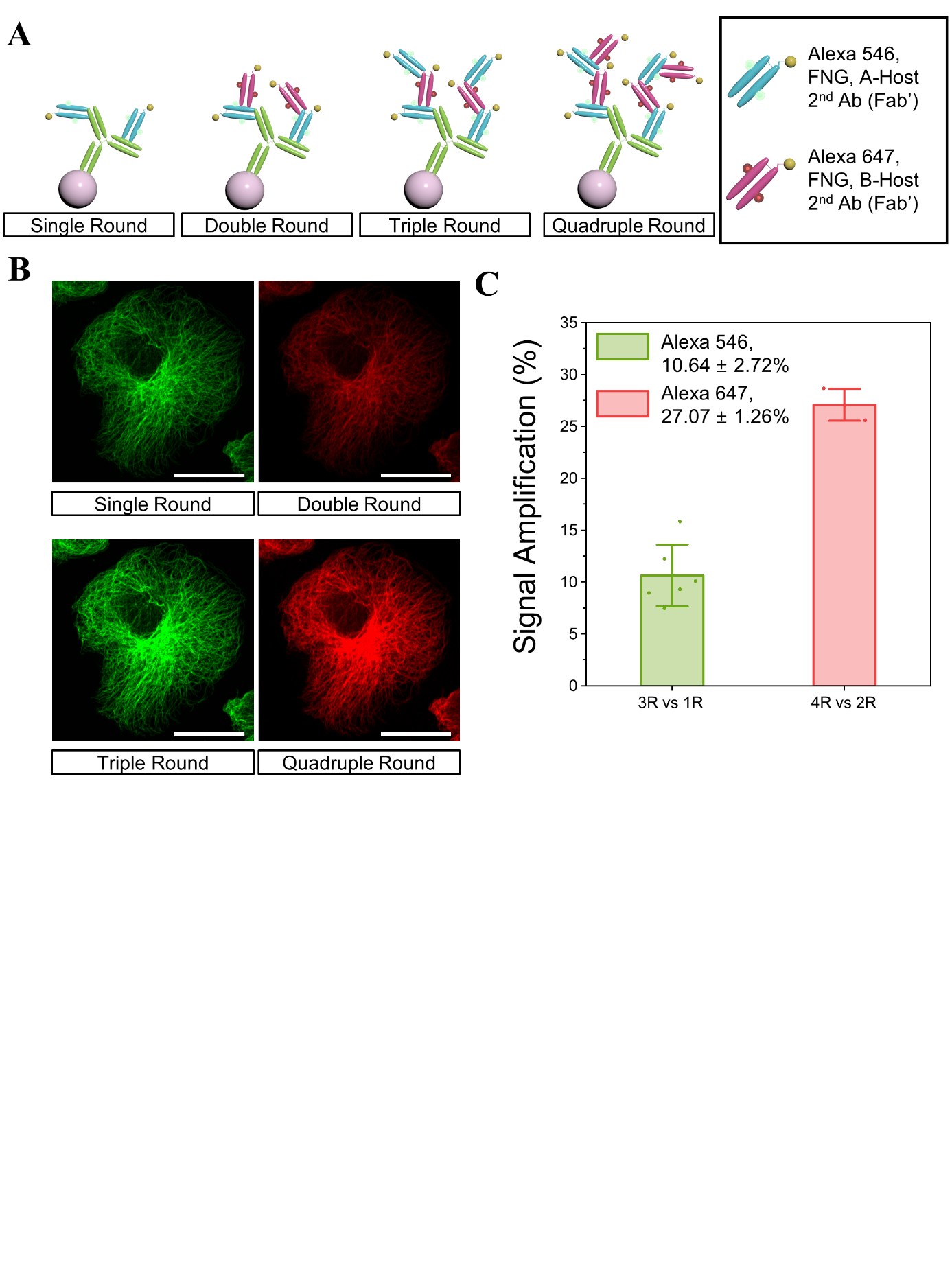


**Fig**. **S18**. **Alternative iterative labeling strategy (1).** (**A**) Schematic of one of the alternative iterative labeling strategies through the introduction of FNG pair, which consists of antibody fragment (Fab'), smaller than whole IgG antibodies. (**B**) Representative images at each labeling round (signal mean values (a.u.); 1R: 462.39, 2R: 509.01, 3R: 384.28, 4R: 713.37). (**C**) Comparison of signal amplification levels by labeling rounds using different fluorophores. Data are presented as mean ± s.d., starting with a total *n* = 12 points from three independent cells in four wells. Scale bar. B. 30 μm.

**
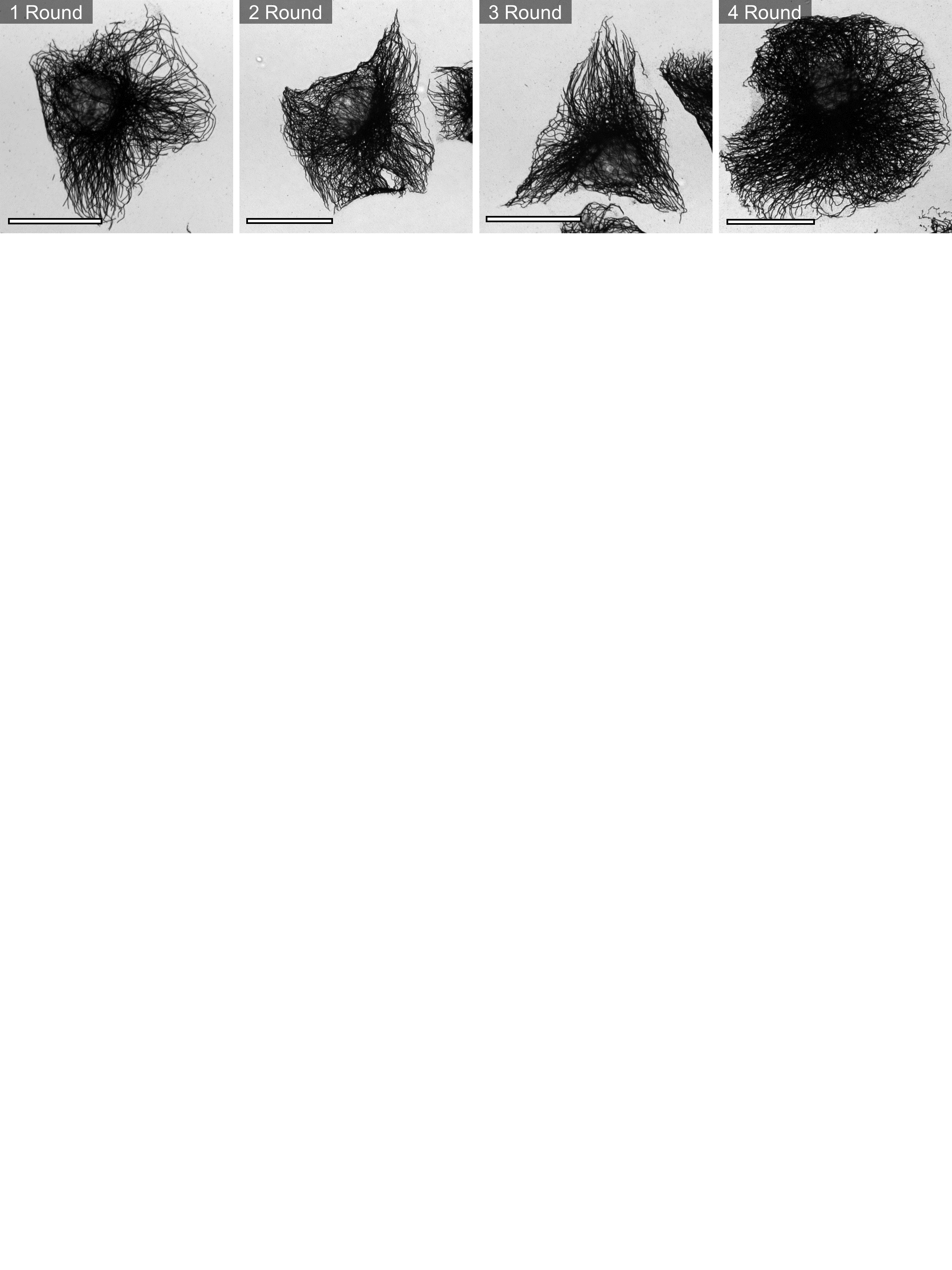
**

**Fig**. **S19**. **BF images from each labeling round of the iterative labeling strategy described in Fig S18.** Scale bar. 30 μm.

**
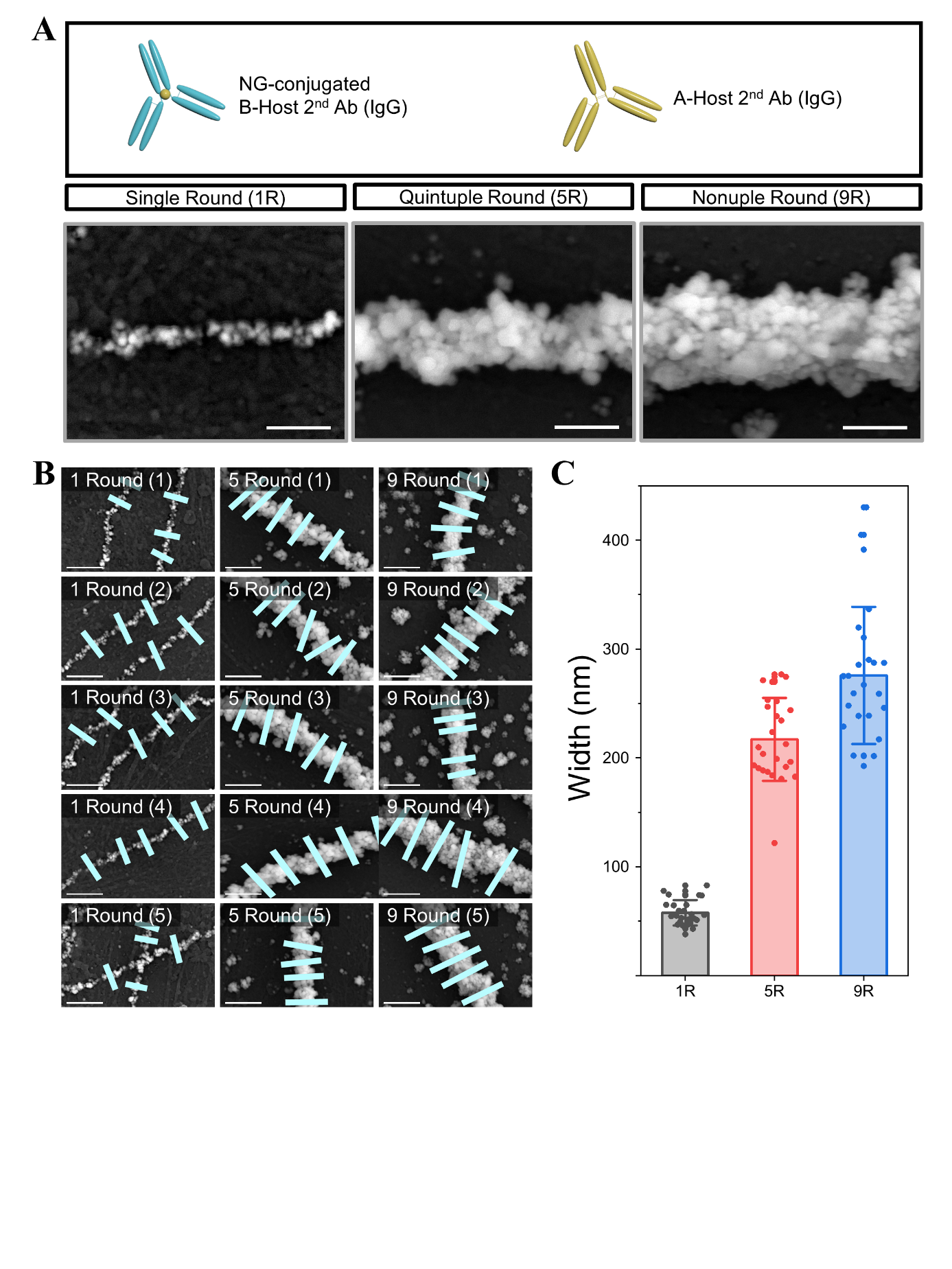
**

**Fig**. **S20**. **Alternative iterative labeling strategy (2).** (**A**) The schematic of the antibody pair consists of a labeling spacer and representative SEM images of multiple rounds. (**B**) SEM images of AgNP chains at each labeling round with blue lines indicating regions used for SEM profile analysis. (**C**) Width comparison graph of AgNP chains *via* FWHM value calculation against the labeling round (average width; 1R: 57.9 nm / 5R: 217.1 nm / 9R: 275.8 nm). Data are presented as mean ± s.d., starting with a total *n* = 25 measurements from independent regions of two different cells each in a single substrate. Scale bar. A. 300 nm, B. 500 nm.


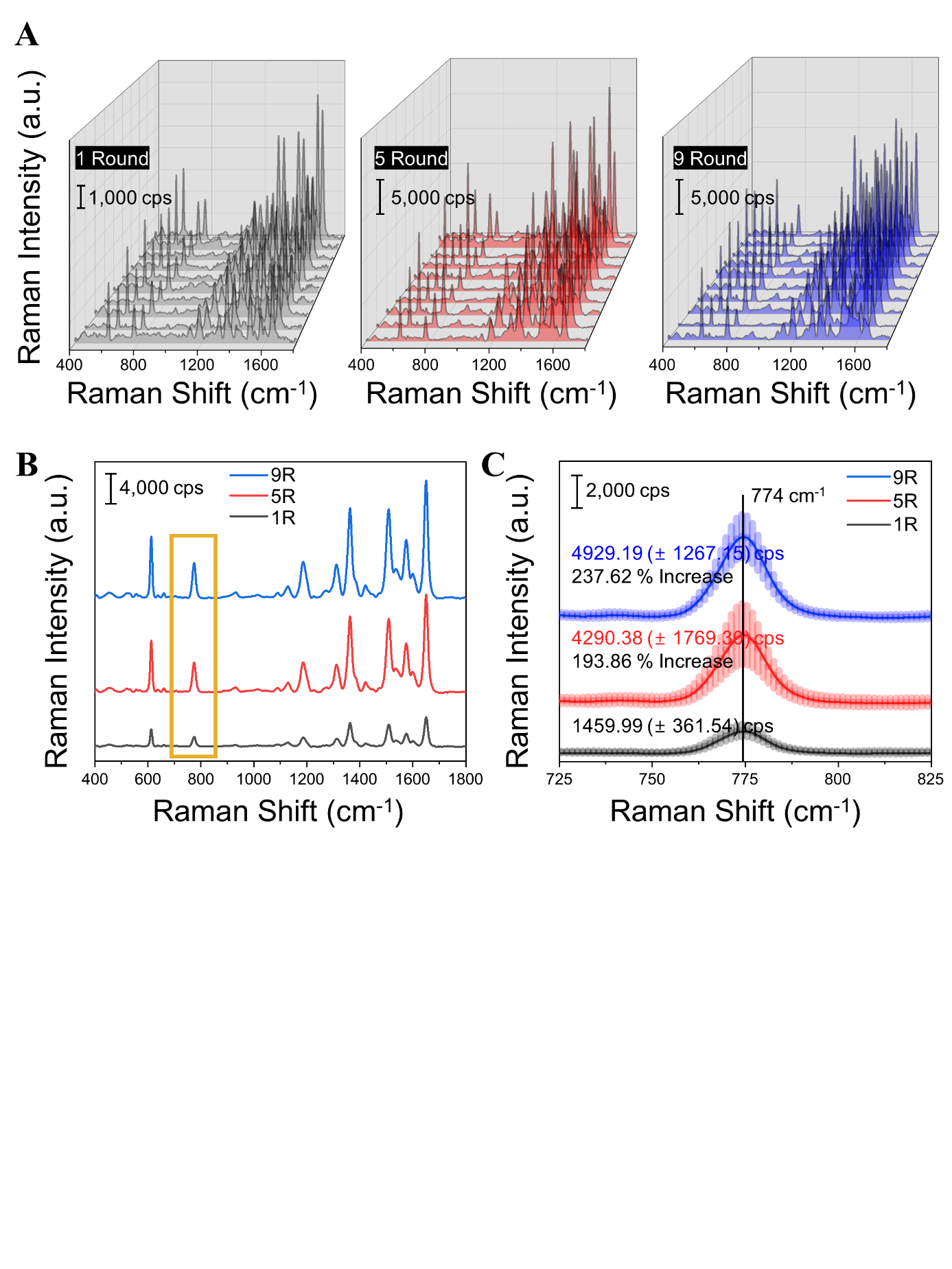


**Fig**. **S21**. **SERS spectra of R6G on silver-grown cell substrate according to the labeling round in Fig. S18**. (**A**) Reproducible SERS spectra and (**B**) average SERS spectra of R6G on silver-grown cell substrate according to the labeling round. (**C**) Raman intensity comparison at a representative peak (774 cm^-1^) range of R6G with average SERS spectra of each labeling round. Data are presented as mean ± s.d., *n* = 10 points from ten independent cells in a single substrate.


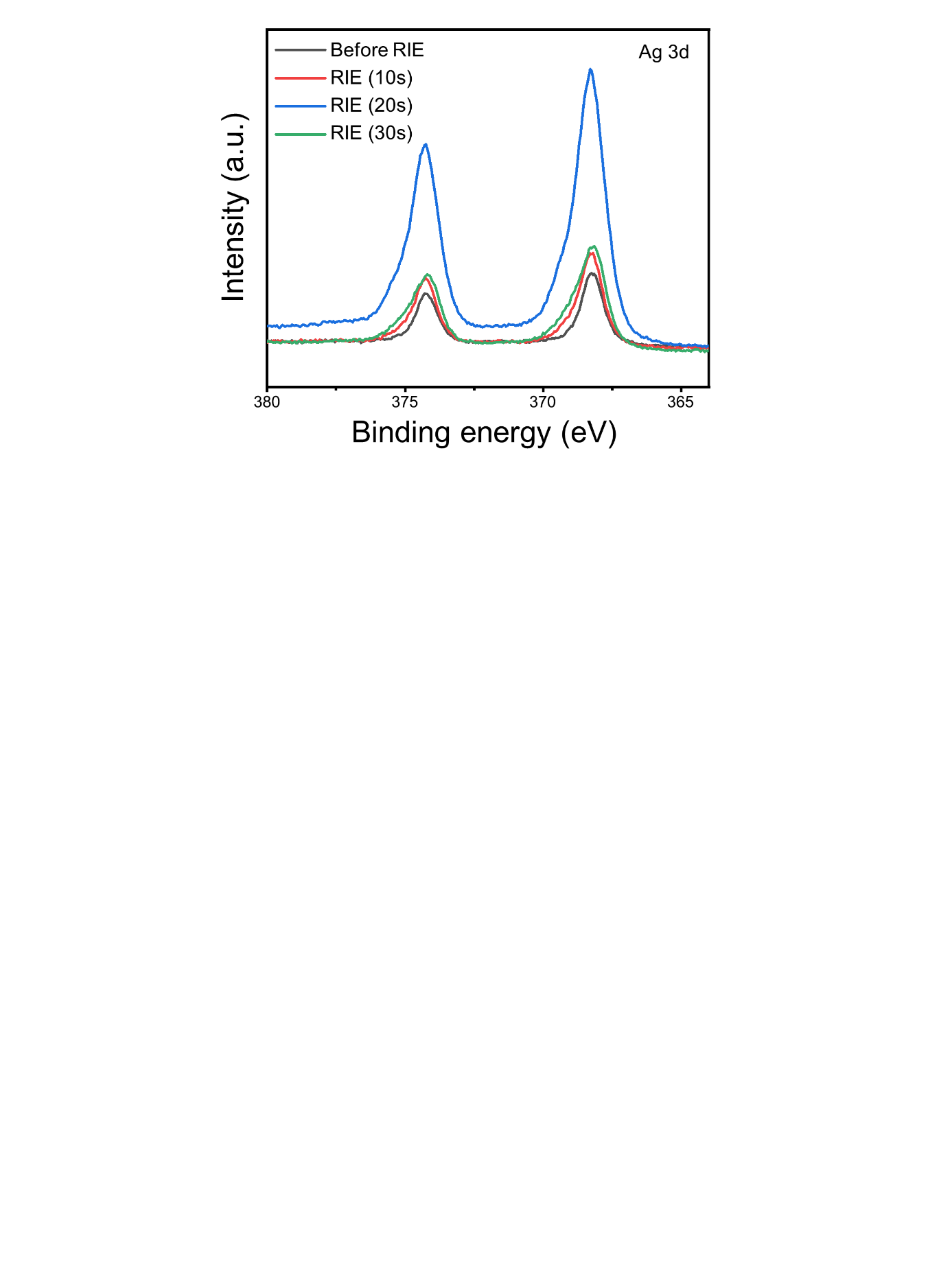


**Fig**. **S22**. **Ag 3d XPS spectra of silver-grown cell substrate according to RIE processing time.**


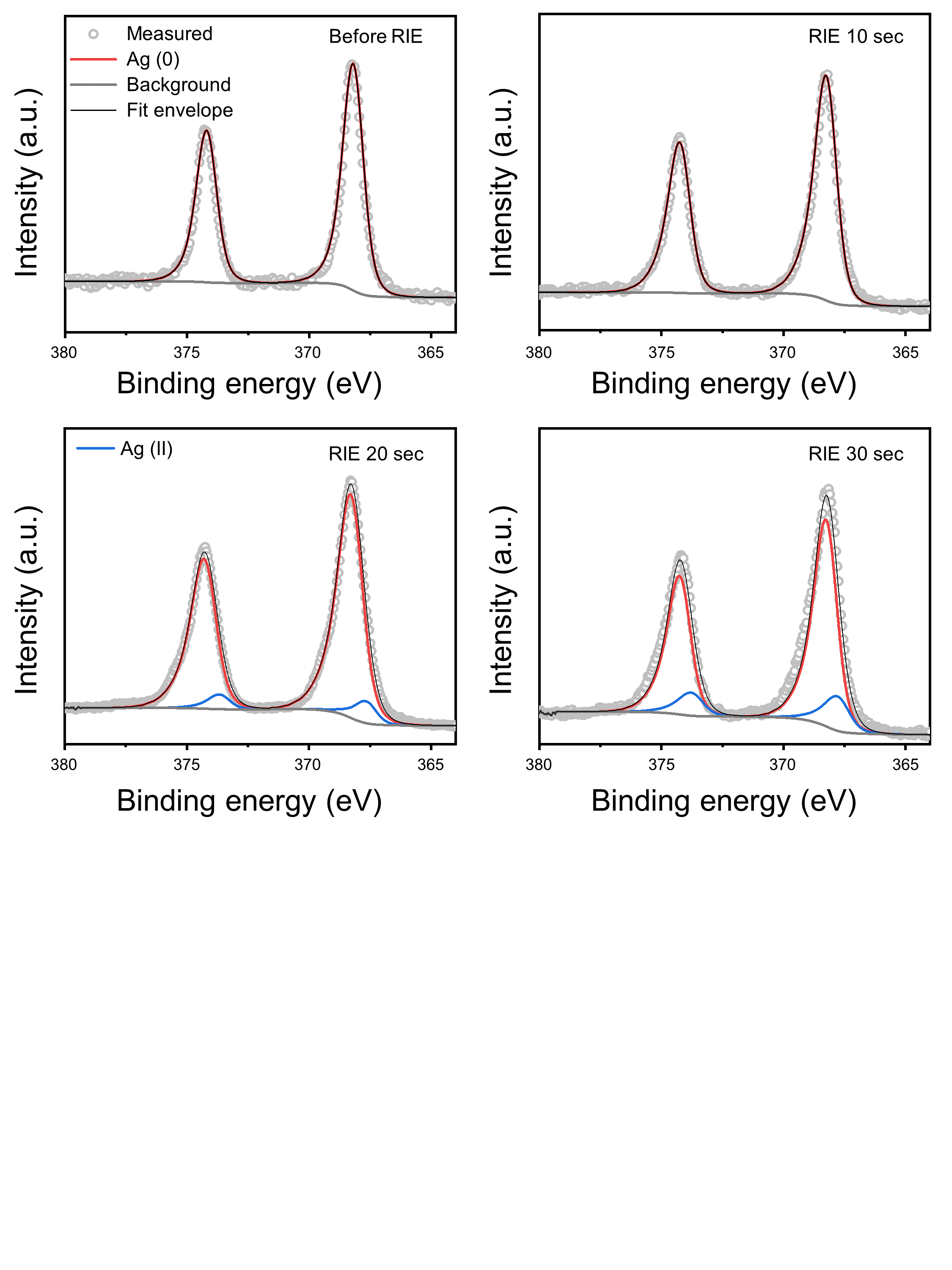


**Fig**. **S23**. **Detailed Ag 3d XPS spectra of silver-grown cell substrate according to RIE processing time.**


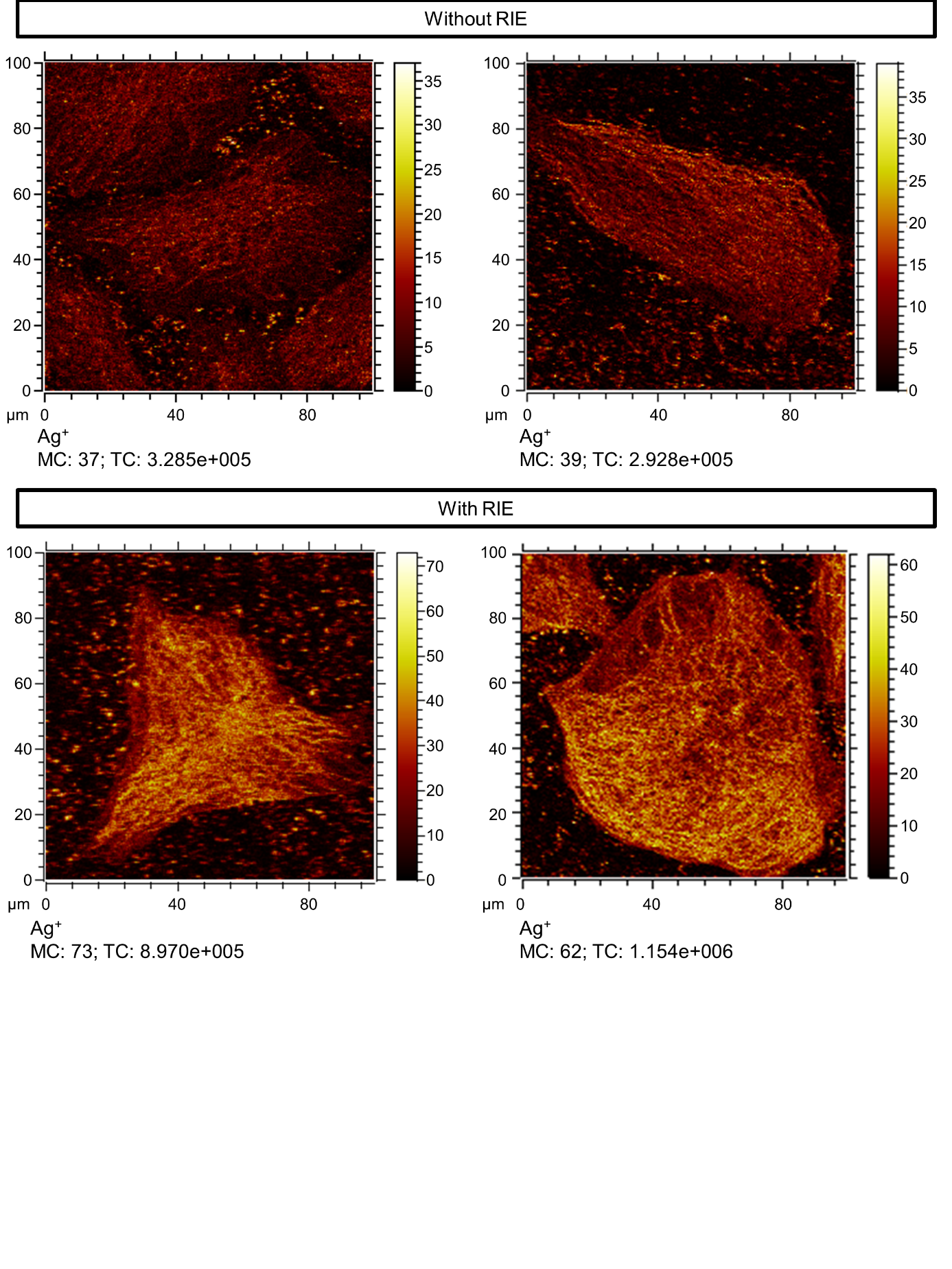


**Fig**. **S24**. **Additional ToF-SIMS mapping images of silver-grown cells according to the RIE process.**

**
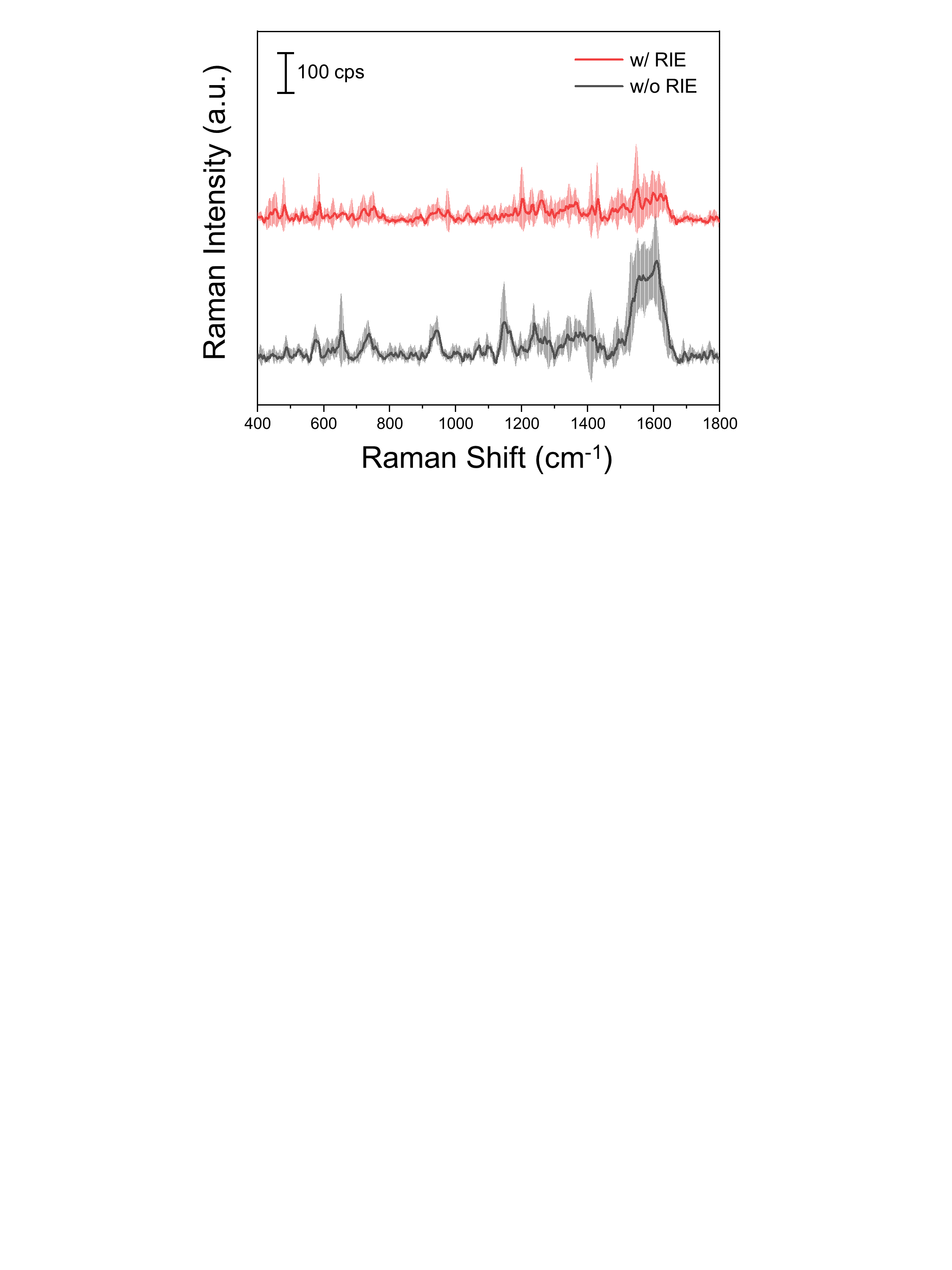
**

**Fig**. **S25**. **Average Raman spectra of silver-grown cell substrates according to the RIE process.** Data of each step are presented as mean ± s.d., *n* = 7 points from seven independent cells in each case in a single substrate.


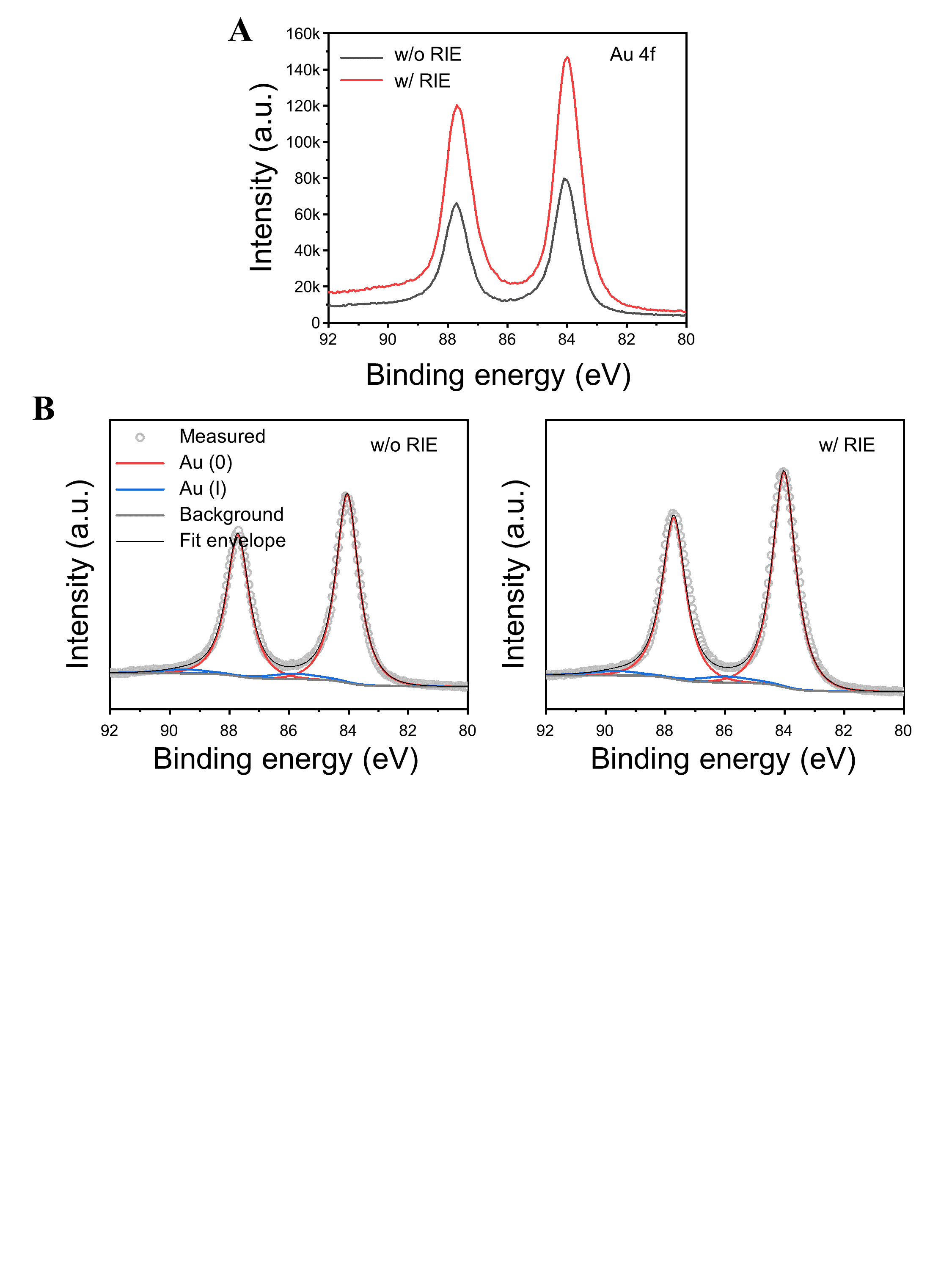


**Fig**. **S26**. **Au 4f XPS spectra of gold-grown cell substrates.** (**A**) Au 4f XPS spectra of gold-grown cell substrate according to RIE process. (**B**) Detailed Au 4f XPS spectra of gold-grown cell substrate according to RIE process.

**
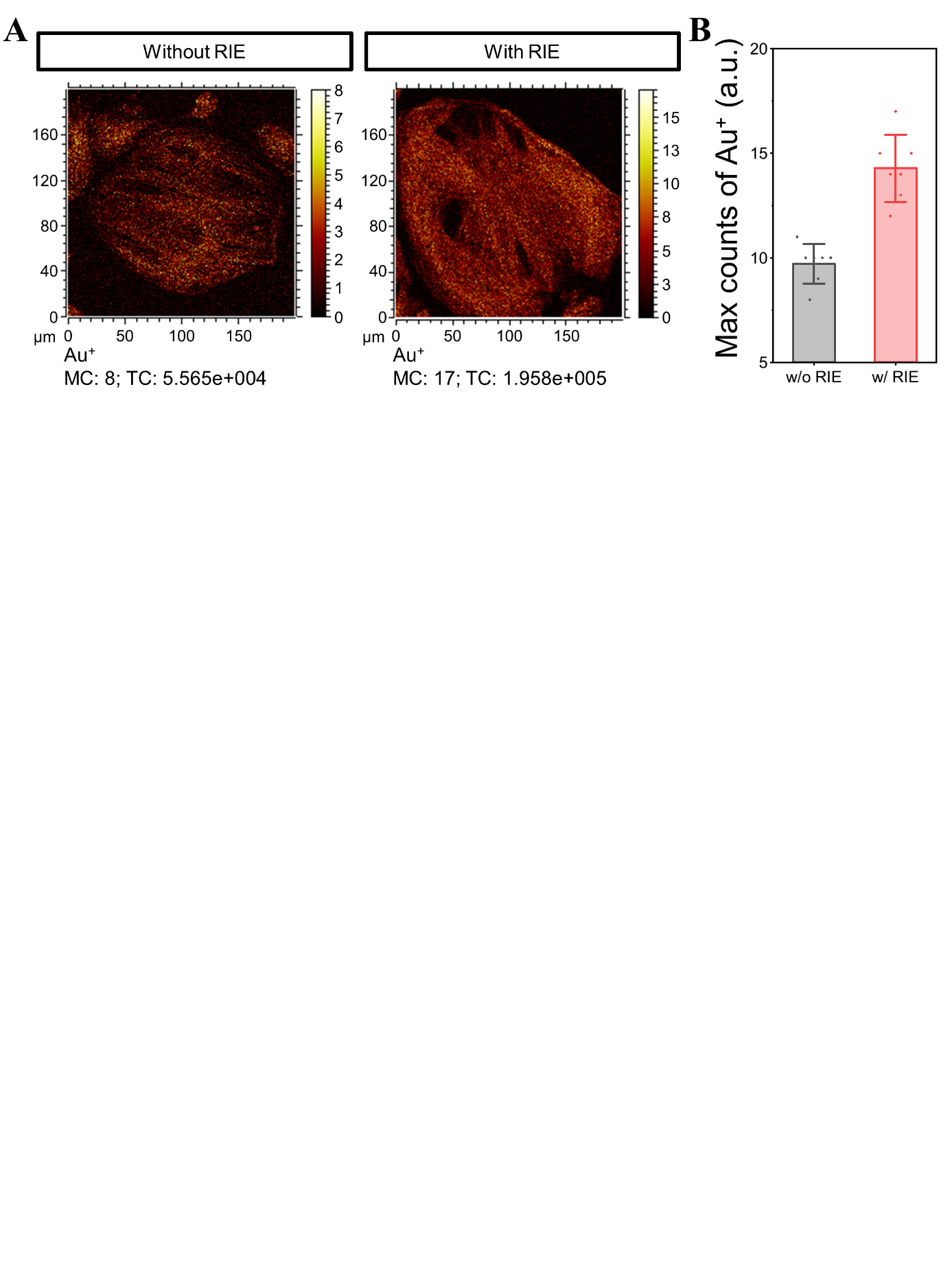
**

**Fig**. **S27**. **ToF-SIMS analysis of gold-grown cell substrates.** (**A**) Representative ToF-SIMS mapping images of gold-grown cells according to the RIE process. (**B**) Max counts comparison of gold ion against RIE (average max count, MC (a.u.); without: 9.71 / with: 14.29). Data of each sample are presented as mean ± s.d., *n* = 7 from independent regions in a single substrate.

**
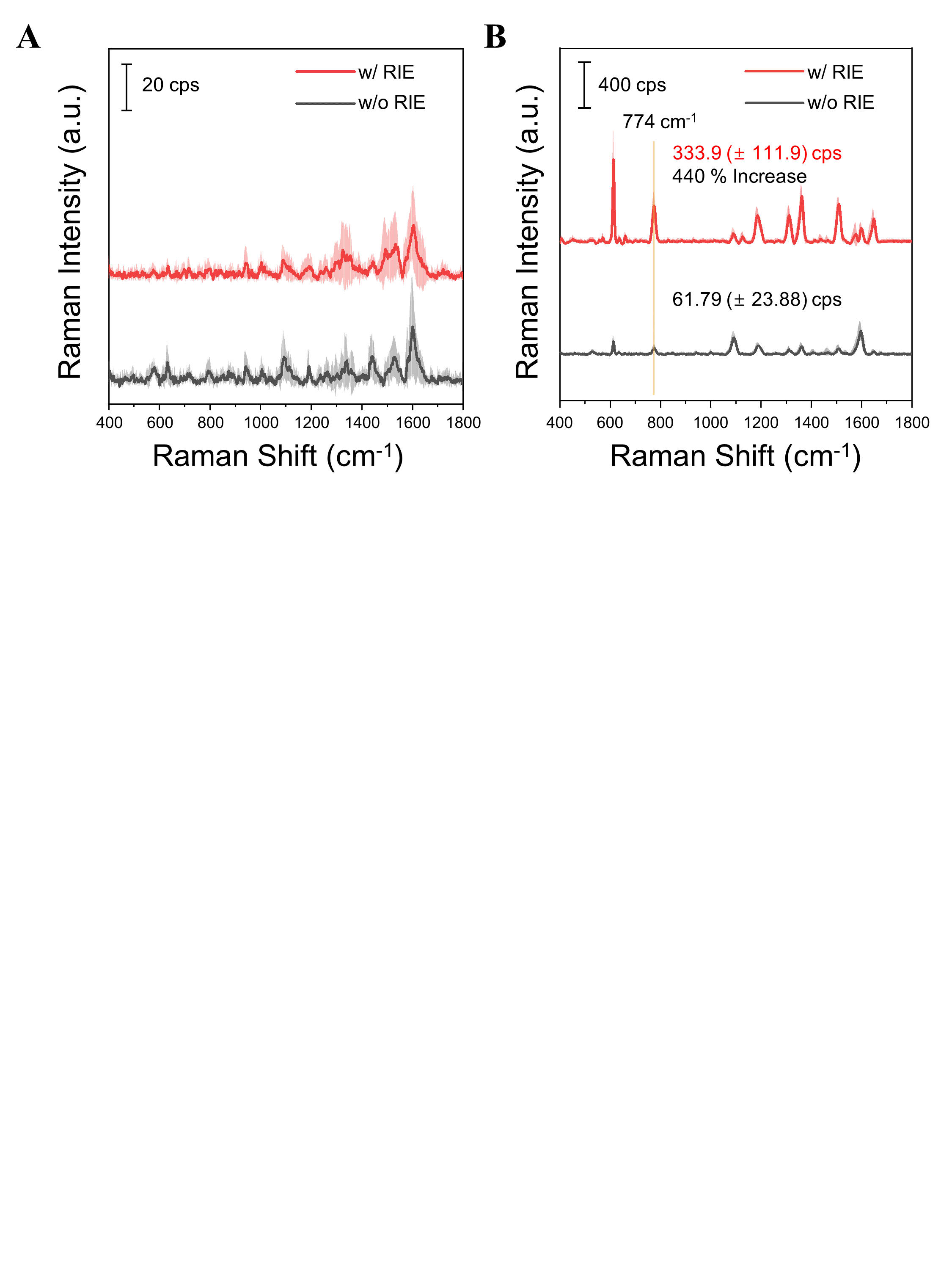
**

**Fig**. **S28**. **Raman and SERS spectra of gold-grown cell substrates.** (**A**) Average Raman spectra of gold-grown cell substrates according to the RIE process. (**B**) Average SERS spectra of R6G recorded on gold-grown cell substrate against RIE process. Data of each sample are presented as mean ± s.d., *n* = 7 points from seven independent cells in each case in a single substrate.

**
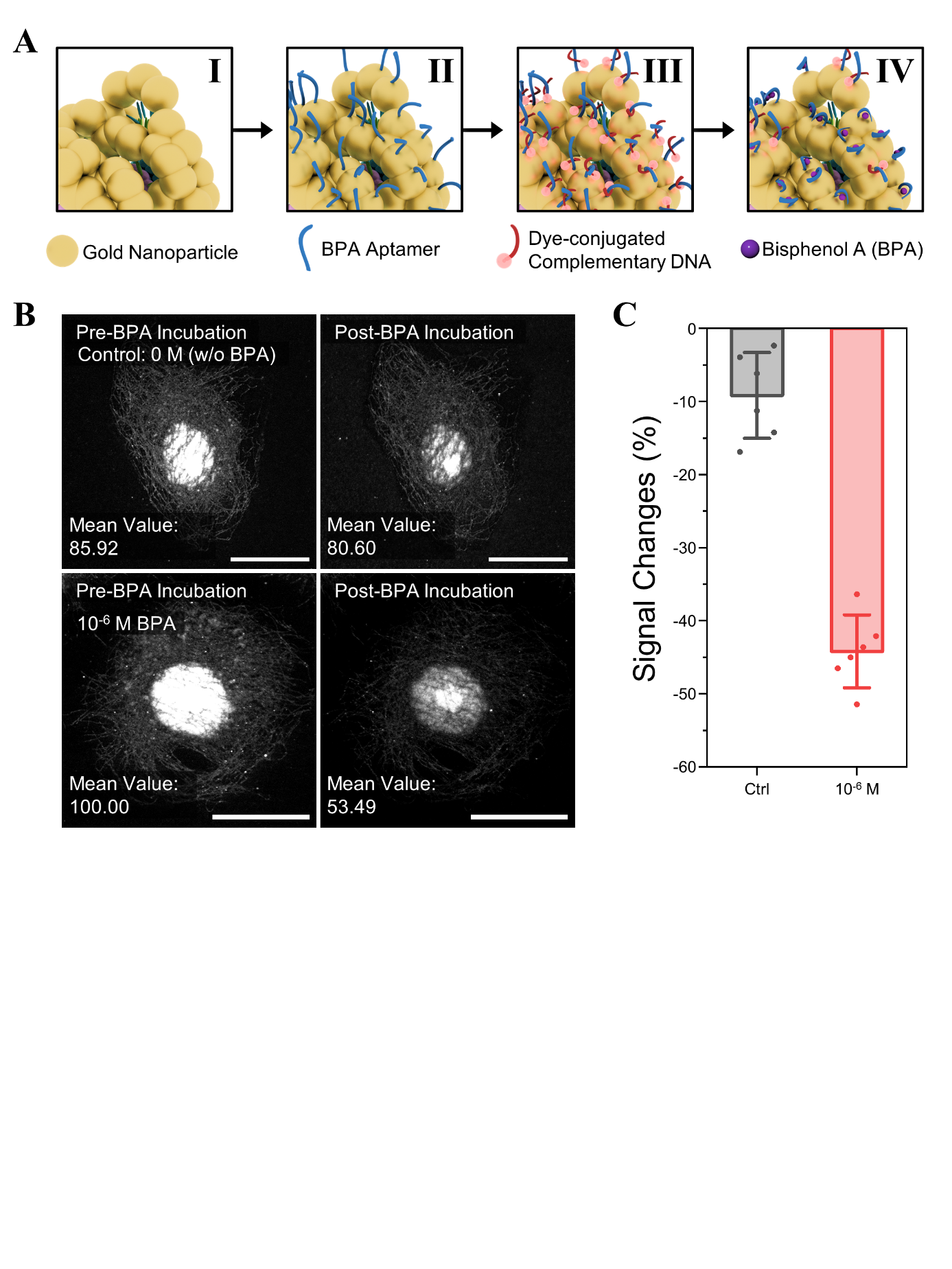
**

**Fig**. **S29**. **Aptamer functionalization of AuNP chains after structure extraction through the RIE process**. (**A**) Schematic describing the aptamer functionalization process and capturing of BPA. (**I**) AuNP chains converted from microtubule structures (**II**) BPA aptamer functionalized AuNP chains. (**III**) Dye-conjugated BPA complementary ssDNA bound to AuNP chains. (**IV**) BPA captured by functionalized AuNP chains. (**B**) Representative FM images of pre- and post-BPA incubation with mean values. (**C**) Comparison of signal changes after BPA incubation. Data are presented as mean ± s.d., *n* = 3 per well from two independent wells. Scale bar. B. 30 μm.

**
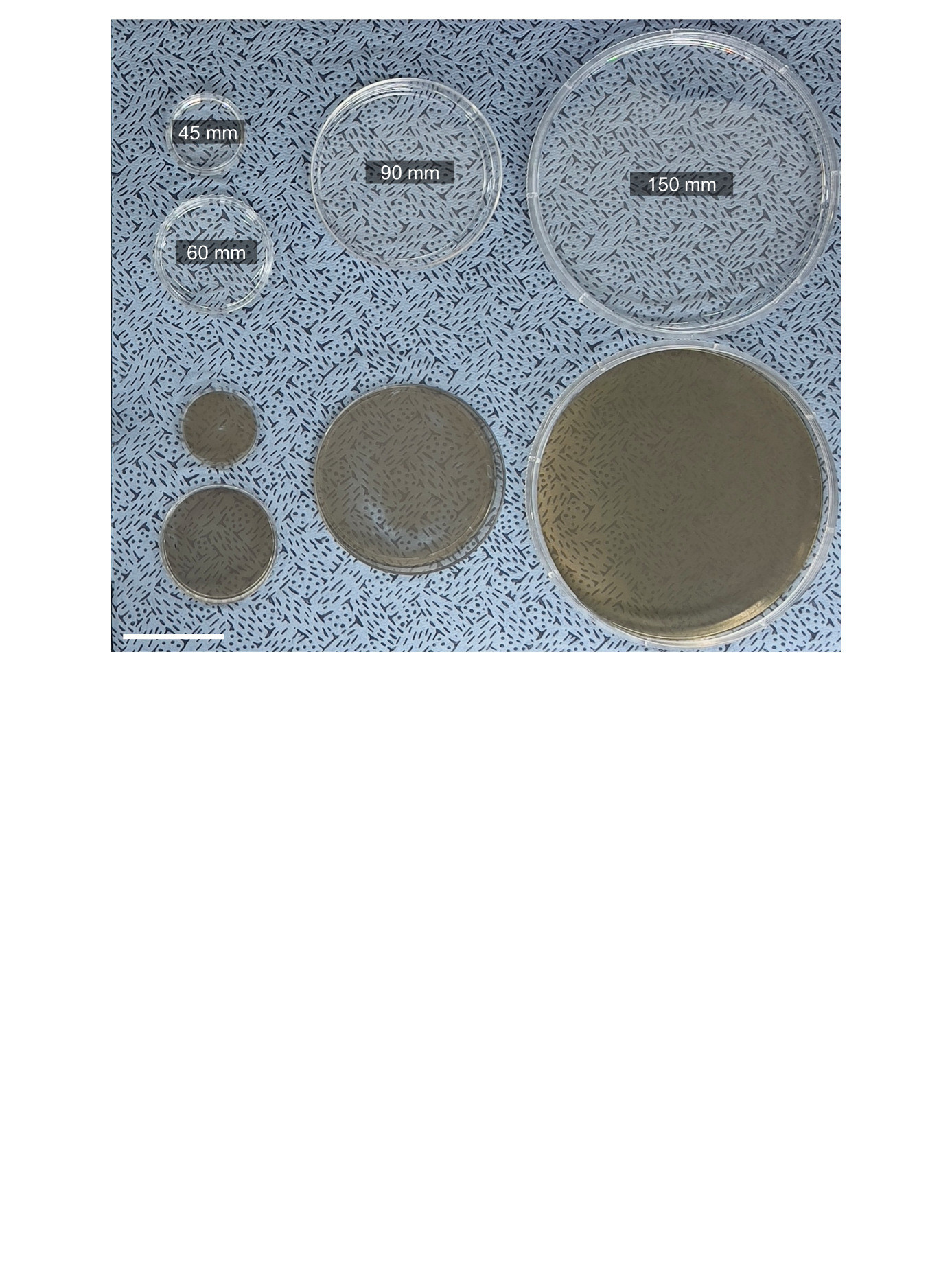
**

**Fig**. **S30**. **Massive production according to cell culture area *via* a proliferation of adherent cells.** Various cell culture dishes wherein adherent cells were cultured (top line) and were silver grown along with microtubules inside cells *via* the CamBio process (bottom line). Scale bar. 50 mm.

**
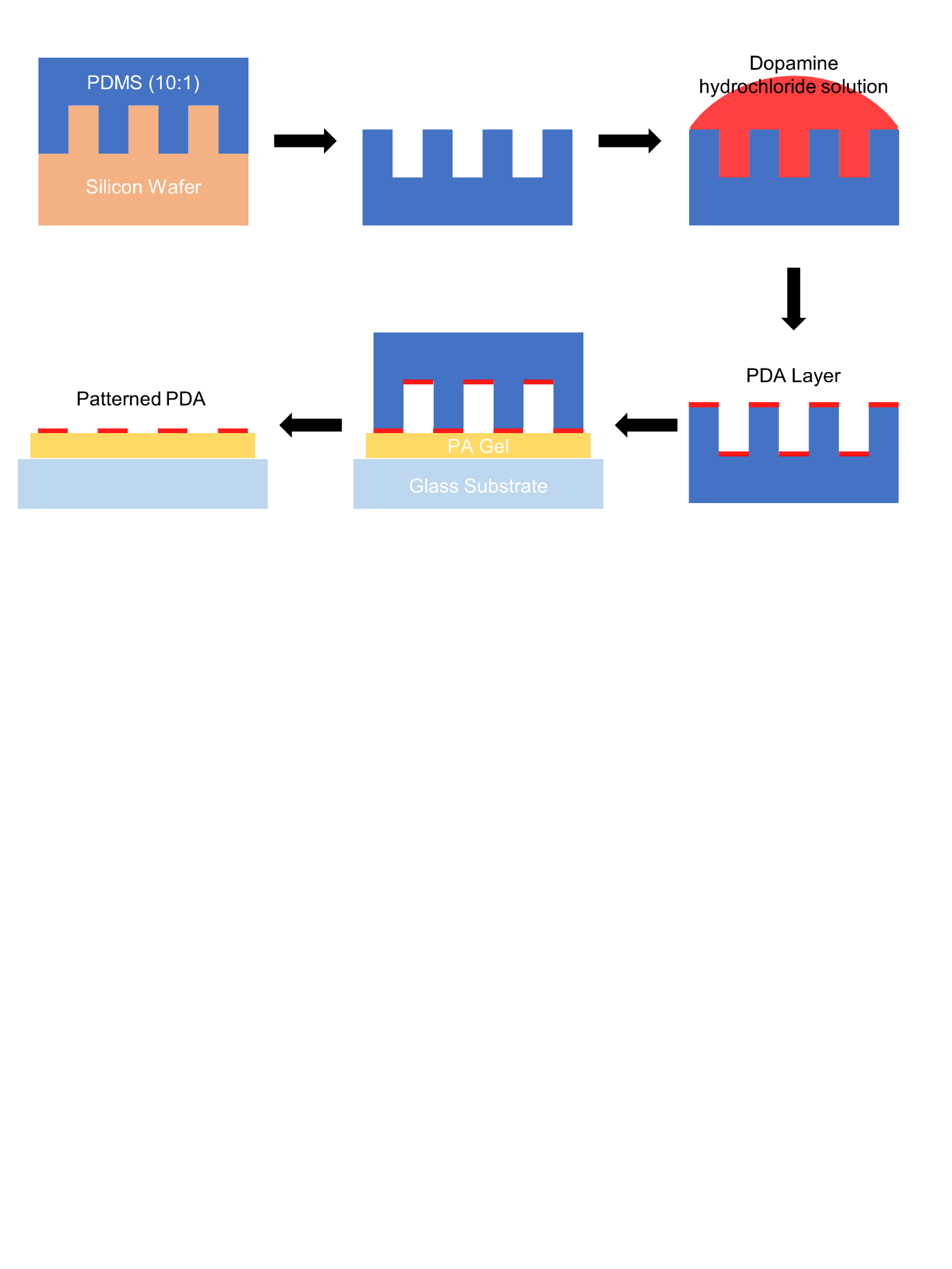
**

**Fig**. **S31**. **Schematic of a method to fabricate PDA patterned PA hydrogel substrate for cell patterning.**

**
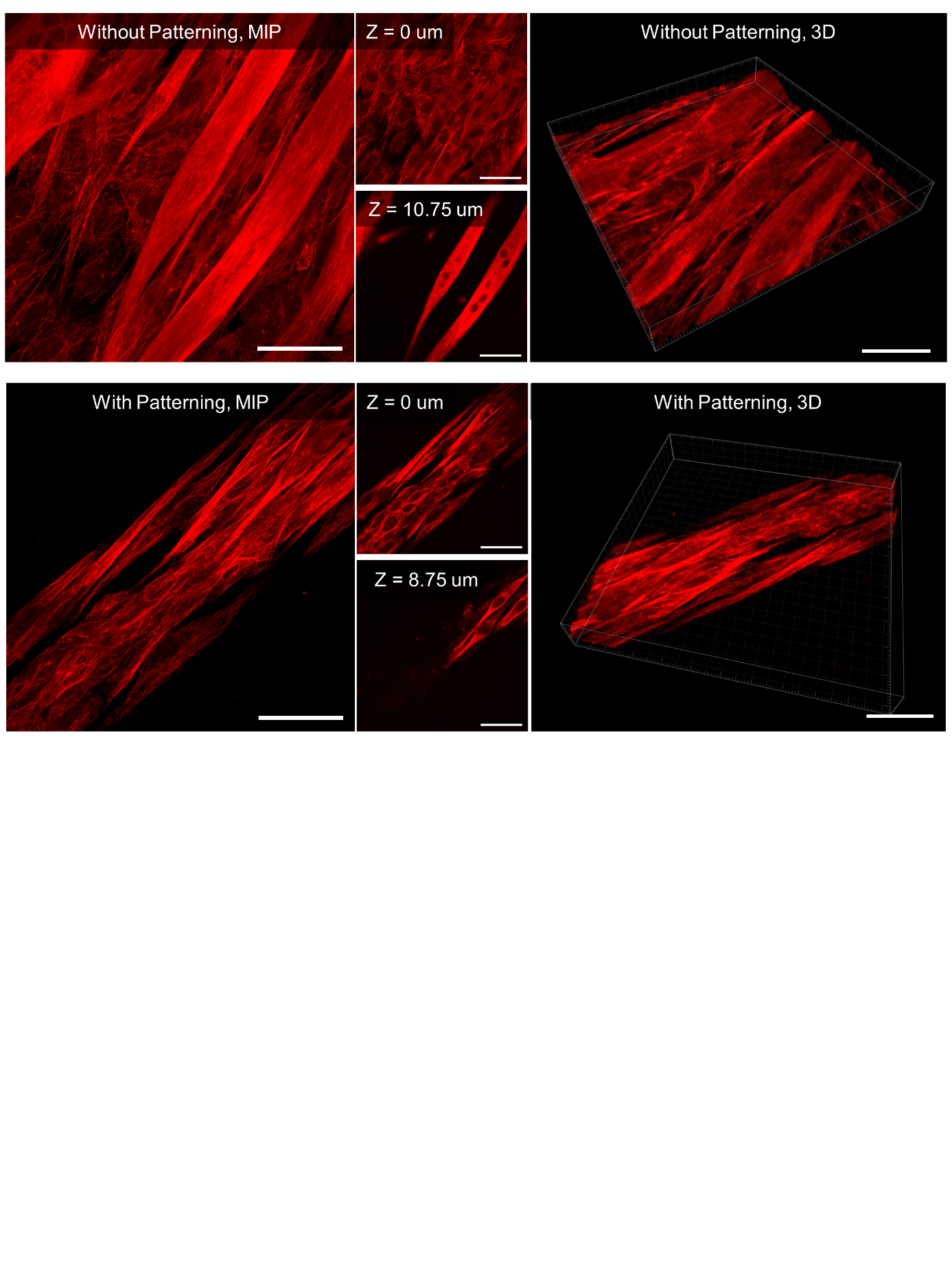
**

**Fig**. **S32**. **FM images of microtubule labeled cells according to patterning** (100 μm linear pattern). Scale bar. 50 μm.

**
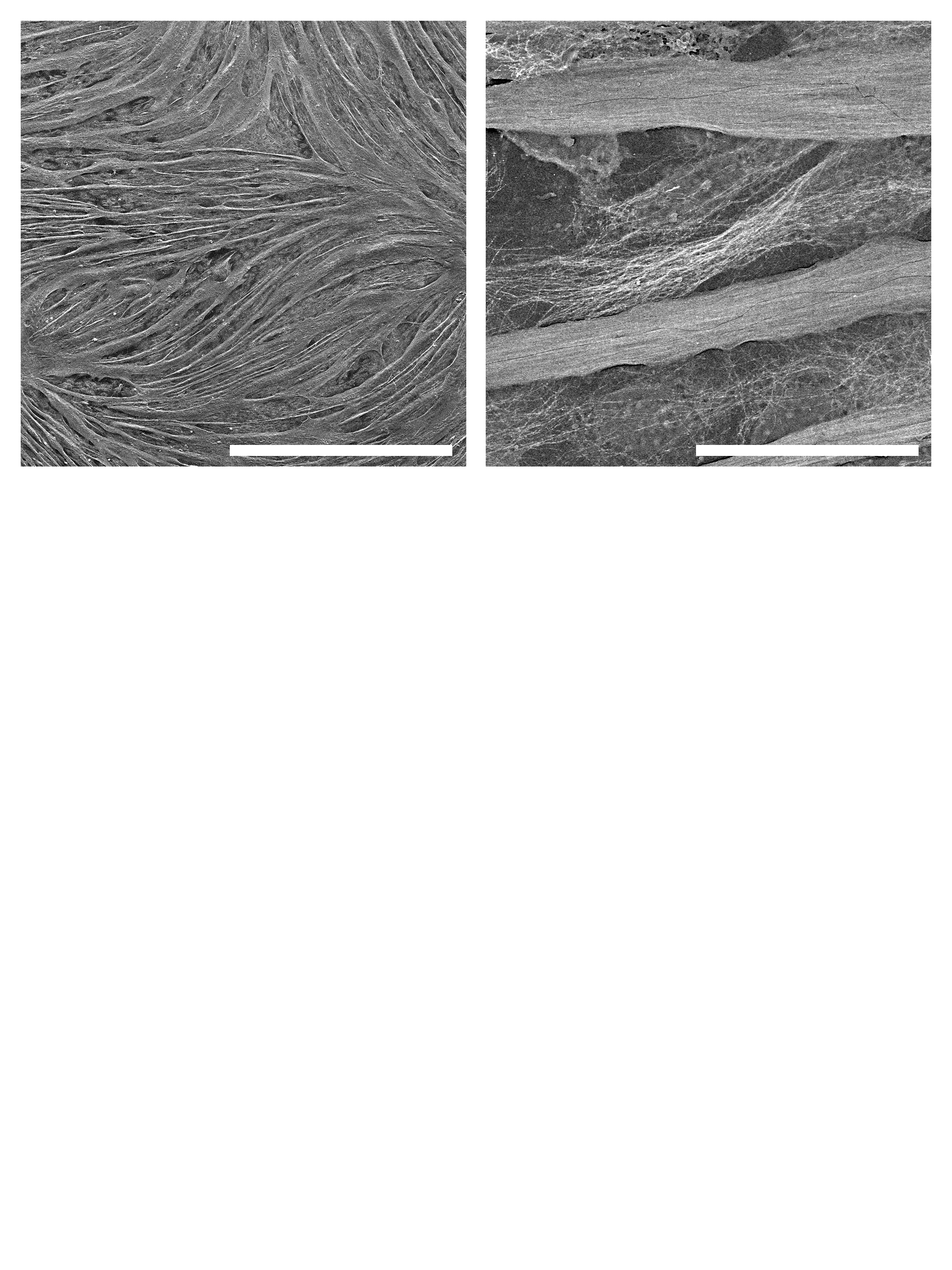
**

**Fig. S33. Additional SEM images of cells that were silver grown at labeled microtubules** (left: low magnified, right: high magnified). Scale bar. 1 mm (left), 40 μm (right).

**
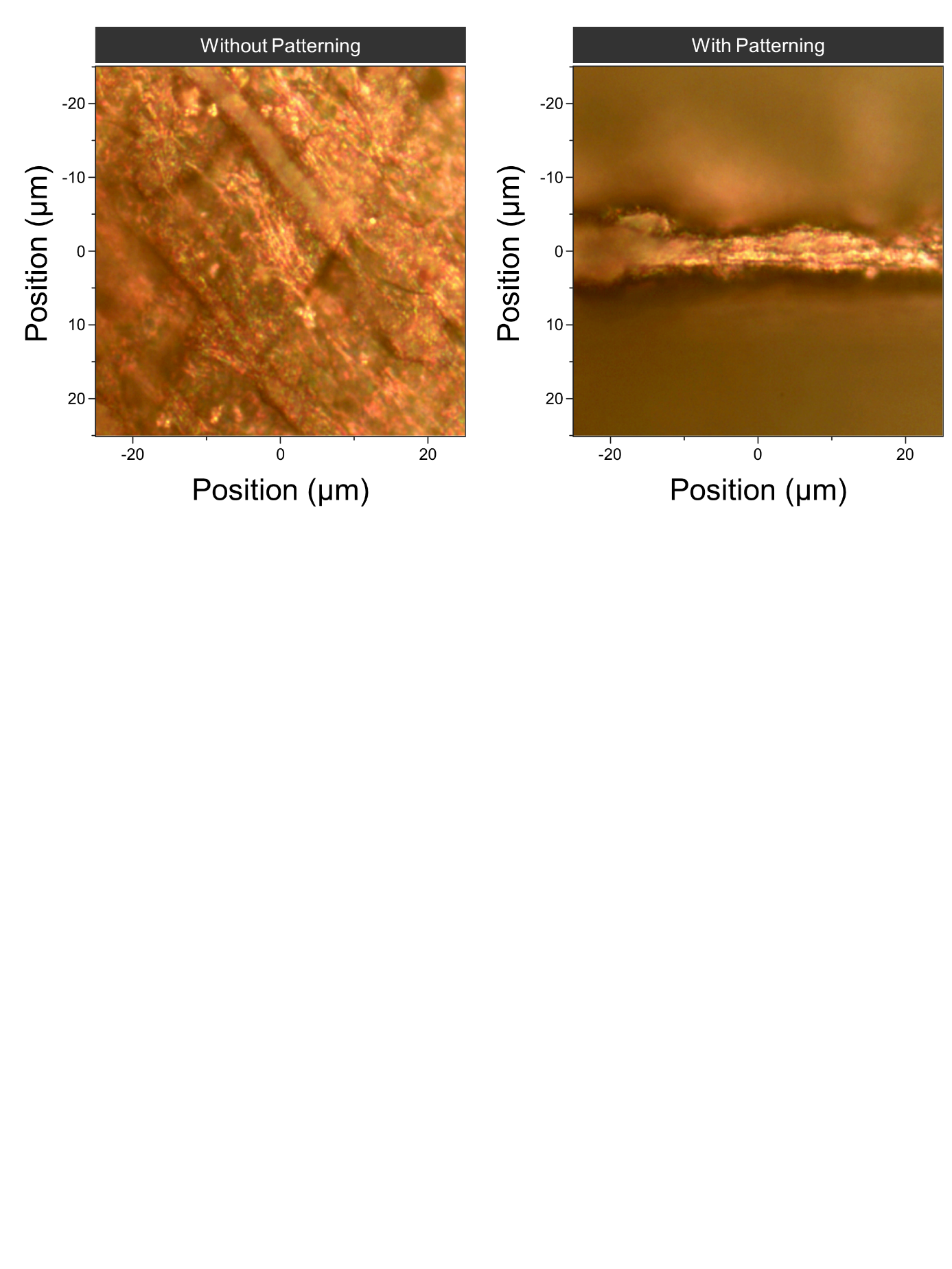
**

**Fig**. **S34**. **Optical images at Raman mapping region.**

**
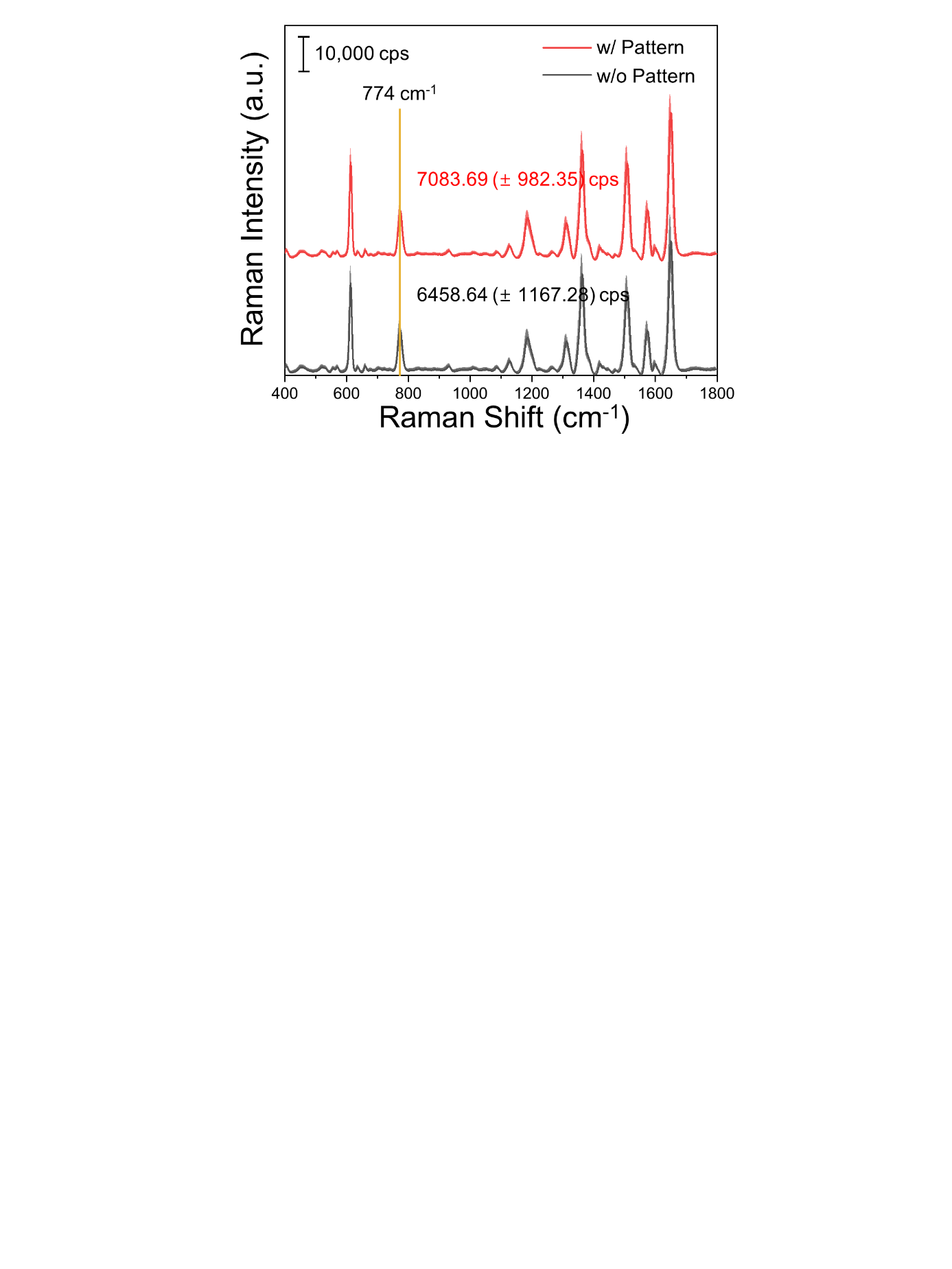
**

**Fig**. **S35**. **SERS spectra of R6G recorded on silver-grown cell substrate according to patterning**. Data of each sample are presented as mean ± s.d., *n* = 8 points from eight independent cells in each case in a single substrate.


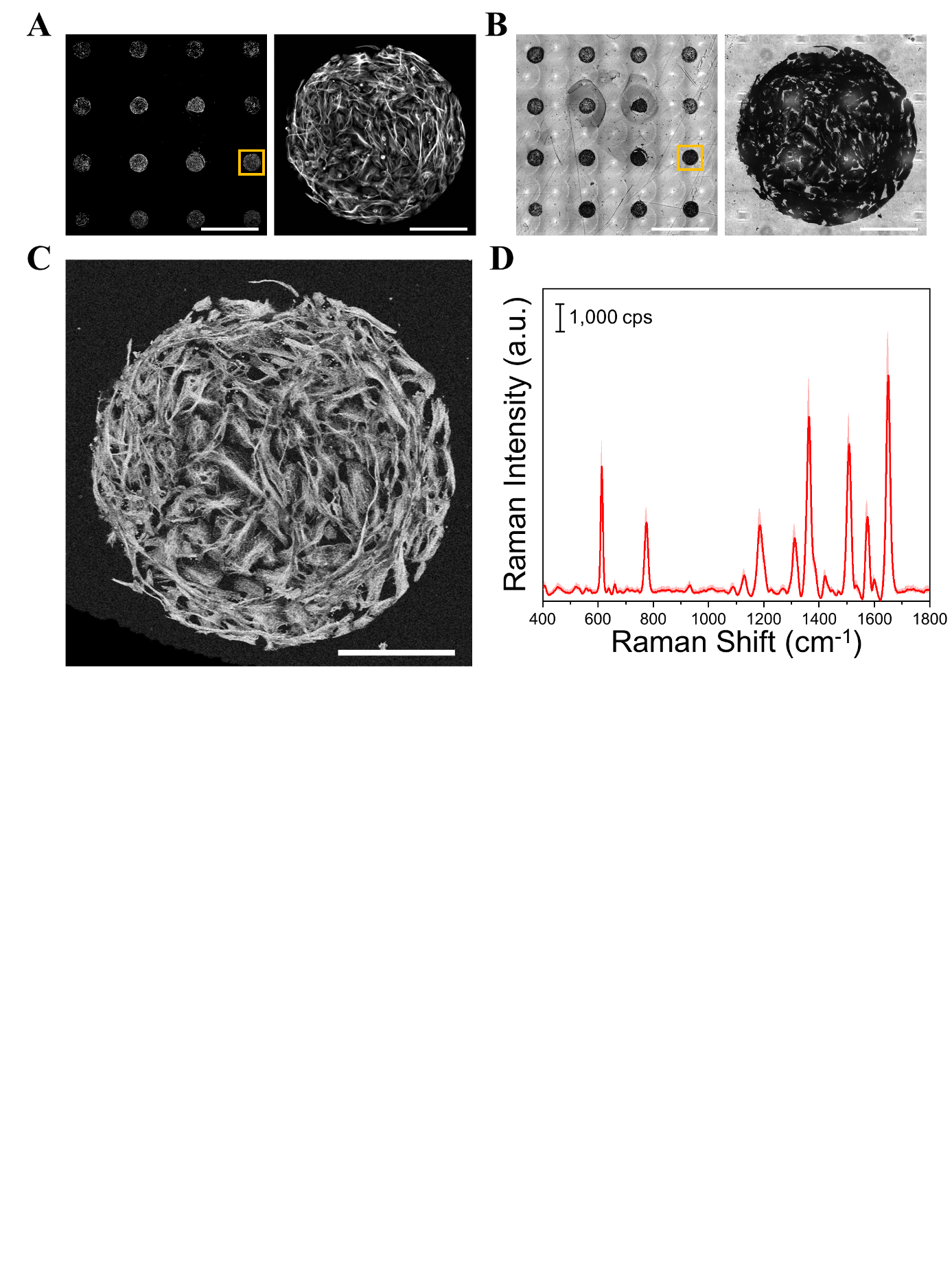


**Fig**. **S36**. **Images of microtubule-labeled circular patterned (700 μm) epithelial cells.** (**A**) FM images of circular patterned cells and (**B**) BF images of silver-grown cells. Right side images show magnified images in a yellow boxed region in each left image. (**C**) SEM image of silver-grown cells in yellow box region above. (**D**) Average SERS spectra of R6G recorded on the silver-grown circular patterned cell substrate. Data are presented as mean ± s.d., *n* = 5 points from five independent patterns in a single substrate. Scale bar. A, B. 2 mm (left), 200 μm (right), C. 200 μm.


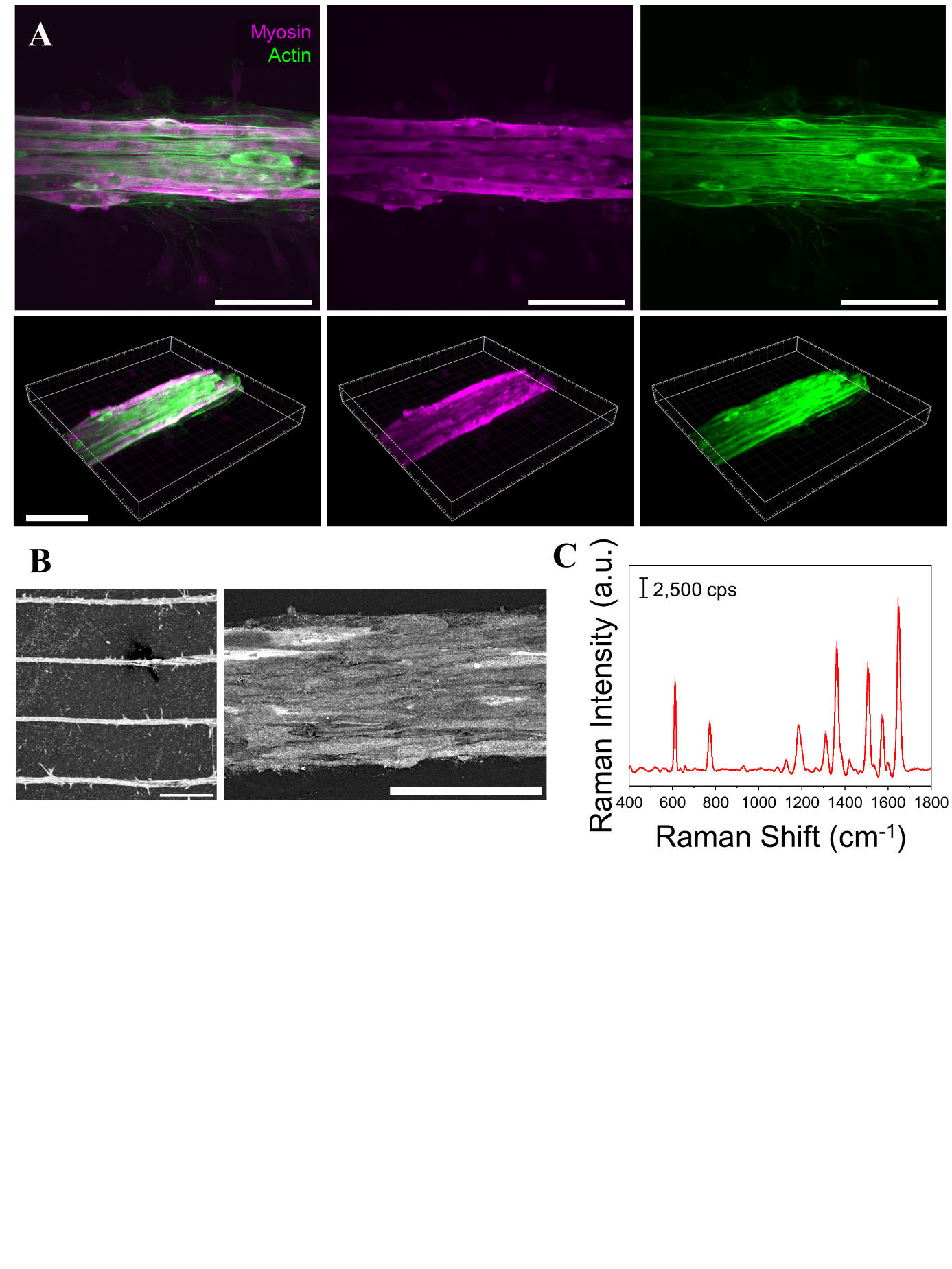


**Fig**. **S37**. **Myosin and actin-labeled, line-patterned (100 μm) myoblast cells.** (**A**) FM images of myosin and actin labeled, line-patterned myoblast cells (magenta: myosin, green: actin). (**B**) SEM images of line-patterned myoblast cells were silver-grown from myosin proteins. (**C**) Average SERS spectra of R6G recorded on the silver-grown linear patterned cell substrate. Data are presented as mean ± s.d., *n* = 5 points from five independent line patterns in a single substrate. Scale bar. A. 100 μm, B. 500 μm (left), 50 μm (right).


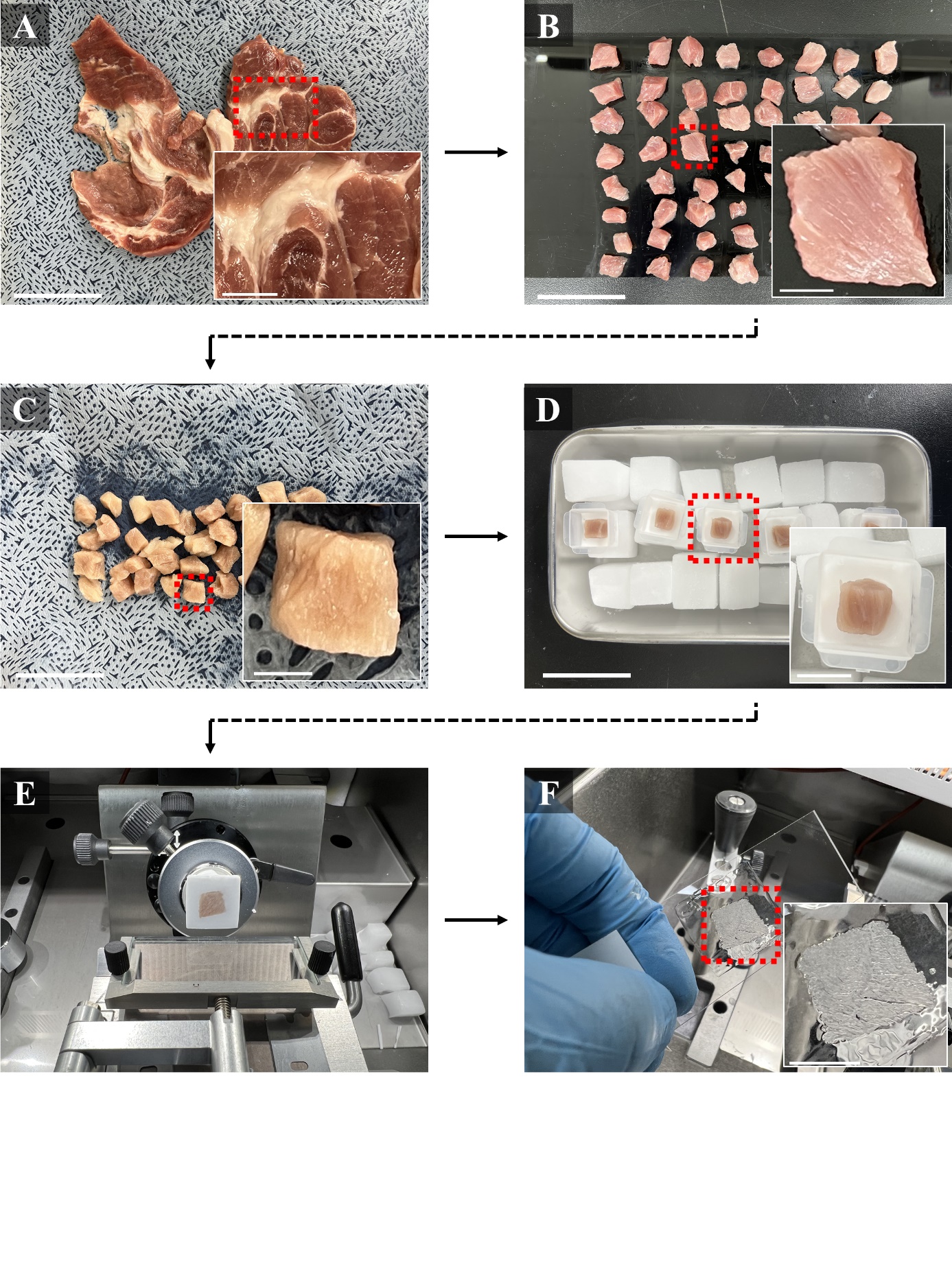


**Fig**. **S38**. **Cryo-sectioning process to obtain thin muscle tissue substrate from pork shoulders.** (**A**) Pork shoulder meats, including fats and fasciae. (**B**) Trimmed and PBS-washed meat samples. (**C**) Fixed and sucrose incubated meat samples. (**D**) OCT compound embedding process. (**E**) Positioned OCT compound at cryostat. (**F**) A thin-sectioned meat slice sample. Detailed procedure-related meat samples were written in the methods section '**Meat samples fixation, cryo-sectioning, and staining.**' Scale bar. A. 5 cm (Inset. 1 cm), B, C. 5 cm (Inset. 5 mm), D. 5 cm (Inset. 1cm), F. 1 cm.


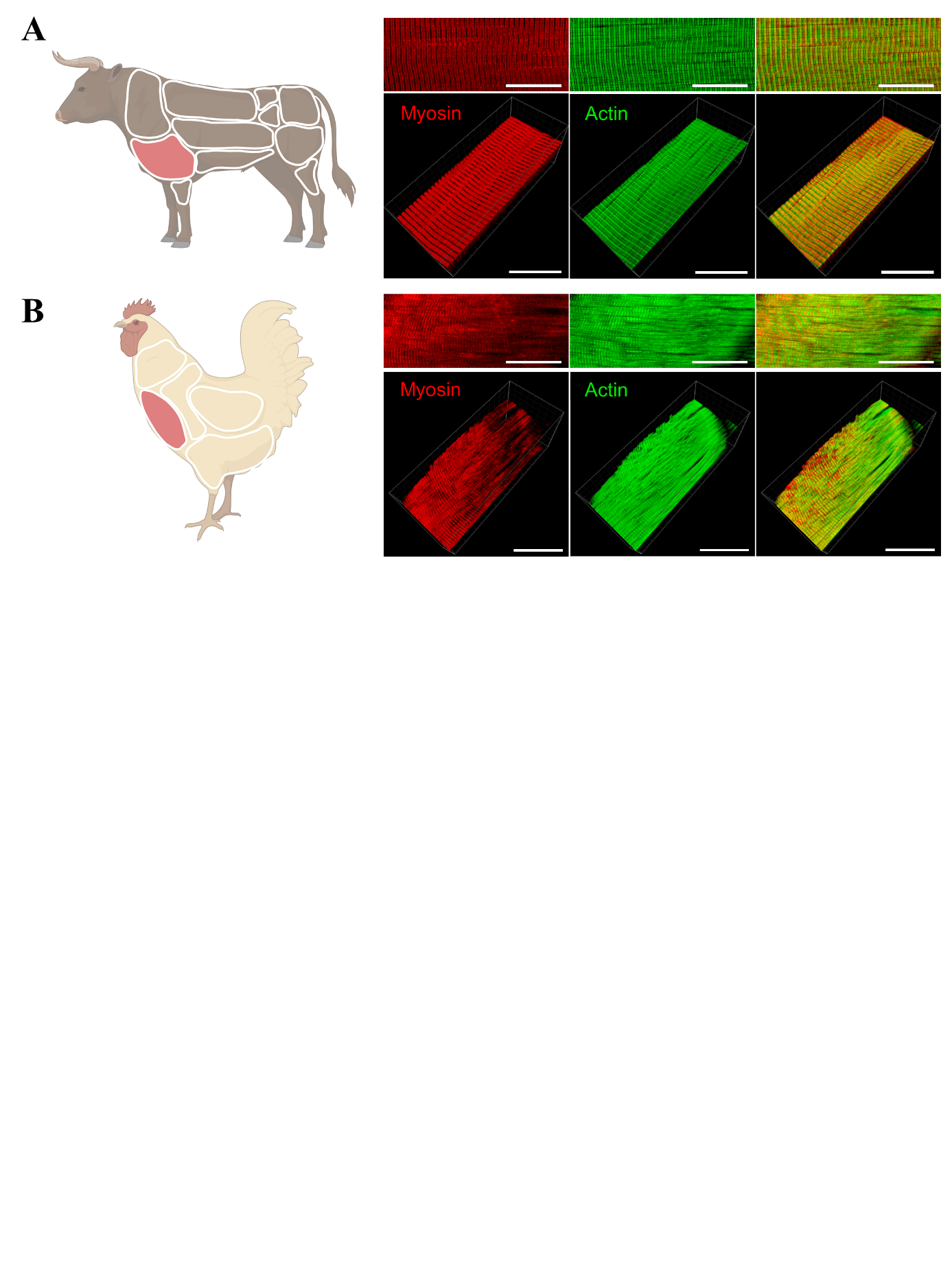


**Fig**. **S39**. **FM images of muscle proteins in various meat samples.** (**A**) Beef brisket, (**B**) Chicken brisket. Scale bar. 30 μm.


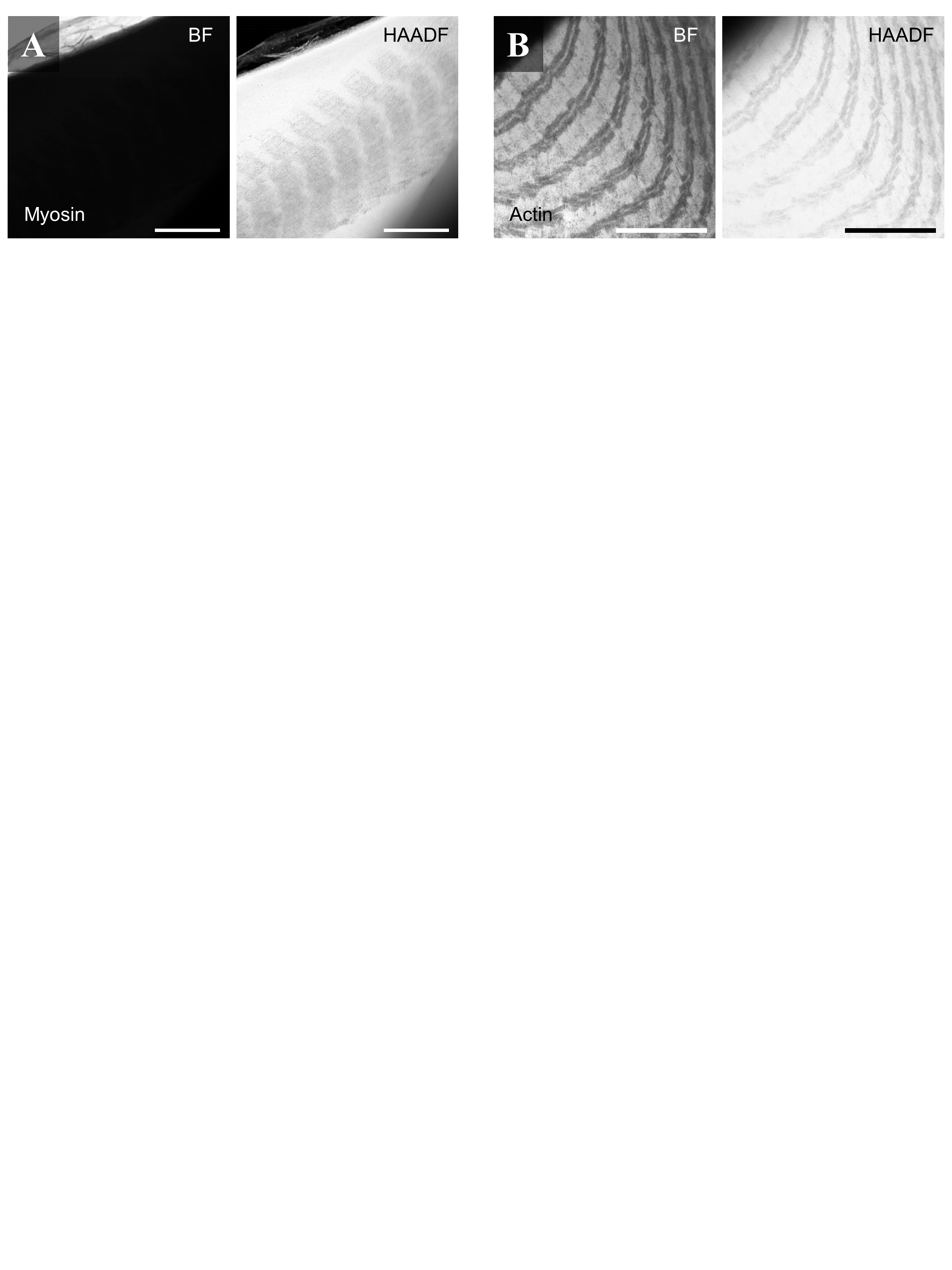


**Fig**. **S40**. **HAADF inversion phenomena in STEM imaging in meat slice samples.** Periodic patterned AgNPs against (**A**) myosin and (**B**) actin proteins *via* CamBio from pork shoulder meat slice substrates (left: BF, right: HAADF images). Scale bar. 5 μm.

**
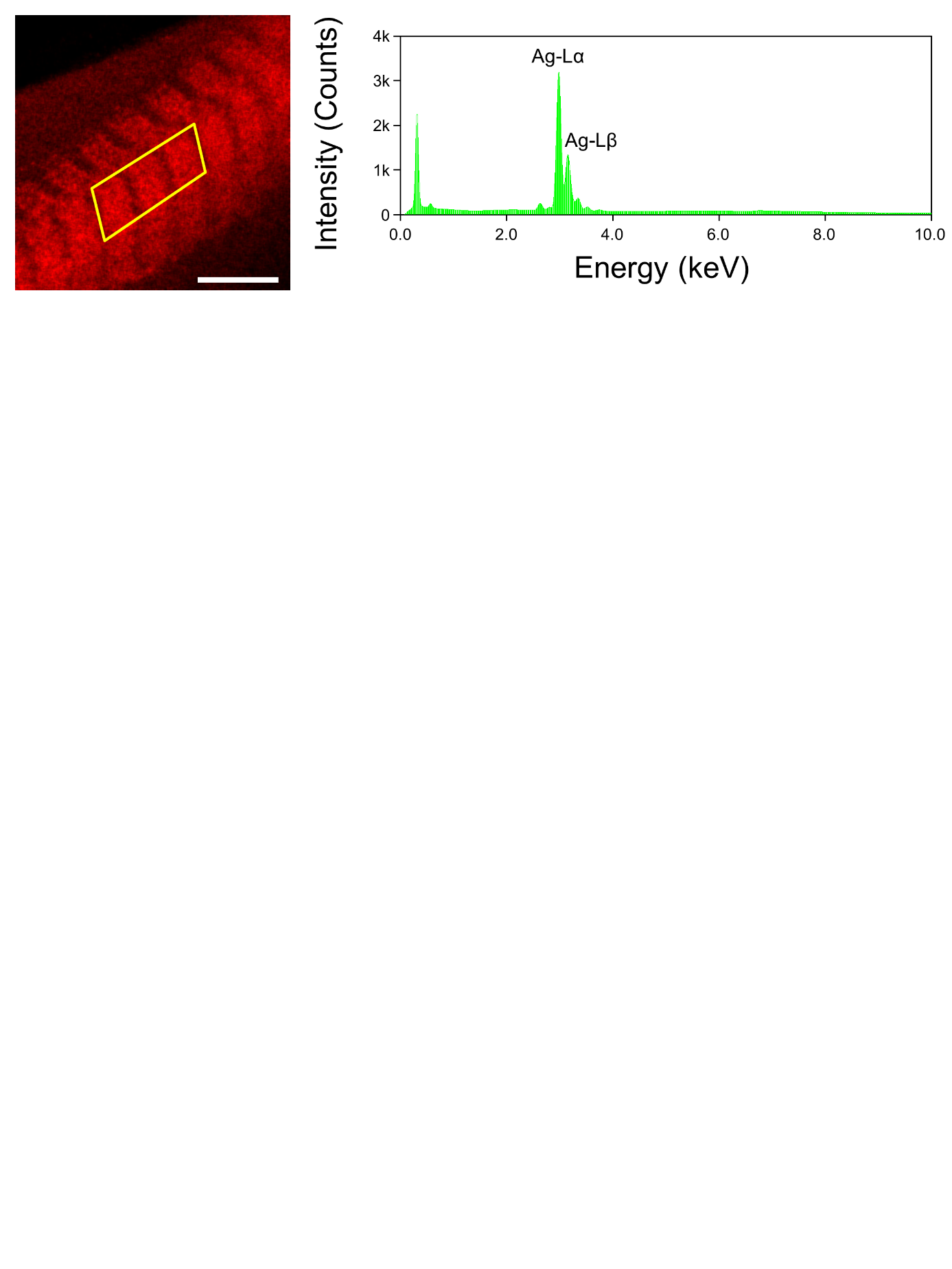
**

**Fig**. **S41**. **EDS analysis in periodic patterned AgNPs against myosin proteins**. EDS spectrum obtained from the yellow boxed region in the left EDS mapping image. Scale bar. 5 μm.

**
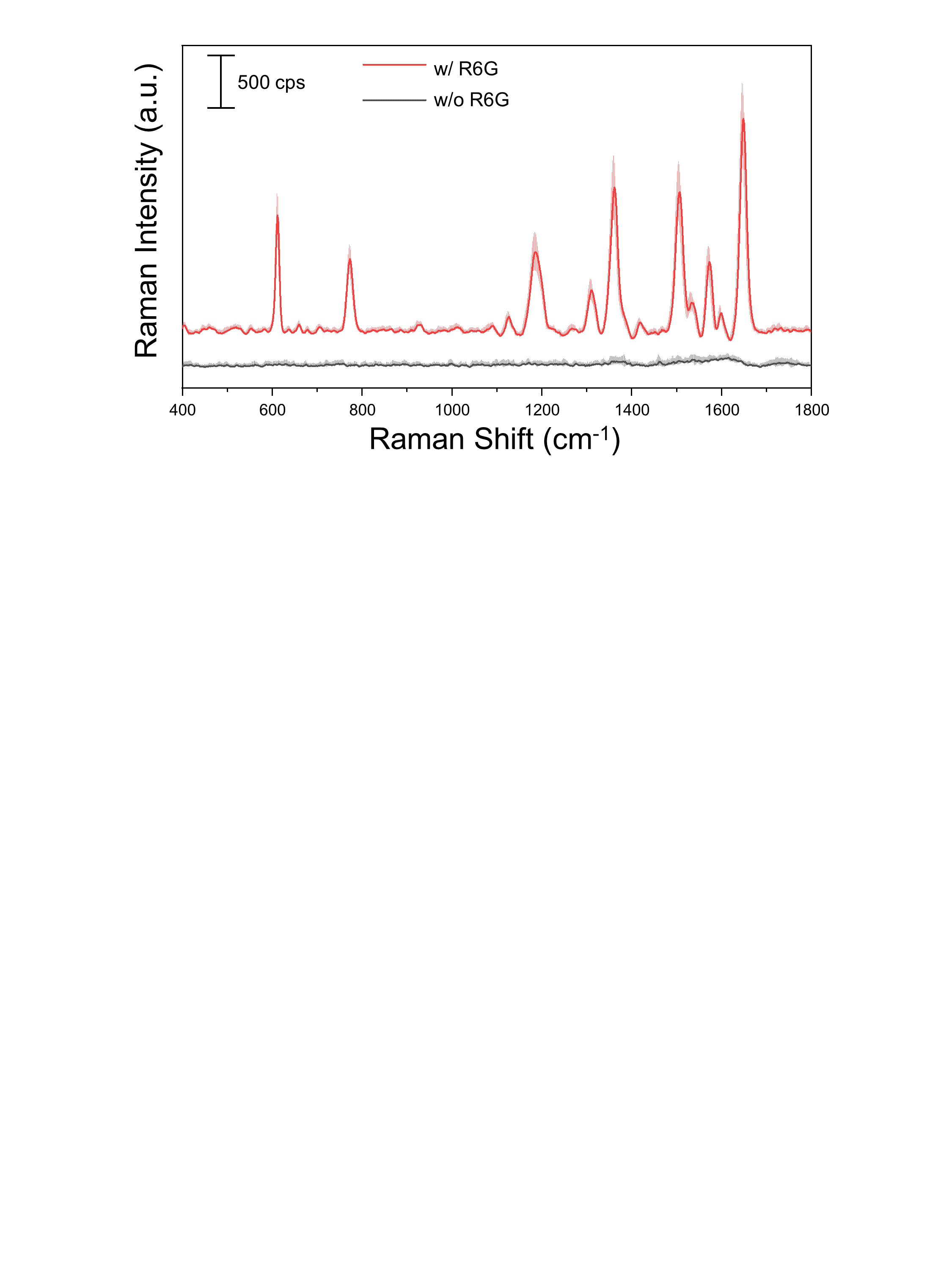
**

**Fig**. **S42**. **Raman and SERS spectra of R6G recorded on silver-grown meat slice substrate (target protein: myosin).** Data of each sample are presented as mean ± s.d., *n* = 7 independent points in each case in a single substrate.


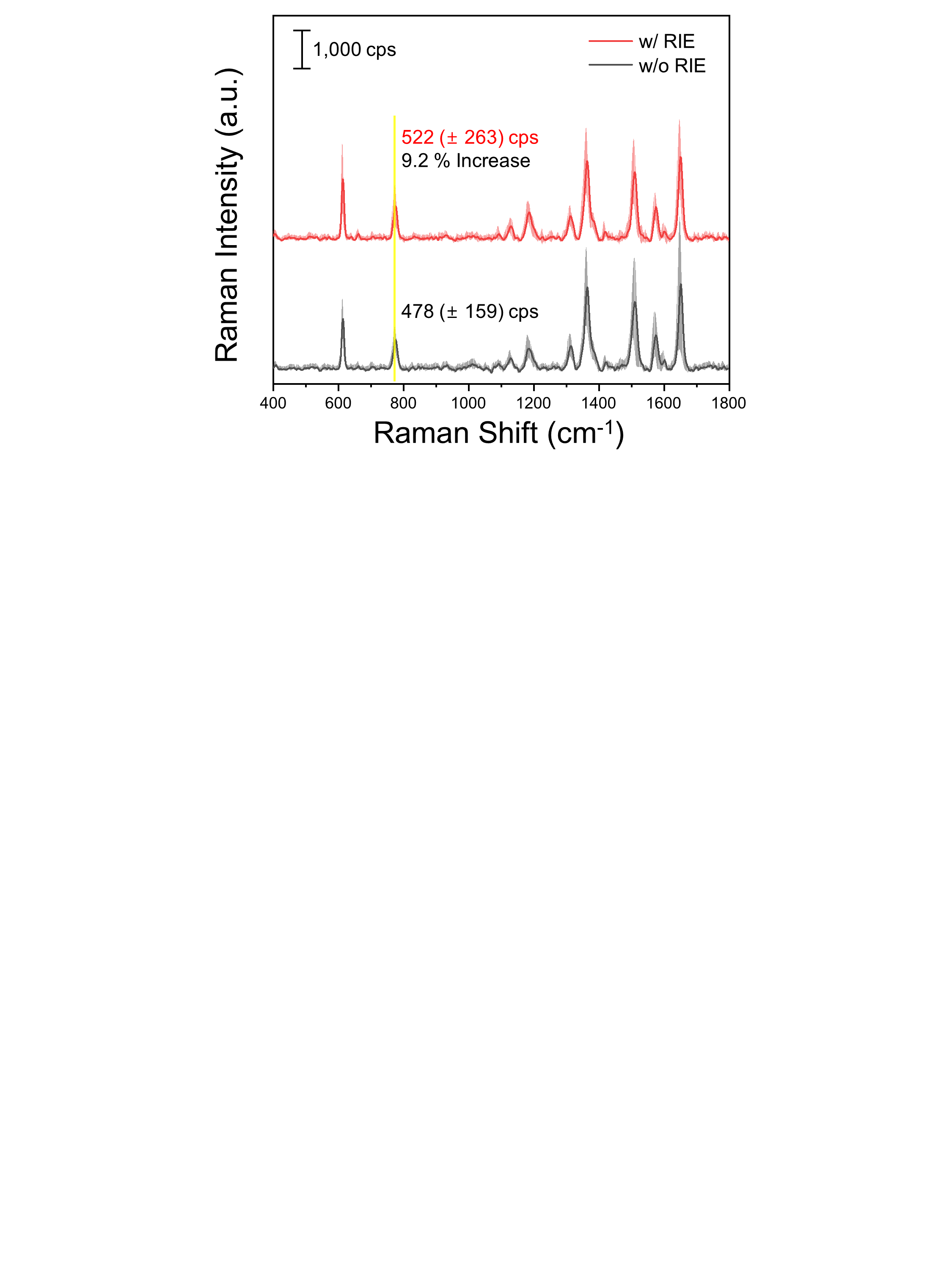


**Fig**. **S43**. **Average SERS spectra of R6G recorded on silver-grown meat slice substrates with and without the RIE process (target protein: myosin).** Data for each sample are presented as mean ± s.d., *n* = 10 taken from independent 5 points from two substrates in each case.

**
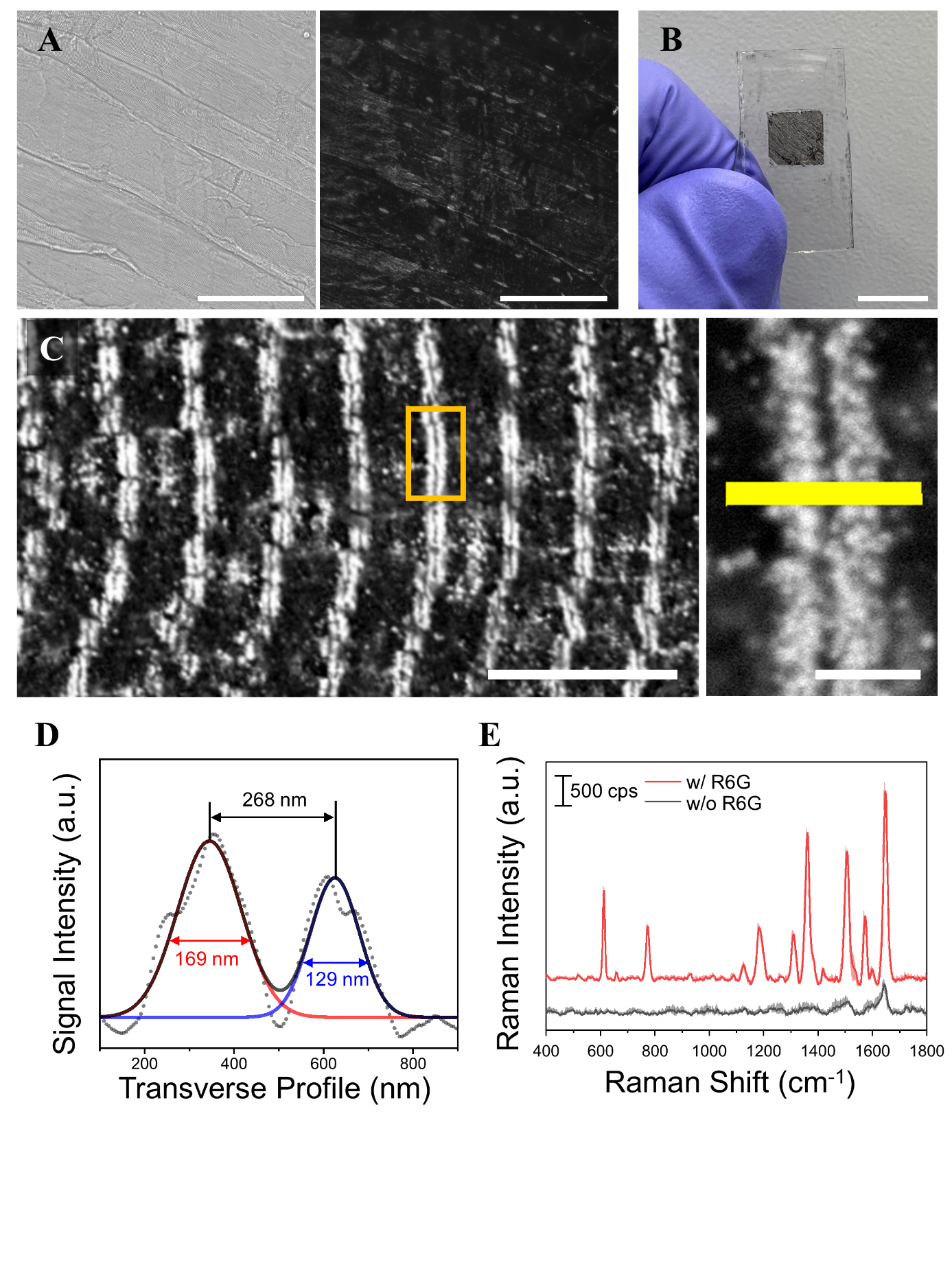
**

**Fig**. **S44**. **AgNPs against actin protein structures in a slice meat substrate (pork shoulder) *via* CamBio.** (**A**) BF images of the pre-and post-silver-grown meat slice substrate in which actin proteins were labeled. (**B**) Digital image of silver-grown meat slice substrate. (**C**) SEM images of periodic patterned AgNPs against actin proteins. (right image: a magnified SEM image in the yellow boxed region). (**D**) The SEM signal profile along the yellow line in C. (**E**) Raman and SERS spectra of R6G was recorded on a silver-grown meat slice substrate (target protein: actin). Data of each sample are presented as mean ± s.d., *n* = 5 independent points in each case in a single substrate. Scale bar. A. 150 μm, B. 1 cm, C. 5 μm (left), 500 nm (right).

**
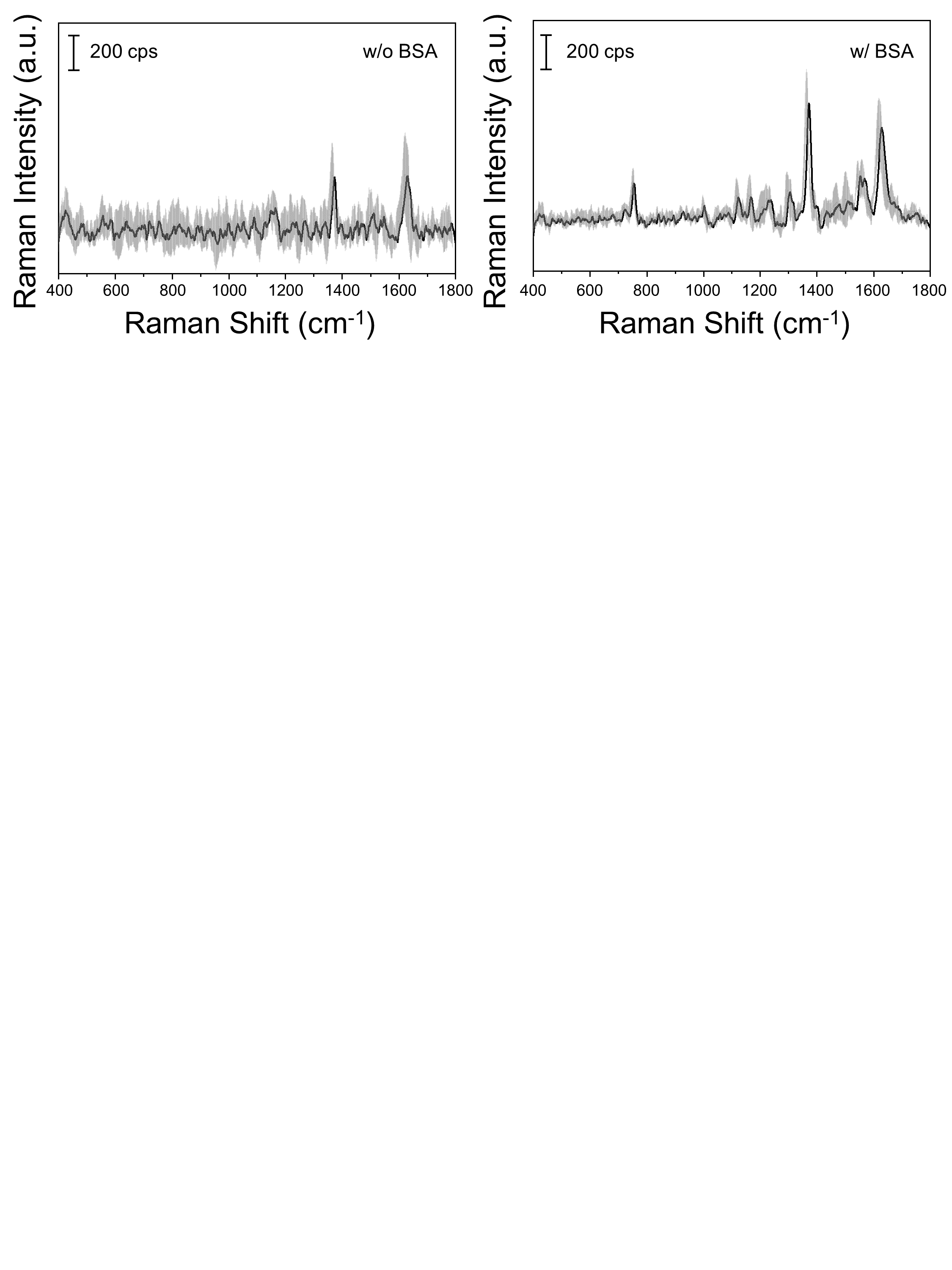
**

**Fig**. **S45**. **Average Raman and SERS spectrum of BSA recorded on silver-grown meat slice substrate (target protein: myosin).** Data of each sample are presented as mean ± s.d., *n* = 7 independent points in each case in a single substrate.

**
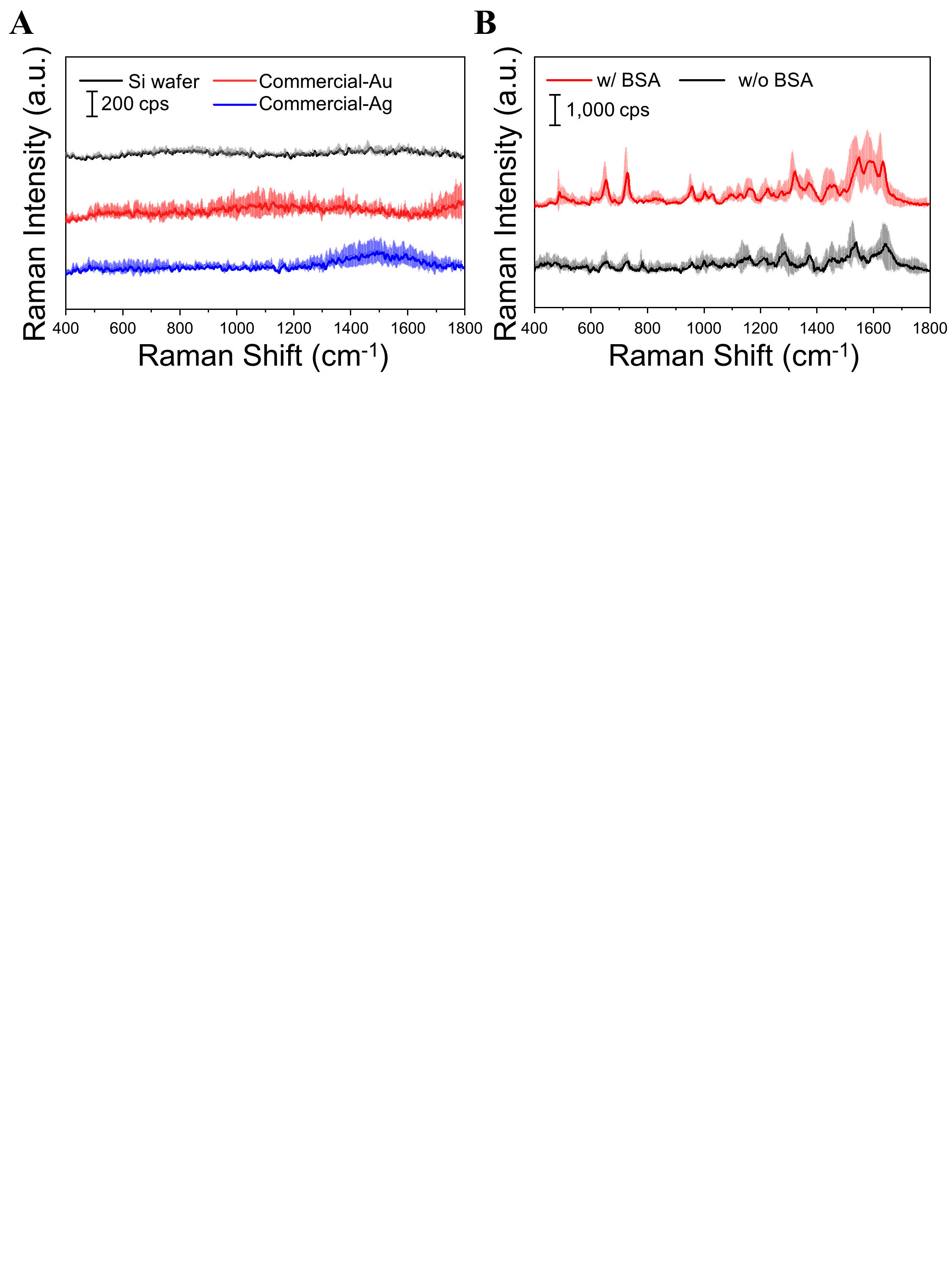
**

**Fig**. **S46**. **Average Raman and SERS spectra of BSA were recorded on different substrates.** (**A**) Raman spectrum of BSA was recorded on a silicon wafer, and SERS spectra of BSA were recorded on commercial SERS substrates. Data of each sample are presented as mean ± s.d., *n* = 3 independent points. (**B**) Raman and SERS spectra of BSA were recorded on a silver-grown cell substrate (target protein: microtubule). Data of each sample are presented as mean ± s.d., *n* = 7 independent points in each case in a single substrate.

**
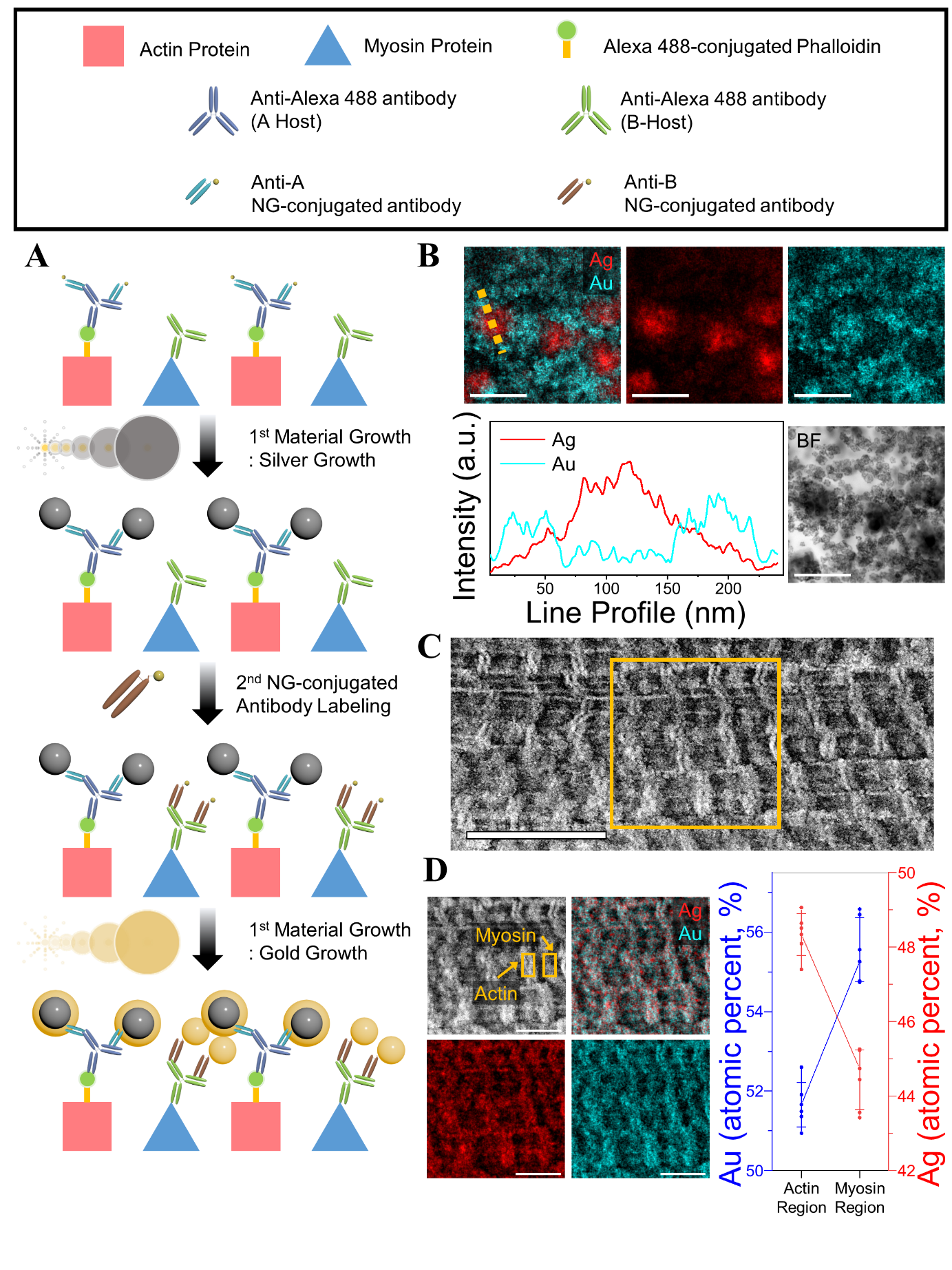
**

**Fig**. **S47**. **Successive labeling and material growth strategy.** (**A**) Schematic of successive labeling and material growth in a muscle tissue. (**B**) Gold toning results on the meat slice substrate, silver-grown at actin proteins, and successively labeled on myosin proteins. (**C**) Inverted HAADF image of the multi-material grown meat slice sample. (**D**) STEM, EDS mapping images in yellow boxed region in C, and an atomic percentage comparison at actin and myosin regions in yellow boxed regions in the merged EDS mapping image. Data are presented as mean ± s.d., *n* = 5 independent regions in a single substrate. Scale bar. B. 200 nm, C. 5 μm, D. 2 μm.

Table S1. Comparison of biotemplating methods based on specificity and tunability.

| **Biotemplating Methods** | **Protein Specificity** | **Sample Tunability** | **Reference** |
| --- | --- | --- | --- |
| Recombinant-Protein Based | △ | △ | S1, S2, S3 |
| Microbial Biotemplates | X | X | S4, S5 |
| Phage-Display Technique Based | O | △ | S6, S7, S8 |
| Polymer-Assisted  Cell Metalization  (PACM) | X | △ | S9 |
| CamBio  (Labeled-Biostructure) | O | O | This work  S10, S11 |

O: Possible / △: Partially Possible / X: Impossible

**Reference**

S1 A. V. Cresce, J. S. Silverstein, W. E. Bentley, P. Kofinas, *Macromolecules* **2006**, *39*, 5826.

S2 G. He, T. Dahl, A. Veis, A. George, *Nat. Mater.* **2003**, *2*, 552.

S3 S. M. Bird, O. El-Zubir, A. E. Rawlings, G. J. Leggett, S. S. Staniland, *J. Mater. Chem. C* **2016**, *4*, 3948.

S4 H. Zhou, T. Fan, D. Zhang, Q. Guo, H. Ogawa, *Chem. Mater.* **2007**, *19*, 2144.

S5 C. Zhu, F. Wan, H. Ping, H. Wang, W. Wang, Z. Fu, *J. Power Sources* **2021**, *506*, 230180.

S6 S. W. Lee, C. Mao, C. E. Flynn, A. M. Belcher, *Science (80-. ).* **2002**, *296*, 892.

S7 S. W. Lee, S. K. Lee, A. M. Belcher, *Adv. Mater.* **2003**, *15*, 689.

S8 K. T. Nam, D. Kim, P. J. Yoo, C. Chiang, N. Meethong, P. T. Hammond, Y. Chiang, A. M. Belcher, *Science (80-. ).* **2006**, 885.

S9 W. Wang, Q. Gan, Y. Zhang, X. Lu, H. Wang, Y. Zhang, H. Hu, L. Chen, L. Shi, S. Wang, Z. Zheng, *Adv. Mater.* **2021**, *33*, 1.

S10 C. W. Song, D. H. Song, D. G. Kang, K. H. Park, C. E. Park, H. Kim, Y. Hur, S. D. Jo, Y. S. Nam, J. Yeom, S. M. Han, J. B. Chang, *Adv. Mater.* **2022**, *34*, 1.

S11 C. W. Song, J. Ahn, I. Yong, N. Kim, C. E. Park, S. Kim, S.-Y. Chung, P. Kim, I.-D. Kim, J.-B. Chang, *Adv. Sci.* **2023**, *10*, 2302830.

Table S2. Chemicals and materials list

| **Product name** | **Vendor** | **Product number** |
| --- | --- | --- |
| **Cell culture and fixation** | | |
| BS-C-1 Cell | Korean Cell Line Bank | 10026 |
| HeLa Cell | Korean Cell Line Bank | 10002 |
| CS16-CultureWell Removable Chambered Coverglass | Grace Bio-Labs | 112358 |
| Grace Bio-Labs CultureWell chambered coverglass | Grace Bio-Labs | 103380 |
| 20Ø Confocal dish | SPL Life Science | 101350 |
| Microscope Cover Glass Circular, 12 mm | MARIENFELD | HSU-0111520 |
| Falcon 35 mm TC-treated Easy-Grip Style Cell Culture Dish | CORNING | 353001 |
| Cell cultrue dish (60 mm) | SPL Life Science | 20060 |
| Cell cultrue dish (90 mm) | SPL Life Science | 20100 |
| Cell cultrue dish (150 mm) | SPL Life Science | 20151 |
| µ-Slide 18 Well - Flat ibiTreat | ibidi | 81826 |
| Minimum essential medium (MEM) | Thermofisher | 11095114 |
| Fetal bovine serum (FBS) | Thermofisher | 10082147 |
| Penicillin-streptomycin | Thermofisher | 15140122 |
| Sodium pyruvate | Thermofisher | 11360070 |
| 10× PBS | Invitrogen | AM9625 |
| 0.1 M 1,4-piperazinediethanesulfonic acid (PIPES) | Sigma | P1851 |
| Sodium hydroxide (NaOH) | Sigma | S8045 |
| Magnesium chloride (MgCl_2_) | Sigma | M1028 |
| Ethylene glycol-bis(2-aminoethylether)-N,N,N′,N′-tetraacetic acid (EGTA) | Sigma | E3889 |
| Triton X-100 | Sigma | X100 |
| 16% paraformaldehyde (PFA) | Electron Microscopy Science | 15710 |
| Glutaraldehyde (GA) | Electron Microscopy Science | 16019 |
| Sodium borohydride (NaBH_4_) | Sigma | S9640 |
| Glycine | Sigma | 50046 |
| **Cell patterning and culture** | | |
| Confocal Dish (Insert Type) | SPL Life Science | 100351 |
| Polydimethylsiloxane (PDMS) | DOW | SYLGARD 184 silicon elastomer kit |
| Dopamine hydrochloride | Sigma | H8502 |
| Tris-HCl buffer (pH 8.5) | Biosesang | TR2016-000-85 |
| 40% Acrylamide solution | BIO-RAD | 1610140 |
| 2% N, N'-methylenebisacrylamide (BIS) | BIO-RAD | 1610142 |
| Sulfo-SANPAH | ProteoChem | c1111 |
| HEPES solution | Sigma | H0887 |
| Collagen type I solution (PureCol, 3mg/ml) | Advanced Biomatrix | 5005 |
| DPBS | WELGENE | LB 001-02 |
| C2C12 Cell | ATCC | CRL-1772 |
| Dulbecco's Modified Eagle’s Medium (DMEM) | WELGENE | LM 001-05 |
| Fetal bovine serum (FBS) | WELGENE | S001-01 |
| Penicillin-streptomycin | WELGENE | LS202-02 |
| Horse serum | WELGENE | S004-01 |
| **Staining of cells and meat slices** | | |
| Normal donkey serum | JacksonImmunoResearch | 017-000-121 |
| Normal goat serum | JacksonImmunoResearch | 055-000-121 |
| MAXblock Blocking Medium | Active Motif | 15252 |
| MAXbind Staining Medium | Active Motif | 15253 |
| MAXwash Washing Medium | Active Motif | 15254 |
| Alexa Fluor 488 Phalloidin | Invitrogen | A12379 |
| Pork Shoulder | Jeongyookgak | - |
| Beef Brisket | Jeongyookgak | - |
| Chicken Breast | Jeongyookgak | - |
| https://www.jeongyookgak.com/index | | |
| Sucrose | Sigma | 84097 |
| Peel-A-Way embedding mold | Sigma | E6032-1CS |
| Tissue-Tek O.C.T Compound | SKURA | 4583 |
| High-Profile Disposable Blades 818 | LEICA | 14035838926 |
| Silane-Prep Slides | Sigma | S4651 |
| iSpacer 3mm, Double-sided sticky, Rectangular well | Sunjin Labs | IS013 |
| Stain Tray staining system | Simport | HSI-M922-2 |
| Collagenase, Type IV, powder | Thermofisher | 17100-019 |
| Calcium chloride (CaCl_2_) | Sigma | C1016 |
| Hanks' Balanced Salt Solution (HBSS) (10×) | Thermofisher | 14065056 |
| **Protein-retention expansion microscopy (Pro-ExM)** | | |
| Acryloyl-X SE (AcX) | Thermofisher | A-20770 |
| Sodium acrylate | Sigma | 408220 |
| Acrylamide | Sigma | A9099 |
| N, N'-methylenebisacrylamide (BIS) | Sigma | M7279 |
| Sodium chloride | Sigma | 71376 |
| Ammonium persulfate (APS) | Sigma | A3678 |
| N,N,N',N'-tetramethylethylenediamide (TEMED) | Sigma | T7024 |
| 4-hydroxy-TEMPO (H-TEMPO) | Sigma | 176141 |
| Proteinase K | New England Biolabs | P8107S |
| Ethylenediaminetetraacetic acid (EDTA) | Sigma | EDS |
| Trizma hydrochloride (Tris-HCl), pH8.0, 1 M | Sigma | T3038 |
| DAPI | Sigma | D9542 |
| Atto 565 NHS ester | Sigma | 72464 |
| Sodium bicarbonate | Sigma | S6297 |
| **Material Growth** | | |
| Sodium citrate tribasic dihydrate | Sigma | S4641 |
| Silver Enhancement Kit | Nanoprobes | HQ Silver |
| Gum arabic from acacia tree | Sigma | 51198 |
| 4-(2-Hydroxyethyl)piperazine-1-ethanesulfonic acid (HEPES) | Sigma | H3375 |
| Hydroquinone | Sigma | H9003 |
| Silver lactate | Sigma | 359750 |
| Triton X-100 | Sigma | X100 |
| Gold Enhancement Kit | Nanoprobes | GoldEnhance EM |
| **Biocompatibility Test** | | |
| Live/Dead Cell Imaging Kit | Invitrogen | R37601 |
| **Ultramicrotome Sectioning** | | |
| Ethyl alcohol, Pure | Sigma | E7023 |
| Propylene Oxide, EM grade | EMS | 20401 |
| Embed 812 Embedding Kit with DMP-30 | EMS | 14120 |
| Holey Carbon Supported Copper Gids | Sigma | TEM-HC300CU100 |
| **Raman Spectroscopy** | | |
| Ethyl alcohol, Pure | Sigma | E7023 |
| Rhodamine 6G | Supelco | 56226 |
| Bovine Serum Albumin (BSA) | VWR | 0332-100G |
| **Aptamer-Functionalization and BPA Incubation** | | |
| Salmon Sperm DNA (sheared) (10 mg/mL) | Invitrogen | AM9680 |
| Potassium chloride | Sigma | P9541 |
| Dimethyl sulfoxide (DMSO) | Sigma | 276855 |
| Bisphenol A (BPA) | Sigma | 239658 |

Table S3.Antibody list

| **No.** | **Antibody** | **Primary or Secondary** | **Vendor** | **Catalog** | **Host** | **Clonality** | **Conjugate** | **Dilution (μg/ml)** |
| --- | --- | --- | --- | --- | --- | --- | --- | --- |
| 1 | Anti-beta Tubulin | Primary | Abcam | ab6046 | Rabbit | Poly | Unconjugated | 1 |
| 2 | Anti-alpha Tubulin | Primary | Sigma | T5168 | Mouse | Mono | Unconjugated | 1 |
| 3 | Anti-Myosin heavy chain (MYH1E gene) | Primary | DSHB | MF-20 | Mouse | Mono | Unconjugated | 1.25 |
| 4 | Anti-Myosin heavy chain (MYH2 gene) | Primary | DSHB | SC-71 | Mouse | Mono | Unconjugated | 1.25 |
| 5 | Anti-Myosin heavy chain (MYH7 gene) | Primary | DSHB | BA-D5 | Mouse | Mono | Unconjugated | 1.25 |
| 6 | Aexa Fluor 488 | Primary | Invitrogen | A-11094 | Rabbit | Poly | Unconjugated | 10 |
| 7 | Goat anti-Rabbit IgG (H+L) Highly Cross-Adsorbed | Secondary | Invitrogen | A-11034 | Goat | Poly | Alexa Fluor 488 | 4 |
| 8 | FluoroNanogold Fab' Goat anti-Rabbit IgG (H+L) | Secondary | Nanoprobes | 7204 | Goat | - | 1.4 nm Nanogold, Alexa Fluor 488 | 2 |
| 9 | FluoroNanogold Fab' Goat anti-Rabbit IgG (H+L) | Secondary | Nanoprobes | 7404 | Goat | - | 1.4 nm Nanogold, Alexa Fluor 546 | 2 |
| 10 | FluoroNanogold Fab' Goat anti-Rabbit IgG (H+L) | Secondary | Nanoprobes | 7504 | Goat | - | 1.4 nm Nanogold, Alexa Fluor 647 | 2 |
| 11 | FluoroNanogold Fab' Goat anti-Mouse IgG (H+L) | Secondary | Nanoprobes | 7202 | Goat | - | 1.4 nm Nanogold, Alexa Fluor 488 | 2 |
| 12 | FluoroNanogold Fab' Rabbit anti-Goat IgG (H+L) | Secondary | Nanoprobes | 7506 | Rabbit | - | 1.4 nm Nanogold, Alexa Fluor 647 | 2 |
| 13 | FluoroNanogold Fab' Goat anti-Mouse IgG (H+L) | Secondary | Nanoprobes | 7502 | Goat | - | 1.4 nm Nanogold, Alexa Fluor 647 | 2 |
| 14 | Nanogold-Fab' Goat anti-Rabbit IgG (H+L) | Secondary | Nanoprobes | 2004 | Goat | - | 1.4 nm Nanogold | 2 |
| 15 | Nanogold-Fab' Goat anti-Mouse IgG (H+L) | Secondary | Nanoprobes | 2002 | Goat | - | 1.4 nm Nanogold | 2 |
| 16 | Nanogold-IgG Goat anti-Rabbit IgG (H+L) | Secondary | Nanoprobes | 2003 | Goat | - | 1.4 nm Nanogold | 2 |
| 17 | Nanogold-IgG Rabbit anti-Goat IgG (H+L) | Secondary | Nanoprobes | 2005 | Rabbit | - | 1.4 nm Nanogold | 2 |
| 18 | Rabbit anti-Goat IgG (H+L) Superclonal Recombinant | Secondary | Invitrogen | A27011 | Rabbit | Poly | Unconjugated | 2 |

Table S4.Imaging systems and conditions

| **No.** | **Type** | **Microscope** | **Figure** | **Used antibodies and fluorophores (FM) metal nanoparticles (BF / EM) with imaging conditions** |
| --- | --- | --- | --- | --- |
| 1 | FM | Andor dragonfly | Figure. 1A, movie. S1 | Nucleus (DAPI), β-tubulin (Alexa 488), Unbiased labeling (ATTO565) Objective: 60× / Laser (intensity, exposure time): 445 (40 %, 400 ms), 521 (40 %, 400 ms), 594 (20 %, 200 ms) |
| 2 | BF | Ti2-E | Figure. S2 | β-tubulin (Silver) |
| 3 | FM | Andor dragonfly | Figure. S3 | β-tubulin (Alexa 546) Objective: 10× / Laser (intensity, exposure time): 594 (25 %, 250 ms) |
| 4 | FM | Andor dragonfly | Figure. S4, 5 | β-tubulin (Alexa 488, Alexa 546, Alexa 647) Objective: 40× / Laser (intensity, exposure time): 521 (10 %, 200 ms), 594 (25 %, 250 ms), 698 (35 %, 200 ms) |
| 5 | SEM | SU-8230 (BSE) | Figure. 1C, Figure. S7 | α-tubulin (Silver) |
| 6 | STEM | Titan cubed G2 60 | Figure. 1D, Figure. S13 | β-tubulin (Silver (Fig. 1D) / Gold (Supple. Fig. F)) / 300 nm thickness section Imaging with energy-dispersive X-ray spectroscopy (Super-X detector) |
| 7 | BF | uEye UI-146xLE Series | Figure. S9 | β-tubulin (Silver) |
| 8 | BF | uEye UI-146xLE Series | Figure. S10 | β-tubulin (Silver) |
| 9 | BF | Ti2-E | Figure. S12 | β-tubulin (Silver) and without labeling |
| 10 | SEM | S-4800 | Figure. S12 | Without labeling |
| 11 | FM | Andor dragonfly | Figure. S13 | β-tubulin (Alexa 546) Objective: 40× / Laser (intensity, exposure time): 594 (30 %, 300 ms) |
| 12 | BF | Ti2-E | Figure. S13 | β-tubulin (Gold) |
| 13 | SEM | SU-8230 (BSE) | Figure. S13 | β-tubulin (Gold) |
| 14 | FM | Andor dragonfly | Figure. S14 | Live/Dead Cell Imaging Kit  Objective: 10× / Laser (intensity, exposure time): 445 (30 %, 200 ms), 521 (20 %, 200 ms) |
| 15 | BF | BX51WI |  | Cells with the fixed, and material-grown substrates / α-tubulin (Silver, Gold) |
| 16 | SEM | SU-8230 | Figure. 2B, Figure. S16 | β-tubulin (Silver) |
| 17 | STEM | Titan cubed G2 60 | Figure. 2B, Figure. S15 | β-tubulin (Silver) / 300 nm thickness section Imaging with energy-dispersive X-ray spectroscopy (Super-X detector) |
| 18 | FM | Andor dragonfly | Figure. S18 | β-tubulin (Alexa 546 / Alexa 647) Objective: 40× / Laser (intensity, exposure time): 594 (30 %, 300 ms), 698 (20 %, 200 ms) |
| 19 | BF | Ti2-E | Figure. S19 | β-tubulin (Silver) |
| 20 | SEM | SU-8230 | Figure. S20 | β-tubulin (Silver) |
| 21 | FM | Nikon C2 Plus | Figure. S29 | BPA Aptamer Complementary DNA - Atto 647N Objective: 40× / Laser (intensity, gain): 640 (5, 10) |
| 22 | BF | BX51WI | Figure. 4A | patterned cell (without labeling) |
| 23 | FM | Nikon C2 Plus | Figure. 4B | β-tubulin (Alexa 546) Objective: 10× / Laser (intensity, gain): 557 (3, 10) Objective: 40× / Laser (intensity, gain): 557 (7.5, 10) |
| 24 | BF | BX51WI |  | patterned cell (Silver) |
| 25 | FM | Andor dragonfly | Figure. S32, movie. S2, and S3 | β-tubulin (Alexa 647) Objective: 60× / Laser (intensity, exposure time): 698 (25 %, 300 ms) |
| 26 | SEM | SU-8230 (BSE) | Figure. 4C | β-tubulin (Silver) |
| 27 | SEM | SU-8230 (SE / BSE) | Figure. S33 | β-tubulin (Silver) |
| 28 | BF | UI-1460LE-C-HQ | Figure. S34 | β-tubulin (Silver) |
| 29 | FM | Nikon C2 Plus | Figure. S36 | β-tubulin (Alexa 546) Objective: 10× / Laser (intensity, gain): 557 (5, 10) |
| 30 | BF | BX51WI |  | β-tubulin (Silver) |
| 31 | SEM | SU-8230 (BSE) |  |  |
| 32 | FM | Nikon C2 Plus | Figure. S37 | Actin (Alexa 488) / Mysosin (Alexa 647) Objective: 40× / Laser (intensity, gain): 488 (1.5, 6), 640 (4, 10) |
| 33 | SEM | SU-8230 (BSE) |  | Myosin (Silver) |
| 34 | FM | Nikon C2 Plus | Figure. 5C, Figure. S39 | Actin (Alexa 488) / Mysosin (Alexa 647) Objective: 40× / Laser (intensity, gain): 488 (1.5, 6), 640 (5, 15) |
| 35 | STEM | Talos F200X | Figure. 5D, E, Figure. S40, 41, and 47 | Myosin, Actin (Silver) BF, STEM (HAADF), EDS |
| 36 | SEM | S-4800 | Figure. 5F, G | Myosin (Silver) |
| 37 | BF | BX51WI | Figure. S44A | Actin (Silver) |
| 38 | SEM | SU-8230 (BSE) | Figure. S44C |  |

Table S5. BPA-specific aptamer and complementary ssDNA sequences were used in this work.

| **ssDNA #1** | **ss DNA Sequence #1 (5’-SH-(CH_2_)_6_-aptamer-3’), 63 NT** |
| --- | --- |
| BPA target aptamer | CCGGTGGGTGGTCAGGTGGGATAGCGTTCCGCGTATGGCCCAGCGCATCAGGGGTTCGCACCA |
| **ssDNA #2** | **ss DNA Sequence #2 (5’-Complementary-3’-T5-ATTO647N), 30 NT** |
| Complementary ssDNA | GAACGCTATCCCACCTGACCACCCATTTTT |
